# A simulation study on the role of mitochondria‐sarcoplasmic reticulum Ca^2+^ interaction in cardiomyocyte energetics during exercise

**DOI:** 10.1113/JP286054

**Published:** 2024-10-10

**Authors:** Ayako Takeuchi, Satoshi Matsuoka

**Affiliations:** ^1^ Department of Integrative and Systems Physiology, Faculty of Medical Sciences and Life Science Innovation Center University of Fukui Fukui Japan

**Keywords:** energetics, exercise, heart, mathematical modelling, mitochondria, sarcoplasmic reticulum

## Abstract

**Abstract:**

Previous studies demonstrated that the mitochondrial Ca^2+^ uniporter MCU and the Na^+^‐Ca^2+^ exchanger NCLX exist in proximity to the sarcoplasmic reticulum (SR) ryanodine receptor RyR and the Ca^2+^ pump SERCA, respectively, creating a mitochondria‐SR Ca^2+^ interaction. However, the physiological relevance of the mitochondria‐SR Ca^2+^ interaction has remained unsolved. Furthermore, although mitochondrial Ca^2+^ has been proposed to be an important factor regulating mitochondrial energy metabolism, by activating NADH‐producing dehydrogenases, the contribution of the Ca^2+^‐dependent regulatory mechanisms to cellular functions under physiological conditions has been controversial. In this study, we constructed a new integrated model of human ventricular myocyte with excitation‐contraction‐energetics coupling and investigated systematically the contribution of mitochondria‐SR Ca^2+^ interaction, especially focusing on cardiac energetics during dynamic workload transitions in exercise. Simulation analyses revealed that the spatial coupling of mitochondria and SR, particularly via mitochondrial Ca^2+^ uniport activity‐RyR, was the primary determinant of mitochondrial Ca^2+^ concentration, and that the Ca^2+^‐dependent regulatory mechanism facilitated mitochondrial NADH recovery during exercise and contributed to the stability of NADH in the workload transition by about 40%, while oxygen consumption rate and cytoplasmic ATP level were not influenced. We concluded that the mitochondria‐SR Ca^2+^ interaction, created via the uneven distribution of Ca^2+^ handling proteins, optimizes the contribution of the mitochondrial Ca^2+^‐dependent regulatory mechanism to stabilizing NADH during exercise.

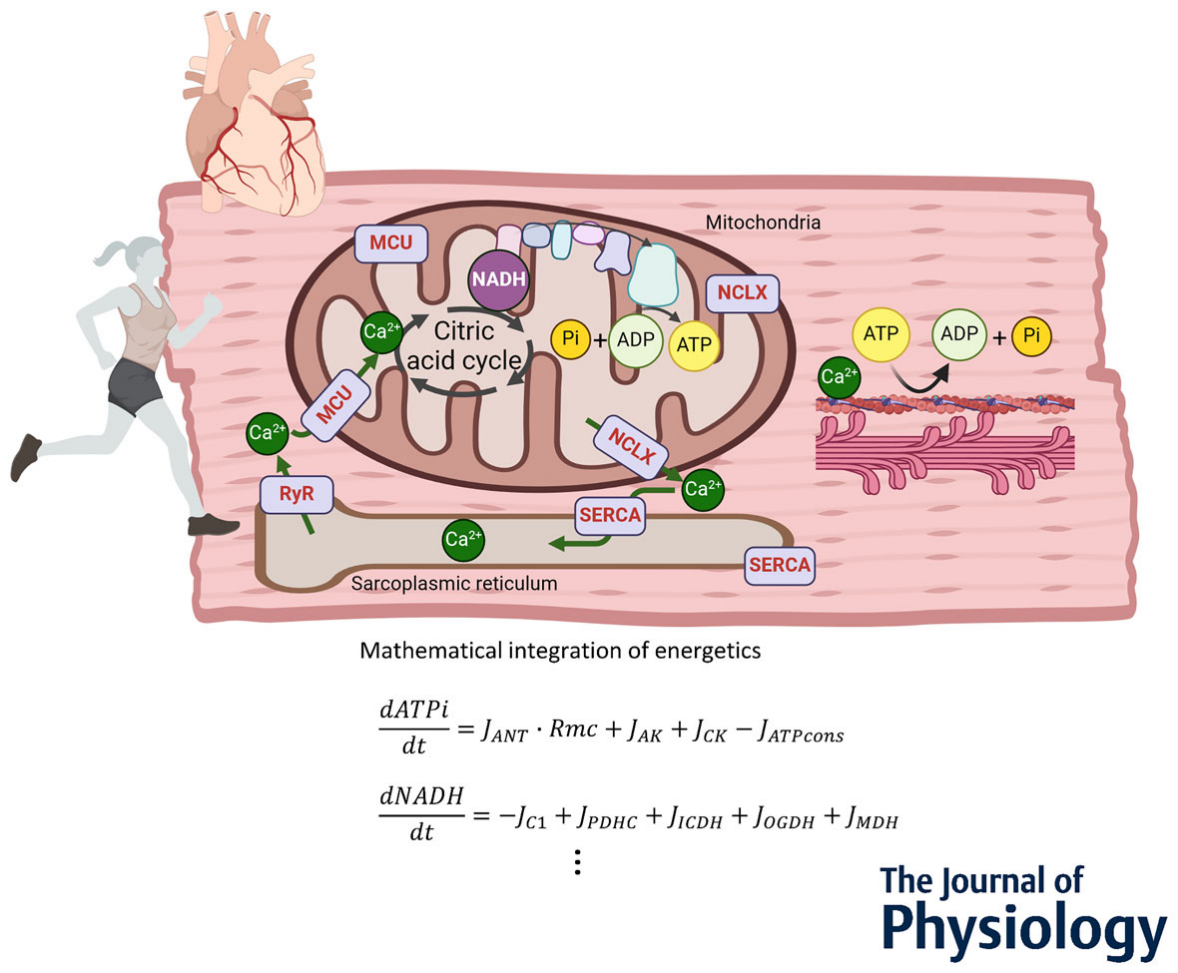

**Key points:**

- The mitochondrial Ca^2+^ uniporter protein MCU and the Na^+^‐Ca^2+^ exchanger protein NCLX are reported to exist in proximity to the sarcoplasmic reticulum (SR) ryanodine receptor RyR and the Ca^2+^ pump SERCA, respectively, creating a mitochondria‐SR Ca^2+^ interaction in cardiomyocytes.
- Mitochondrial Ca^2+^ (Ca^2+^_mit_) has been proposed to be an important factor regulating mitochondrial energy metabolism, by activating NADH‐producing dehydrogenases.
- Here we constructed an integrated model of a human ventricular myocyte with excitation‐contraction‐energetics coupling and investigated the role of the mitochondria‐SR Ca^2+^ interaction in cardiac energetics during exercise.
- Simulation analyses revealed that the spatial coupling particularly via mitochondrial Ca^2+^ uniport activity‐RyR is the primary determinant of Ca^2+^_mit_ concentration, and that the activation of NADH‐producing dehydrogenases by Ca^2+^_mit_ contributes to NADH stability during exercise.
- The mitochondria‐SR Ca^2+^ interaction optimizes the contribution of Ca^2+^_mit_ to the activation of NADH‐producing dehydrogenases.

## Graphical abstract

**Abstract figure legend** An integrated model of human ventricular myocyte with excitation‐contraction‐energetics coupling was created to systematically analyse the contribution of Ca^2+^ regulation of mitochondrial enzymes to cardiomyocyte functions during workload transition; i.e. exercise. During exercise, the mitochondria‐sarcoplasmic reticulum Ca^2+^ interaction, via uneven distributions of the mitochondrial Ca^2+^ uniporter protein MCU, Na^+^‐Ca^2+^ exchanger protein NCLX, sarcoplasmic reticulum ryanodine receptor RyR and Ca^2+^ pump SERCA, optimizes the homeostasis of NADH, a key intermediate connecting mitochondrial metabolism and oxidative phosphorylation.

## Introduction

Mitochondrial Ca^2+^ (Ca^2+^_mit_), which dynamically changes in response to changes in the cellular activity, is an important factor for cellular energy metabolism, apoptosis and cytoplasmic Ca^2+^ (Ca^2+^_cyt_) signalling (see reviews by Brown et al., 2017; O'Rourke et al., 2021). In cardiomyocytes, this Ca^2+^_mit_ dynamics is determined by Ca^2+^ influx via mitochondrial Ca^2+^ uniport (CaUni) activity and Ca^2+^ efflux via mitochondrial Na^+^‐Ca^2+^ exchange (NCX_mit_) and H^+^‐Ca^2+^ exchange (HCX_mit_) activities, with the former playing a dominant role (Carafoli et al., 1974; Nicholls & Crompton, 1980). In the past 15 years, the genes responsible for these activities have been identified – MCU complexed with accessory proteins such as MICUs, EMRE, and so on constituting CaUni activity, NCLX for NCX_mit_ activity, and Letm1 or TMBIM5 for HCX_mit_ activity – and their roles in cardiomyocyte functions have been extensively studied (Austin et al., 2022; Kwong et al., 2015; Luongo et al., 2015; Natarajan et al., 2020; Takeuchi et al., 2013; see also reviews by Boyman et al., 2013; Takeuchi & Matsuoka, 2021; Takeuchi et al., 2015; Wu et al., 2015).

Mitochondria are densely and regularly aligned in ventricular myocytes, and heterogenous localization of Ca^2+^_mit_ handling proteins and their contributions to spatial Ca^2+^ movements inside the myocytes have attracted the attention of many researchers. De La Fuente et al. (2016) reported that in mouse and rat ventricular myocytes distributions of MCU and EMRE are biased towards the mitochondria‐sarcoplasmic reticulum (SR) interface, which the SR Ca^2+^ release channel ryanodine receptor (RyR) faces (De La Fuente et al., 2016). They defined the region as a ‘Ca^2+^_mit_ uptake hotspot’ and subsequently found that it lacks Ca^2+^_mit_ extrusion activity via NCLX (De La Fuente et al., 2018). Recently we independently found that NCLX and SR Ca^2+^ pump (SERCA) were localized in close proximity to each other in mouse ventricular myocytes (Takeuchi & Matsuoka, 2022). We further demonstrated with mathematical model analysis that the experimentally recorded automaticity and Ca^2+^_SR_ dynamics of HL‐1, a cell line derived from mouse atrial myocytes (Claycomb et al., 1998), could be reproduced only when spatial and functional couplings of mitochondria and SR via CaUni‐RyR and NCX_mi__t_‐SERCA were assumed (Takeuchi & Matsuoka, 2022). These findings have filled the last gap in explaining the efficient Ca^2+^ cycling between mitochondria and SR (De La Fuente et al., 2016, 2018). A question then arose: what are the physiological impacts of the spatial and functional couplings on the functions of intact ventricular myocytes, which have no automaticity?

Cellular energetics is one of the functions most likely to be affected by the above‐mentioned couplings, because Ca^2+^ is an important factor in both ATP consuming and producing processes. In cardiomyocytes, the ATP consuming process mainly occurs via myosin ATPase, SERCA, Na^+^‐K^+^ ATPase (*I*_NaK_) and the plasma membrane Ca^2+^ pump (*I*_pCa_), whereas ATP is produced mainly by oxidative phosphorylation in mitochondria (Katz, 2010). In order to adapt to the dynamic increase in cardiac workload, or ATP demand, during intense exercise, ATP supply increases rapidly as demonstrated by the linear relationship between workload and myocardial oxygen consumption (mVO_2_) (Hata et al., 1994; Ingwall, 2002; Starling & Visscher, 1927). Feedback regulation by ATP hydrolysis products ADP and inorganic phosphate (Pi) has an important role for the adaptation, while allosteric regulation by Ca^2+^ has also been proposed as an additional mechanism underlying the adaptation. This idea is based on an epoch‐making finding that Ca^2+^ activates mitochondrial dehydrogenases producing NADH, which is an intermediate product connecting mitochondrial metabolism and oxidative phosphorylation (McCormack et al., 1990; Rutter & Denton, 1988). Following controversies regarding the contribution of the Ca^2+^‐dependent regulations to cardiac energetics (see reviews by Aon & Cortassa, 2012; Beard & Kushmerick, 2009; Glancy & Balaban, 2012; Korzeniewski, 2017; Saks et al., 2006; Takeuchi & Matsuoka, 2020), we proposed the idea that the composition of metabolic substrates is an important factor determining the extent to which Ca^2+^ contributes to mitochondrial energetics. Our simulation analysis using a detailed cardiac mitochondrial model demonstrated that, under the cytoplasmic substrate condition of malate/glutamate, which is frequently used in *in vitro* measurements of isolated mitochondria, steady state mitochondrial NADH (NADH_mit_) concentration was elevated by increasing the Ca^2+^_cyt_ concentration. On the other hand, when other physiological metabolic substrates were assumed as the *in vivo* condition, Ca^2+^_cyt_ had marginal effects on the steady state NADH_mit_ level (Saito et al., 2016), in line with experimental reports using isolated rat cardiac mitochondria (Vinnakota et al., 2011, Vinnakota, Singhal et al., 2016). However, it remains unclear how the Ca^2+^‐dependent regulatory mechanisms contribute to cardiac energetics and to overall cardiomyocyte function during dynamic workload transitions, such as exercise, when excitation‐contraction coupling changes vigorously.

In the present study, we constructed a new integrated model of human ventricular myocyte with excitation‐contraction‐energetics coupling and investigated systematically the contribution of the mitochondria‐SR Ca^2+^ interaction to cardiac energetics as well as to overall cardiomyocyte functions during dynamic workload transitions in exercise. Simulation analyses revealed that the spatial coupling of mitochondria and SR, particularly via CaUni‐RyR, is the primary determinant of Ca^2+^_mit_ concentration during exercise, and that it modulates the contribution of Ca^2+^_mit_‐dependent regulation of NADH‐producing dehydrogenases to the NADH_mit_ dynamics.

## Methods

### Structure of the integrated human ventricular cell model with excitation‐contraction‐energetics coupling

**Figure 1:**
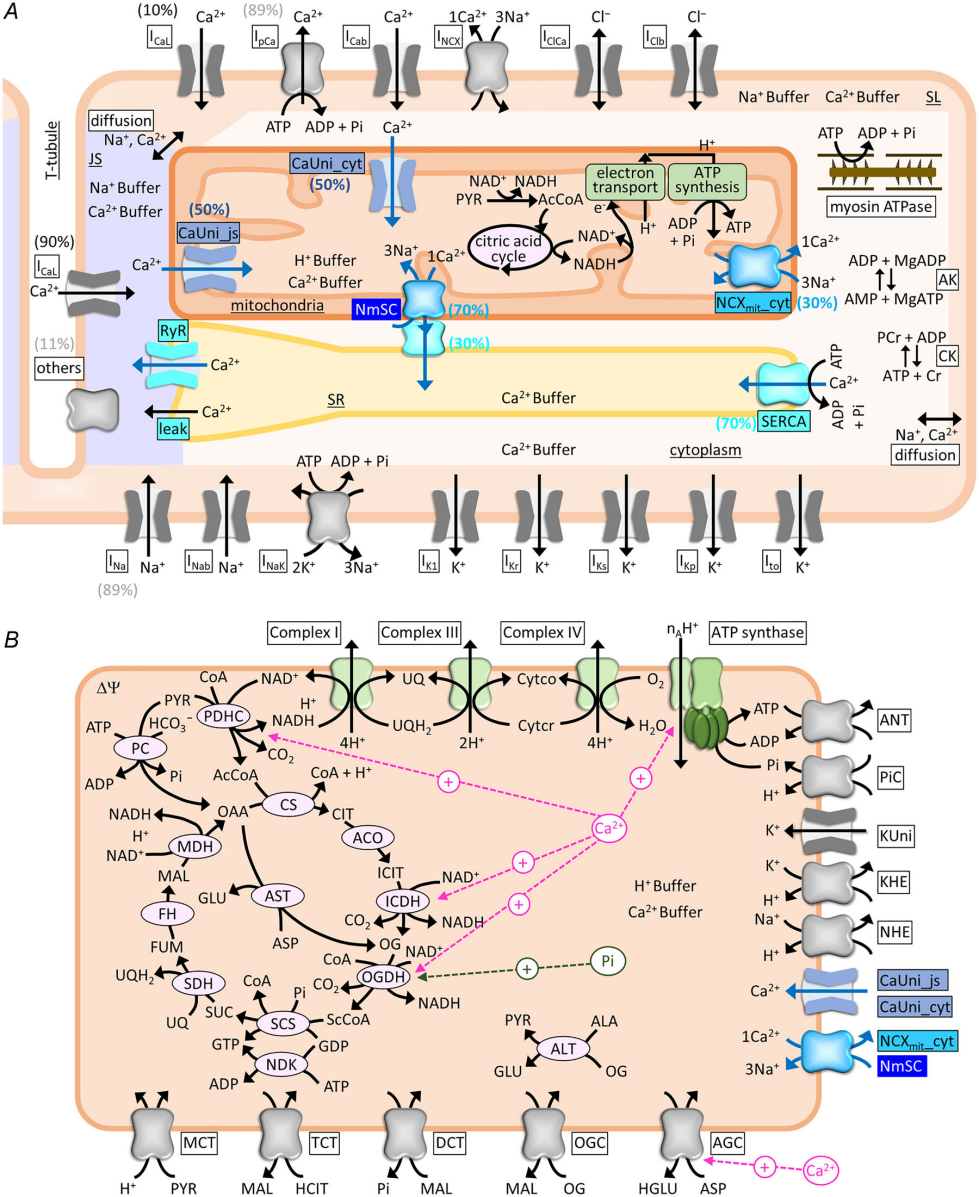
Schematic presentation of the Integrated Human Ventricular Cell Model *A*) the overall view of the model, consisting of a human ventricular myocyte model, a contraction model, and a detailed mitochondrial energetics model, with an interaction between mitochondria and SR. Distribution of *I*_CaL_ and other membrane channels/transporters (others) in JS/SL are indicated in parenthesis in black and grey, respectively. Uneven distribution of NmSC:NCX_mit__cyt and NmSC:SERCA facing cytoplasm is set as, respectively, 70%:30% and 30%:70%. Uneven distribution of CaUni_js:CaUni_cyt is set as 50%:50%. *B*) the scheme of the mitochondrial energetics part of the model which is simplified in *A*. Allosteric regulations by Ca^2+^ and Pi are indicated by magenta and green, respectively. AcCoA, acetyl CoA; ACO, aconitase; AK, adenylate kinase; ALA, alanine; ALT, alanine aminotransferase; ANT, adenine nucleotide translocase; ASP, aspartate; AST, aspartate aminotransferase; Buff, buffer; CK, creatine kinase; Cytco, oxidized form of cytochrome c; Cytcr, reduced form of cytochrome c; DCT, dicarboxylate transporter; FH, fumarate hydratase; FUM, fumarate; GLU, glutamate; HCIT, citrate; *I*_Cab_, background Ca^2+^ current; *I*_Clb_, background Cl^−^ current; *I*_ClCa_, Ca^2+^ activated Cl^−^ current; *I*_K1_, inward rectifier K^+^ current; *I*_Kp_, plateau K^+^ current; *I*_Kr_, rapidly activating K^+^ current; *I*_Na_, voltage‐dependent Na^+^ current; *I*_Nab_, background Na^+^ current; *I*_NCX_, sarcolemmal Na^+^‐Ca^2+^ exchange current; *I*_to_, transient outward K^+^ current; KUni, K^+^ uniporter; MAL, malate; NDK, nucleoside diphosphate kinase; OAA, oxaloacetate; OG, 2‐oxoglutarate; OGC, 2‐oxoglutarate/malate carrier PC, pyruvate carboxylase; PYR, pyruvate; ScCoA, succinyl CoA; SCS, succinyl CoA synthase; SDH, succinate dehydrogenase; SUC, succinate; TCT, tricarboxylate transporter; UQ, ubiquinone; UQH_2_, ubiquinol.

The Integrated Human Ventricular Cell Model was created in Visual C# (Microsoft Visual Studio 2022) and the ordinary differential equations were integrated using the Runge‐Kutta method with an adaptive time step in a range of 10^−6^ to 5.0 × 10^−3^ ms. The source code is available on GitHub at https://github.com/atakeuti/IHVCM. The model consisted of a human ventricular myocyte model (Grandi et al., 2010), a contraction model (Shim et al., 2007), an interaction between mitochondria and SR (Takeuchi & Matsuoka, 2022), and a detailed mitochondrial energetics model (Saito et al., 2016) (Fig. 1*A*). All equations are presented in the Online Supplementary Material. In order to obtain ion concentrations comparable to the original Grandi model after implementing the above components, several parameters were fine‐tuned. The amplitude factors for SERCA, RyR, SR Ca^2+^ Leak and *I*_NaK_ were set to 1.5, 1.5, 2.3 and 0.9 times of the original, respectively. The Ca^2+^ diffusion permeabilities for junctional space (JS)‐subsarcolemmal space (SL) and cytoplasm‐SL were set 1.1 times of the original. One of the cytoplasmic Ca^2+^ buffer systems in the Grandi model, troponin C (TnClow), was replaced with that used in the contraction model (Shim et al., 2007).

### Contraction model

**Figure 2:**
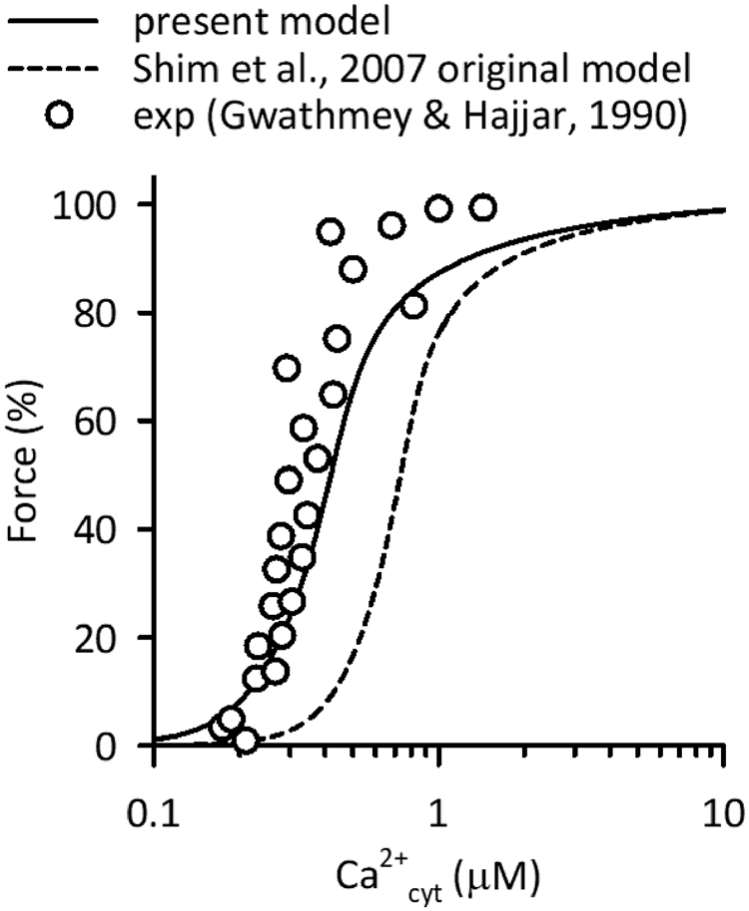
Dependence of force in the contraction model on Ca^2+^_cyt_ The contraction model (Shim et al., 2007) with the present (continuous line) and original (dashed line) parameters applied, with *F*_ext_ of 9.15 and 8.45, respectively, to obtain hsmL of 1.10 μm, under the condition of isometric contraction with various Ca^2+^_cyt_ concentrations. The steady state force was obtained and was normalized to the maximal value. Experimental data using human cardiac muscle (exp; open circles) were from Gwathmey and Hajjar (1990).

We adapted the contraction model by Shim et al. (2007) with minor modifications, to the Integrated Human Ventricular Cell Model. In particular, the Ca^2+^ binding rate of troponin, *Y*_1_ (ms^−1^), was modified to better reproduce the Ca^2+^_cyt_ concentration‐force relationship measured using human myocardium (Gwathmey & Hajjar, 1990) (Fig. 2).

$$Y_{1}\;=\;12.8\times {\left({\left[Ca^{2+}\right]}_{\mathrm{cyt}}\right)}^{0.81}.$$

The magnitude factor *B*_eff_, model fit parameter *L*_0_, and total troponin concentration were fine‐tuned to obtain contraction comparable to the original model after this modification. See details in the Online Supplementary Material Table S7. The increase in half‐sarcomere length (hsmL) is achieved by increasing the external load (*F*_ext_) applied to the model cell.

### Mitochondria‐SR interaction (MSI) model

Based on super‐resolution imaging studies on the uneven distributions of Ca^2+^_mit_ and Ca^2+^_SR_ handling proteins in ventricular myocytes (De La Fuente et al., 2016; Takeuchi & Matsuoka, 2022), the mitochondria‐SR interaction (MSI) component developed in the HL‐1 cell model (Takeuchi & Matsuoka, 2022) was implemented in the Integrated Human Ventricular Cell Model. In short, we assumed the NCX_mit_‐SERCA complex (NmSC) and the CaUni facing JS (CaUni_js) (see Fig. 1*A*). In the control MSI model, the amplitude factors for NmSC and CaUni_js were set to 70% and 50% of total NCX_mit_ and total CaUni, respectively. The remaining 30% and 50% of NCX_mit_ and CaUni, defined as NCX_mit__cyt and CaUni_cyt, respectively, were set to face the cytoplasm. In addition, 30% of SERCA was replaced with NmSC and the remaining 70% was set to face the cytoplasm. Functional coupling of NCX_mit_ and SERCA was expressed by assuming that Ca^2+^, extruded from the mitochondria via the NmSC, directly enters the SR. When MSI was not considered (non‐MSI) in the model, the fractions of CaUni_js and NmSC were set to 0%, and those of CaUni_cyt and NCX_mit__cyt to 100% (Fig. 10). The amplitude factor for SERCA in the non‐MSI model was set to 1.43 times, i.e. 100%/70%, that of the MSI model.

The CaUni model (Takeuchi & Matsuoka, 2022) was updated by implementing the term Ca^2+^_mit_‐dependent regulation, Ca_mit__reg, according to the experimental report (Vais et al., 2016):

$$\begin{array}{ccc} Ca_{\mathrm{mit}\_\mathrm{reg}}\; & = & \;0.9\;-\left(\frac{{\left[Ca^{2+}\right]}_{\mathrm{mit}}}{{\left[Ca^{2+}\right]}_{\mathrm{mit}}+K_{\mathrm{inh}}}-0.3\right)\\ & & \times \left(\frac{{K_{\mathrm{rec}}}^{n}}{{{\left[Ca^{2+}\right]}_{\mathrm{mit}}}^{n}+{K_{\mathrm{rec}}}^{n}}-0.3\right), \end{array}$$

where the apparent inhibition constant of Ca^2+^_mit_, *K*_inh_ = 5.0 × 10^−5^ mm, the apparent recovery constant of Ca^2+^_mit_, *K*_rec_ = 8.0 × 10^−4^ mm, and the Hill coefficient *n* = 2.

The NCX_mit_ model (Takeuchi & Matsuoka, 2022) was updated by increasing the Ca^2+^_mit_ affinity; i.e. reducing the apparent binding constant of Ca^2+^_mit_, *K*_d_Ca_mit_, from 0.0209 to 0.0025 mm, based on our electrophysiological measurements using mouse cardiac mitoplasts (Islam et al., 2020). Accordingly, the apparent binding constant of mitochondrial Na^+^, *K*_d_Na_mit_, was reduced from 38.0000 to 18.7137 mm to satisfy the constraint of the equilibrium potential of NCX_mit_, *E*_Na/Ca_ (Kim & Matsuoka, 2008):

$$E_{\mathrm{Na}/\mathrm{Ca}}\;=\;3E_{\mathrm{Na}}-\;2E_{\mathrm{Ca}},$$

$$\frac{{\left(K_{d}{\mathrm{Na}}_{\mathrm{cyt}}\right)}^{3}\times K_{d}{\mathrm{Ca}}_{\mathrm{mit}}}{{\left(K_{d}{\mathrm{Na}}_{\mathrm{mit}}\right)}^{3}\times K_{d}{\mathrm{Ca}}_{\mathrm{cyt}}}\;=\;1.0,$$

where *E*_Na_, *E*_Ca_, *K*_d_Na_cyt_ and *K*_d_Ca_cyt_ are the equilibrium potential of Na^+^, equilibrium potential of Ca^2+^, apparent binding constant of cytosolic Na^+^ (Na^+^_cyt_), and apparent binding constant of Ca^2+^_cyt_, respectively.

The amplitude factors for CaUni and NCX_mit_ were set to obtain comparable time courses of Ca^2+^_mit_ increase upon stimulus frequency change in rat trabeculae and guinea‐pig ventricular myocytes (Brandes & Bers, 2002; Jo et al., 2006).

### Cardiac mitochondrial model

Our isolated mitochondrial model (Saito et al., 2016) was adapted for the energetics part of the Integrated Human Ventricular Cell Model. The model consisted of oxidative phosphorylation, the citric acid cycle, the pyruvate pathway, and substrate/ion transporters. Ca^2+^_mit_‐dependent regulation of three dehydrogenases (pyruvate dehydrogenase (PDCH), isocitrate dehydrogenase (ICDH), and oxoglutarate dehydrogenase (OGDH)) and F_1_F_o_‐ATPase (ATP synthase), and Ca^2+^_cyt_‐dependent activation of aspartate/glutamate carrier (AGC) were implemented (Rutter & Denton, 1988; McCormack et al., 1990; Territo et al., 2000; Wescott et al., 2019; Fig. 1*B*). In the original Saito et al. (2016) model, we assumed that mitochondrial Pi (Pi_mit_) allosterically activates Complex III of electron transport chain, based on the experimental report by Bose et al. (2003). Although this regulation was shown to contribute to metabolic stability in various models including ours (Beard, 2005; Cortassa et al., 2006; Saito et al., 2016; Tran et al., 2015), it was subsequently denied experimentally (Bazil et al., 2016; Vinnakota, Bazil et al., 2016). Therefore, we decided to remove the Pi_mit_‐dependent activation of Complex III in the updated model.

In order to obtain resting mVO_2_ comparable to that reported for the human hearts (e.g. 3.57 mM/min, calculated from ∼8 ml/100 g/min (Strauer, 1979)), the amplitude factors for mitochondrial transporters/channels/enzymes were doubled, except for the monocarboxylate transporter (MCT), which was multiplied by four to prevent depletion of mitochondrial pyruvate. The mVO_2_ was 1.88 mM/min when stimulated with a cycle length of 1 s, on the same order of magnitude as the reported value, but slightly lower. This is possibly due to the difference between the estimates obtained by single cell model and by *in vivo* measurements.

Since the components for Ca^2+^ fluxes, CaUni and NCX_mit_, were replaced with those in the MSI model as described above, the Ca^2+^_mit_‐ and Ca^2+^_cyt_‐dependent activities of ATP synthase and AGC, respectively, as well as the amplitude factors for Na^+^‐H^+^ exchanger (NHE) and H^+^ leak, were fine‐tuned.

The updated isolated mitochondrial model well reproduced the *in vitro* experiments on Ca^2+^_cyt_‐dependent changes of NADH_mit_ and mVO_2_ using isolated cardiac mitochondria (Appendix Fig. A*1A*) (Territo et al., 2000). In addition, qualitatively comparable results were obtained for cytoplasmic Pi (Pi_cyt_)‐dependency observed using isolated cardiac mitochondria (Bose et al., 2003) (Appendix Fig. A*1B*), albeit with deviations due to the removal of Pi_mit_‐dependent activation of Complex III.

### Basic characteristics of the integrated human ventricular cell model

The initial standard condition of the Integrated Human Ventricular Cell Model, which corresponds to the steady state condition for isotonic contraction stimulated with a cycle length of 1 s, is listed in the Online Supplementary Material Table S14.

Configurations of action potential, major ionic currents, Ca^2+^ fluxes and Ca^2+^ concentrations in each compartment are shown in the Appendix Figure A2. These are similar to the original Grandi model except for mitochondrial Ca^2+^ dynamics, which was not implemented in the original model.

**Figure 3:**
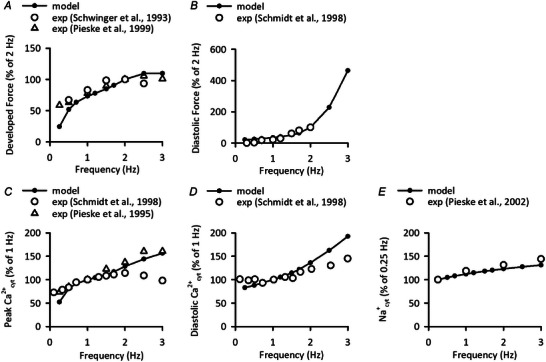
Dependences of force and ion concentrations in the Integrated Human Ventricular Cell Model on stimulus frequency The Integrated Human Ventricular Cell Model was applied with *F*_ext_ of 4.25 to obtain 1.050 μm hsmL under the condition of isometric contraction, and then stimulated at various frequencies for 20 min. The steady state values were obtained and normalized to the given stimulus frequency, as indicated in the graph (filled circles). *A*) developed force. Experimental data using human cardiac muscle were from Schwinger et al. (1993) (open circles) and from Pieske et al. (1999) (open triangles). *B*) diastolic force. Experimental data using human cardiac muscle were from Schmidt et al. (1998) (open circles). *C*) peak Ca^2+^_cyt_ concentration. Experimental data using human cardiac muscle were from Schmidt et al. (1998) (open circles) and from Pieske et al. (1995) (open triangles). *D*) diastolic Ca^2+^_cyt_ concentration. Experimental data using human cardiac muscle were from Schmidt et al. (1998) (open circles). *E*) Na^+^_cyt_ concentration. Experimental data using human cardiac muscle were from Pieske et al. (2002) (open circles).

The frequency dependence of the model was examined in Fig. 3. The model was stimulated for 20 min at different frequencies under condition of isometric contraction with *F*_ext_ of 4.25 to obtain 1.050 μm hsmL in similar manner to experiments of human ventricular trabeculae (Pieske et al., 1995, 1999, 2002; Schwinger et al., 1993; Schmidt et al., 1998). The model well reproduced the experimental data on frequency‐dependent changes in force and cytoplasmic ion concentrations (Fig. 3).

In the HL‐1 cardiomyocyte, blocking NCX_mit_ decreased Ca^2+^_SR_ concentration and decelerated Ca^2+^_SR_ reuptake in the caffeine application protocol (Takeuchi et al., 2013; Takeuchi & Matsuoka, 2022). Incorporation of the MSI component in the HL‐1 model was necessary to reproduce these experimental results (Takeuchi & Matsuoka, 2022). In the Integrated Human Ventricular Cell Model considering MSI, NCX_mit_ reduction decreased Ca^2+^_SR_ concentration and decelerated Ca^2+^_SR_ reuptake after caffeine application, whereas the reduction hardly affected them in the non‐MSI model (Appendix Fig. A3), being comparable to those obtained using the HL‐1 cell model (Takeuchi & Matsuoka, 2022). Note that with the MSI model, Ca^2+^_SR_ concentration slightly increased when the amplitude factor for NCX_mit_ was reduced from 1.0 to 0.5 and 0.3 (filled circles in Fig. A3*E*). Under these conditions, the Ca^2+^_mit_ concentration increased to several micromolar, and the residual NmSC was able to supply more Ca^2+^ to the SR.

### Simulation protocols for dynamic workload change

To simulate dynamic workload changes in exercise, the following three interventions were applied to the model in different combinations; (1) shortening of stimulation cycle length mimicking heart rate increase, (2) increase in *F*_ext_ mimicking ventricular end‐diastolic volume increase, namely preload increase, and (3) β‐adrenergic receptor stimulation mimicking contractility increase, according to the literature on exercise in humans (Kang et al., 2017; Kurata et al., 2019; Lind & McNicol, 1967; Veldkamp et al., 2001; see also textbook by Herring & Paterson, 2018).

As a starting resting condition, the model was stimulated with a cycle length of 1 s, i.e. a heart rate of 60 min^−1^, with isotonic contraction. Under the resting condition, diastolic hsmL was 0.950 μm without applying *F*_ext_. The standard exercise condition was set as cycle length shortening to 0.33 s, i.e. heart rate increase to 180 min^−1^, *F*_ext_ increase to 5.00, i.e. diastolic hsmL elongation to 1.050 μm, and β‐adrenergic receptor stimulation with the increased amplitude factors for L‐type Ca^2+^ current (*I*_CaL_), SERCA, *I*_NaK_, slowly activating K^+^ current (*I*_Ks_) as 1.30, 1.30, 1.20 and 12 times, respectively, plus the negative voltage shift of *I*_Ks_ activation gate as −5.0 mV. This setting of β‐adrenergic receptor stimulation was adapted from Kurata et al. (2019), with minor modifications especially for *I*_Ks_. Since the contribution of *I*_Ks_ to repolarization was set extremely low in the original Grandi model, it was necessary to increase the amplitude factor by 12 times and to shift the voltage by −5.0 mV, to obtain a comparable action potential configuration. The interventions were applied gradually with a time constant of 40 s, based on the literature on the heart rate change during exercise in humans (Lind & McNicol, 1967; Lemire et al., 2021).

In the simulations of Fig. 9, target cycle length, target *F*_ext_ value, and extent of β‐adrenergic receptor stimulation were varied to obtain various extent of exercise. The *F*_ext_ of 0.00–10.00 correspond to diastolic hsmLs of 0.950–1.100 μm when stimulated with a cycle length of 1 s. Configurations of hsmL shortenings with various *F*_ext_ values are presented in the Appendix Fig. A4. The extent of β‐adrenergic receptor stimulation was changed using the following terms:

For *I*_CaL_:

$$AI_{\mathrm{CaL}}=\;1+\alpha \times 0.3,$$

$$P_{\mathrm{Ca}}\;=\;AI_{\mathrm{CaL}}\times P_{\mathrm{Ca},0,}$$

$$P_{\mathrm{Na}}\;=\;AI_{\mathrm{CaL}}\times P_{\mathrm{Na},0},$$

$$P_{K}\;=\;AI_{\mathrm{CaL}}\times P_{K,0},$$

where *P*_Ca_, *P*_Na_ and *P*_K_ are permeability factors for Ca^2+^, Na^+^ and K^+^, respectively, and *P*_Ca,0_, *P*_Na,0_ and *P*_K,0_ are permeability factors in the absence of β‐adrenergic receptor stimulation for Ca^2+^, Na^+^ and K^+^, respectively.

For SERCA:

ASERCA=1+α×0.3,

$$\;V_{\max\_\mathrm{SERCA}}=\;A\mathrm{SERCA}\times V_{\max\_\mathrm{SERCA},0},$$

where *V*_max_SERCA_ is maximum velocity and *V*_max_SERCA,0_ is maximum velocity in the absence of β‐adrenergic receptor stimulation.

For *I*_NaK_:

$$AI_{\mathrm{NaK}}=\;1+\alpha \times 0.2,$$

$$\;V_{\max\_\mathrm{INaK}}=\;AI_{\mathrm{NaK}}\times V_{\max\_\mathrm{INaK},0},$$

where *V*_max_INaK_ is maximum velocity and *V*_max_INaK,0_ is maximum velocity in the absence of β‐adrenergic receptor stimulation.

For *I*_Ks_:

$$AI_{\mathrm{Ks}}=\;1+\alpha \times 11,$$

$$\;G_{\mathrm{Ks}}=\;AI_{\mathrm{Ks}}\times G_{\mathrm{Ks},\;0},$$

Vshift=α×5.0,

$$xKs_{\mathrm{ss}}=\frac{1}{1+\exp\left(-\frac{Vm+3.8+V\mathrm{shift}}{14.25}\right)},$$

$$\tau_{\mathrm{xKs}}=\frac{990.1}{1+\exp\left(-\frac{Vm+2.436+V\mathrm{shift}}{14.12}\right)},$$

where *G*_Ks_, *G*_Ks,0_, *V*shift, *x*Ks_ss_ and τ_xKs_ are conductance, conductance in the absence of β‐adrenergic receptor stimulation, voltage shift factor in the presence of β‐adrenergic receptor stimulation, steady‐state value of gate and time constant of gate, respectively.

For the β‐adrenergic receptor stimulation, α value is varied from 0 to 3.0. In the standard exercise condition, α = 1.0.

In order to analyse the effects of Ca^2+^_mit_‐dependent activations of three dehydrogenases, pyruvate dehydrogenase (PDHC), isocitrate dehydrogenase (ICDH) and oxoglutarate dehydrogenase (OGDH), simulations of ‘silenced Ca^2+^_mit_‐dependent regulation’ were performed. In these simulations, the following allosteric regulation terms were set to refer to the average Ca^2+^_mit_ concentration per stimulation cycle prior to exercise, expressed as FCa_mit_.

For PDHC:

$$\;f_{\mathrm{PDHa}}={\left(1+u_{2}\times \left(1+\frac{u_{1}\times {K_{\mathrm{Ca}}}^{n\mathrm{Ca}}}{{K_{\mathrm{Ca}}}^{n\mathrm{Ca}}+\mathrm{FC}{a_{\mathrm{mit}}}^{n\mathrm{Ca}}}\right)\right)}^{-1}\;,$$

where *f*_PDHa_, *K*_Ca_ and *n*Ca are activation factor by Ca^2+^_mit_, binding constant of Ca^2+^_mit_, and Hill coefficient, respectively. *u*_1_ and *u*_2_ are model fitted factors.

For ICDH:

$$\;{\mathrm{Ca}}_{\mathrm{Act}1\_1}=\;1+\frac{\alpha_{\mathrm{ADP}}\times \beta_{\mathrm{Ca}1}\times \mathrm{FC}a_{\mathrm{mit}}}{\alpha_{\mathrm{Ca}1}\times \beta_{\mathrm{ADP}}\times K_{\mathrm{Ca}1}\times \left(1+{\left(\frac{{\left[{\mathrm{Mg}}^{2+}\right]}_{\mathrm{mit}}}{K_{\mathrm{iMg}2}}\right)}^{2}\right)},$$

$$\;Ca_{\mathrm{Act}1\_2}=\;1+\frac{\alpha_{\mathrm{ADP}}\times \mathrm{FC}a_{\mathrm{mit}}}{\alpha_{\mathrm{Ca}1}\times K_{\mathrm{Ca}1}\times \left(1+{\left(\frac{{\left[Mg^{2+}\right]}_{\mathrm{mit}}}{K_{\mathrm{iMg}2}}\right)}^{2}\right),}$$

$$\;Ca_{\mathrm{Act}1\_3}=\;1+\frac{\mathrm{FC}a_{\mathrm{mit}}}{K_{\mathrm{Ca}1}\times \left(1+{\left(\frac{{\left[Mg^{2+}\right]}_{\mathrm{mit}}}{K_{\mathrm{iMg}2}}\right)}^{2}\right)},$$

$$\;Ca_{\mathrm{Act}2\_1}=\;1+\frac{\alpha_{\mathrm{ATP}}\times \beta_{\mathrm{Ca}2}\times \mathrm{FC}a_{\mathrm{mit}}}{\alpha_{\mathrm{Ca}2}\times \beta_{\mathrm{ATP}}\times K_{\mathrm{Ca}2}\times \left(1+{\left(\frac{{\left[Mg^{2+}\right]}_{\mathrm{mit}}}{K_{\mathrm{iMg}2}}\right)}^{2}\right),}$$

$$\;Ca_{\mathrm{Act}2\_2}=\;1+\frac{\alpha_{\mathrm{ATP}}\times \mathrm{FC}a_{\mathrm{mit}}}{\alpha_{\mathrm{Ca}2}\times K_{\mathrm{Ca}2}\times \left(1+{\left(\frac{{\left[Mg^{2+}\right]}_{\mathrm{mit}}}{K_{\mathrm{iMg}2}}\right)}^{2}\right)},$$

$$\;Ca_{\mathrm{Act}2\_3}=\;1+\frac{\mathrm{FC}a_{\mathrm{mit}}}{K_{\mathrm{Ca}2}\times \left(1+{\left(\frac{{\left[Mg^{2+}\right]}_{\mathrm{mit}}}{K_{\mathrm{iMg}2}}\right)}^{2}\right)},$$

where Ca_ACT_, *K*_Ca_ and *K*_iMg2_ are activation factors by Ca^2+^_mit_, binding constants of Ca^2+^_mit_, and binding constant for inhibition by Mg^2+^_mit_, respectively. α and β are model fitted factors.

For OGDH:

$$\;{\mathrm{Ca}}_{\mathrm{Act}1}=\;1+{\left(\frac{\mathrm{FC}a_{\mathrm{mit}}}{K_{\mathrm{Ca}}}\right)}^{n\mathrm{Ca}},$$

$$\;{\mathrm{Ca}}_{\mathrm{Act}2}=\;1+{\left(\frac{\mathrm{FC}a_{\mathrm{mit}}}{\alpha_{\mathrm{Ca}}\times K_{\mathrm{Ca}}}\right)}^{n\mathrm{Ca}},$$

$$\;{\mathrm{Ca}}_{\mathrm{Act}3}=\;1+\beta_{\mathrm{Ca}}\times {\left(\frac{\mathrm{FC}a_{\mathrm{mit}}}{\alpha_{\mathrm{Ca}}\times K_{\mathrm{Ca}}}\right)}^{n\mathrm{Ca}},$$

where Ca_ACT_, *K*_Ca_ and *n*Ca are activation factors by Ca^2+^_mit_, binding constant of Ca^2+^_mit_, and Hill coefficient, respectively. α and β are model fitted factors.

For the control simulations with allosteric Ca^2+^_mit_‐dependent regulations, Ca^2+^_mit_ was used instead of FCa_mit_ (Saito et al., 2016). Full equations are summarized in the Online Supplementary Material Table S9.

## Results

### Characteristics of the integrated human ventricular cell model in the isotonic contraction

**Figure 4:**
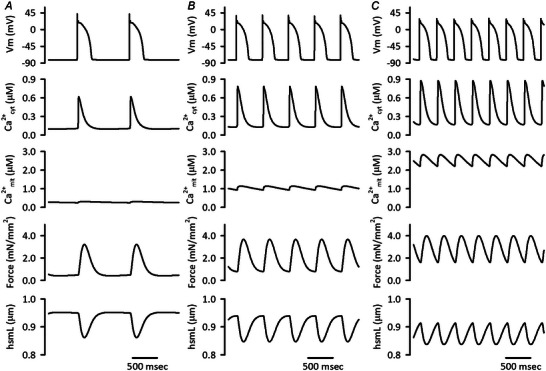
Configurations of action potential, Ca^2+^_cyt_, Ca^2+^_mit_, force and hsmL shortenings stimulated with cycle lengths of 1–0.33 s in the Integrated Human Ventricular Cell Model The steady state variables stimulated with cycle lengths of 1 s (*A*), 0.5 s (*B*) and 0.33 s (*C*) were plotted. The initial condition was obtained without applying *F*_ext_, i.e. 0.950 μm diastolic hsmL, under the condition of isotonic contraction stimulated with cycle length of 1 s for 30 min, as shown in the Online Supplementary Material Table S14.

To simulate cardiac workload transitions during exercise, the model cell was stimulated with various cycle lengths under the condition of isotonic contraction as shown later. The steady state action potential configurations, Ca^2+^_cyt_ transients, Ca^2+^_mit_, active force generations, and hsmL shortenings stimulated with cycle lengths of 1, 0.5 and 0.33 s are shown in the Fig. 4. Shortening the cycle length from 1 to 0.5 s increased amplitude of Ca^2+^_cyt_ transients, resulting in larger developed force and hsmL shortening, though the extents were small. Further cycle length shortening to 0.33 s augmented diastolic Ca^2+^_cyt_ and force levels, resulting in shortened diastolic hsmL. Accordingly, the developed force became smaller with cycle lengths of 0.33 s, which was more noticeable than that seen in the isometric contraction (see Fig. 3). The beat‐to‐beat oscillation of Ca^2+^_mit_ was small, i.e. the amplitude was 60.9 nm, when stimulated with cycle length of 1 s. The shorter cycle length induced accumulation of Ca^2+^_mit_, which was caused by fast and slow Ca^2+^ fluxes of CaUni and NCX_mit_, respectively. These characteristics are comparable to experimental data for rat and rabbit ventricular myocytes, as well as human atrial myocytes (Lu et al., 2013; Mason et al., 2020; Wust et al., 2017).

Energetics‐related parameters are summarized in the Table 1. In the Integrated Human Ventricular Cell Model, ATP was consumed by contraction (62.99%), SERCA (28.59%), *I*_NaK_ (7.75%) and *I*_pCa_ (0.67%), when stimulated with cycle length of 1 s. These values are close to experimental estimates made in guinea‐pig ventricular muscle (Schramm et al., 1994): contraction (76%), SERCA (15%) and *I*_NaK_ (9%). The phosphocreatine (PCr)/ATP concentration ratio was 2.33, which is comparable to experimental values 1.9–2.1 in the human hearts measured by ^31^P cardiac magnetic resonance spectroscopy (Phan et al., 2009; Smith et al., 2006).

**Table 1:** Energetics‐related parameters of the Integrated Human Ventricular Cell Model with (MSI) or without (non‐MSI) mitochondria‐SR interaction.

	MSI model			non‐MSI model
	1 Hz	2 Hz	3 Hz	1 Hz
ATPuse by contraction (mM/min) (% of total ATPuse)	6.076 (62.99)	10.935 (59.27)	15.210 (57.49)	3.886 (60.86)
ATPuse by SR Ca^2+^ uptake (mM/min) (% of total ATPuse)	2.758 (28.59)	6.331 (34.32)	9.730 (36.78)	1.667 (26.10)
ATPuse by *I*_NaK_ (mM/min) (% of total ATPuse)	0.748 (7.75)	1.079 (5.85)	1.374 (5.20)	0.766 (12.00)
ATPuse by *I*_pCa_ (mM/min) (% of total ATPuse)	0.064 (0.67)	0.104 (0.56)	0.141 (0.53)	0.067 (1.05)
total ATP_cyt_ (mM)	6.682	6.677	6.671	6.683
total ADP_cyt_ (mM)	0.018	0.022	0.028	0.017
PCr_cyt_ (mM)	15.556	14.257	12.857	16.031
Pi_cyt_ (mM)	0.366	0.865	1.630	0.239
PCr/ATP	2.33	2.14	1.93	2.40
mVO_2_ (mM/min)	1.882	3.431	4.880	1.273
Δψ (mV)	−180.490	−176.221	−172.700	−183.507
NADH_mit_ (mM)	1.188	1.232	1.273	1.416

The cycle length shortenings from 1 to 0.5 and 0.33 s increased mVO_2_ by 1.8 and 2.6 times, respectively. While cytoplasmic ATP (ATP_cyt_) concentration was hardly affected, cytoplasmic ADP (ADP_cyt_) and Pi (Pi_cyt_) concentrations increased and cytoplasmic PCr (PCr_cyt_) concentration decreased. Steady state NADH_mit_ level was maintained within a 10% change, comparable to an experimental report using rabbit hearts (Heineman & Balaban, 1993).

**Figure 5:**
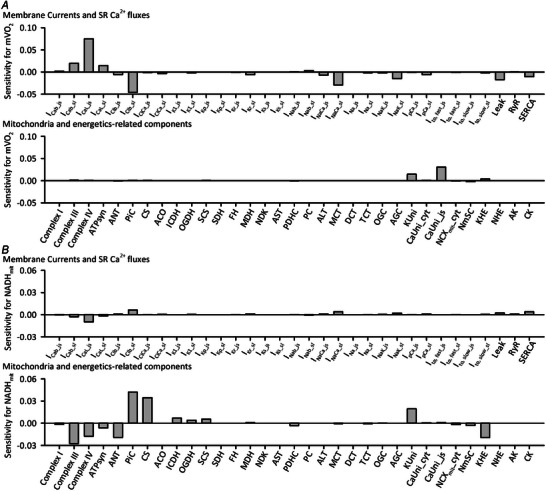
Sensitivity analyses of the Integrated Human Ventricular Cell Model The model was stimulated with a cycle length of 1 s with ± 5% change of the amplitude factor for each component for 20 min. The initial condition was the same as in Fig. 4. Sensitivity was calculated as the relative change of mVO_2_ (*A*) and NADH_mit_ (*B*) as follows: $\mathrm{Sensitivity}\;=\;\frac{\left(X_{+5\%}\;-\;X_{-5\%}\right)}{X_{\mathrm{original}}}.$

To study contribution of individual component to mVO_2_ and NADH_mit_, sensitivity analyses were performed. As expected from the linear relationship between mVO_2_ and workload, the Ca^2+^_cyt_ handling components had great influence on mVO_2_ (Fig. 5*A*). CaUni_js had a relatively greater influence on mVO_2_, because it highly affected Ca^2+^_mit_ and Ca^2+^_SR_ concentrations, as will be demonstrated later. Regarding NADH_mit_, the NADH‐producing enzymes ICDH and OGDH had some influence on NADH_mit_ levels (Fig. 5*B*). The factors involved in mitochondrial H^+^ dynamics, such as electron transport chain, ATP synthase, phosphate carrier (PiC), K^+^‐H^+^‐exchanger (KHE) and citrate synthase (CS) were influential. Complex I was less influential, possibly because it is not the rate‐limiting factor of electron transport chain (Dalmonte et al., 2009).

### Validation of stimulus frequency dependences of NADH_mit_, Ca^2+^_mit_, force and hsmL

**Figure 6:**
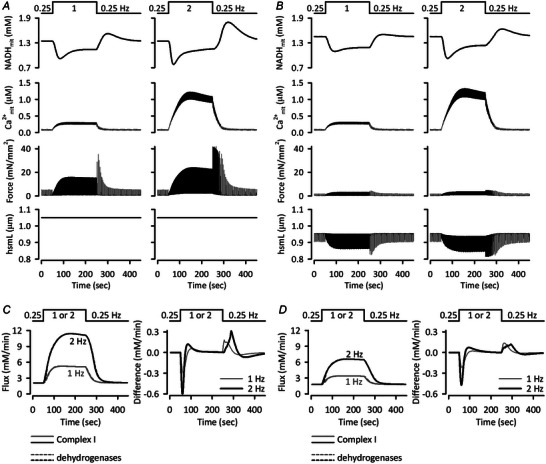
Responses of NADH_mit_, Ca^2+^_mit_, force and hsmL in the Integrated Human Ventricular Cell Model to an abrupt increase of the stimulus frequency *A*) the Integrated Human Ventricular Cell Model was applied with *F*_ext_ of 4.25, i.e. 1.050 μm diastolic hsmL, under the condition of isometric contraction, and was then stimulated with cycle length of 4 s, i.e. at 0.25 Hz, for 20 min to obtain steady state. Abrupt increases in the stimulus frequency to 1 Hz (cycle length 1 s, left panels) and 2 Hz (cycle length 0.5 s, right panels) were applied at 50 s for 200 s. *B*) the model with the initial condition as in Fig. 4, isotonic contraction with *F*_ext_ of 0.00, was stimulated at 0.25 Hz for 20 min to obtain steady state. Simulation protocol was the same as in *A*. *C* and *D*) NADH‐consuming flux of Complex I (continuous lines) and NADH‐producing fluxes of all dehydrogenases, PDHC, ICDH, OGDH and MDH (dashed lines) (left panels), and the difference, NADH‐producing flux minus NADH‐consuming fluxes (right panels) in the simulations shown in *A* and *B*, respectively.

To further validate the Integrated Human Ventricular Cell Model, we next focused on stimulus frequency‐dependent, dynamic change of NADH_mit_, which was experimentally observed in rat ventricular trabeculae (Brandes & Bers, 1997, 2002) and in guinea‐pig ventricular myocytes (Jo et al., 2006). The model was first stimulated at 0.25 Hz under isometric (Fig. 6*A*) or isotonic (Fig. 6*B*) conditions, then stimulus frequency was abruptly increased to 1 or 2 Hz for 200 s. The model well reproduced the experimentally observed biphasic NADH_mit_ changes, i.e. decrease followed by increase, and Ca^2+^_mit_ accumulation (Brandes & Bers, 1997, 2002; Jo et al., 2006). The NADH_mit_ levels are determined by the balance between consumption via Complex I of the electron transport chain and production via four dehydrogenases, PDHC, ICDH, OGDH and malate dehydrogenase (MDH). As the stimulus frequency increased, both NADH consumption and production fluxes increased (left panels of Fig. 6*C, D*), with the consumption fluxes increasing at earlier timing as evidenced by the flux difference plots (right panels of Fig. 6*C, D*), causing the biphasic NADH_mit_ changes (Fig. 6*A, B*). The model also demonstrated greater shortenings of hsmL upon stimulus frequency increase with isotonic contraction (Fig. 6*B*), also comparable to experimental data (Jo et al., 2006). The generation of larger developed forces at higher stimulus frequencies (Fig. 6*A, B*) is a characteristic of human ventricular trabeculae (see also Fig. 3) that is distinct from rat ventricular trabeculae (Brandes & Bers, 1997, 2002; Maier et al., 2000).

Taken together, we conclude that the Integrated Human Ventricular Cell Model well reproduces the characteristics of excitation‐contraction‐energetics coupling of human ventricular myocytes.

### Simulation of exercise‐induced responses of cardiac energetics

Changes in cardiac energetics during dynamic workload transitions and the underlying mechanisms were explored using this model. During exercise, cardiac workload dramatically increases due to increased heart rate, preload and contractility. The *in vivo* exercise condition was established based on the literature, as described in the Methods section.

**Figure 7:**
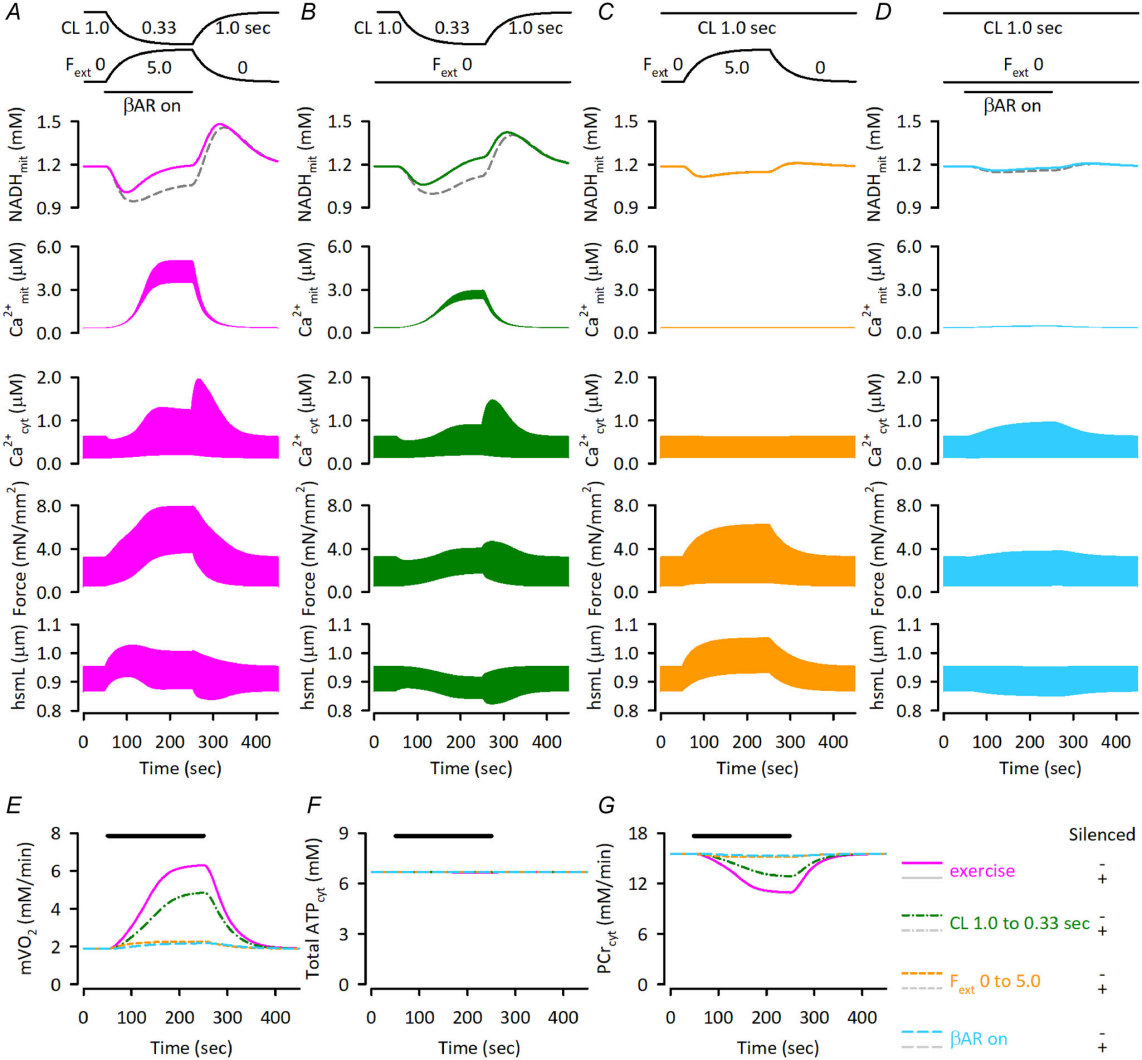
Responses of the Integrated Human Ventricular Cell Model to dynamic changes of workload by various interventions The dynamic changes of workload were applied gradually at 50 s for 200 s with a time constant of 40 s. *A*) responses of NADH_mit_, Ca^2+^_mit_, force and hsmL without (control; magenta continuous lines) or with (dashed grey lines) silenced Ca^2+^_mit_‐dependent regulation to exercise protocol, composed of cycle length (CL) shortening to 0.33 s, *F*_ext_ increase to 5.0, and application of α = 1.0 for β‐adrenergic stimulation. *B*) the responses when only cycle length was shortened to 0.33 s. *C*) the responses when only *F*_ext_ was increased to 5.0. *D*) the responses when only β‐adrenergic stimulation was applied, α = 1.0. The dashed grey lines are with silenced Ca^2+^_mit_‐dependent regulation (*B–D*). *E–G)* responses of mVO_2_ (*E*), total ATP_cyt_ concentration (*F*), and PCr_cyt_ concentration (*G*) to dynamic workload changes during exercise (magenta continuous lines), cycle length shortening to 0.33 s (green dotted‐dashed lines), *F*_ext_ increase to 5.0 (orange dashed lines), and application of α = 1.0 for β‐adrenergic stimulation (sky‐blue long dashed lines). Simulation results with silenced Ca^2+^_mit_‐dependent regulation are shown as grey lines, though they overlap with those with the control regulations. The initial condition of the model was the same as in Fig. 4.

**Figure 8:**
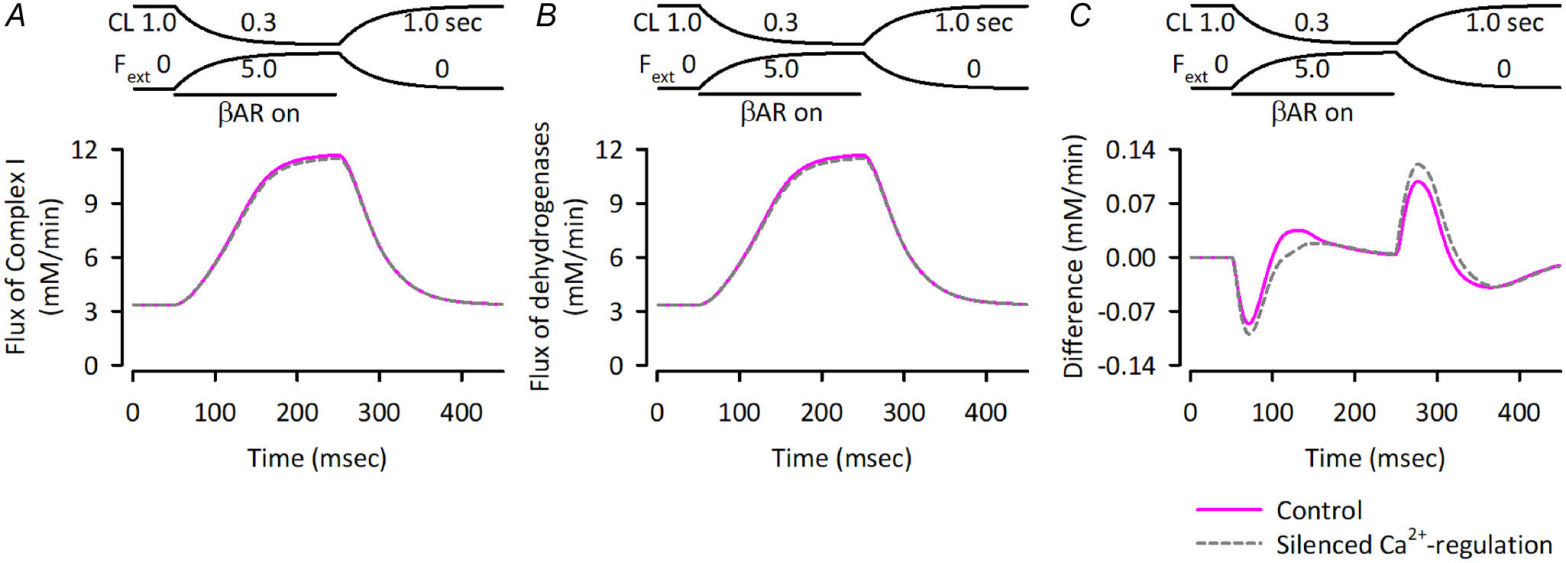
Responses of the NADH‐consuming and producing fluxes in the Integrated Human Ventricular Cell Model to exercise Responses of NADH‐consuming flux of Complex I (*A*), NADH‐producing fluxes of all dehydrogenases, PDHC, ICDH, OGDH and MDH (*B*), and the difference (*C*) without (control; magenta continuous lines) or with (dashed grey lines) silenced Ca^2+^_mit_‐dependent regulation. The initial condition and the exercise protocol were the same as in Fig. 7*A*.

Upon exercise application, NADH_mit_ initially decreased to 1.01 mm and then increased to 1.19 mm, similar to the level before the exercise onset. Ca^2+^_mit_, Ca^2+^_cyt_ and forces at diastole, systole, as well as developed, all increased during exercise (Fig. 7*A*). Among them, the Ca^2+^_mit_ increase was drastic; i.e. the diastolic Ca^2+^_mit_ increased by 13.5‐fold. The diastolic hsmL initially elongated because of the increase in preload, i.e. *F*_ext_, but gradually shortened due to the subsequent increases in diastolic Ca^2+^_cyt_. Throughout exercise, the magnitude of cell shortening increased. Following the increases in ATP‐consuming processes, mVO_2_ increased by 3.35 times during exercise (Fig. 7*E*). ATP_cyt_ was maintained nearly constant, at the expense of a decrease in PCr_cyt_ (Fig. 7*F, G*). When Ca^2+^_mit_‐dependent regulations of PDHC, ICDH and OGDH were silenced, NADH_mit_ change shifted downward (grey dashed line in Fig. 7*A*), suggesting that an exercise‐induced Ca^2+^_mit_ increase contributes to maintaining NADH_mit_. On the other hand, silencing the regulation did not alter mVO_2_, ATP_cyt_ and PCr_cyt_; note that the time courses with silenced regulation (grey lines in Fig. 7*A, E–G*) almost overlapped with those of controls. Upon exercise application, both NADH_mit_ consumption and production fluxes increased (Fig. 8*A, B*), with the consumption flux increasing earlier, as revealed by the flux difference plot (Fig. 8*C*). This caused biphasic NADH_mit_ change (Fig. 7*A*), as seen with the stimulus frequency increase protocols (see Fig. 6). Silencing the Ca^2+^_mit_‐dependent regulation of dehydrogenases shifted the flux difference plot downward during the early phase of the exercise (grey dashed line in Fig. 8*C*), making the NADH_mit_ change downward (grey dashed line in Fig. 7*A*).

The contribution of three exercise factors, namely heart rate, preload and contractility, to cardiac energetics was dissected by applying one factor at a time: cycle length shortening (Fig. 7*B*), *F*_ext_ increase (Fig. 7*C*), and β‐adrenergic receptor stimulation (Fig. 7*D*). Among the three factors, cycle length shortening had the greatest effect on NADH_mit_, Ca^2+^_mit_, mVO_2_ and PCr_cyt_. Since the increase in ATP consumption in a given period, i.e. 1 min, was much greater with cycle length shortening (Fig. 7*B*) than other factors, mVO_2_ was most affected by cycle length shortening (Fig. 7*E*), consistent with the well‐accepted idea that heart rate is the major determinant of mVO_2_ during exercise (see textbook by Herring & Paterson, 2018). Along with the mVO_2_ increase, flux of Complex I increased to consume NADH_mit_ (see Fig. 8*A*); thus the initial NADH_mit_ drop was most affected by cycle length shortening. Cycle length shortening and β‐adrenergic receptor stimulation increased Ca^2+^_cyt_ to a similar extent, but the Ca^2+^_mit_ increase, 9.03‐fold, was much greater with cycle length shortening than with β‐adrenergic receptor stimulation, 1.38‐fold. An increase in *F*_ext_ had little effect on Ca^2+^_cyt_, so Ca^2+^_mit_ remained unchanged. Accordingly, the contribution of Ca^2+^_mit_‐dependent regulation to increasing NADH_mit_ from the initial drop mostly depended on cycle length shortening.

**Figure 9:**
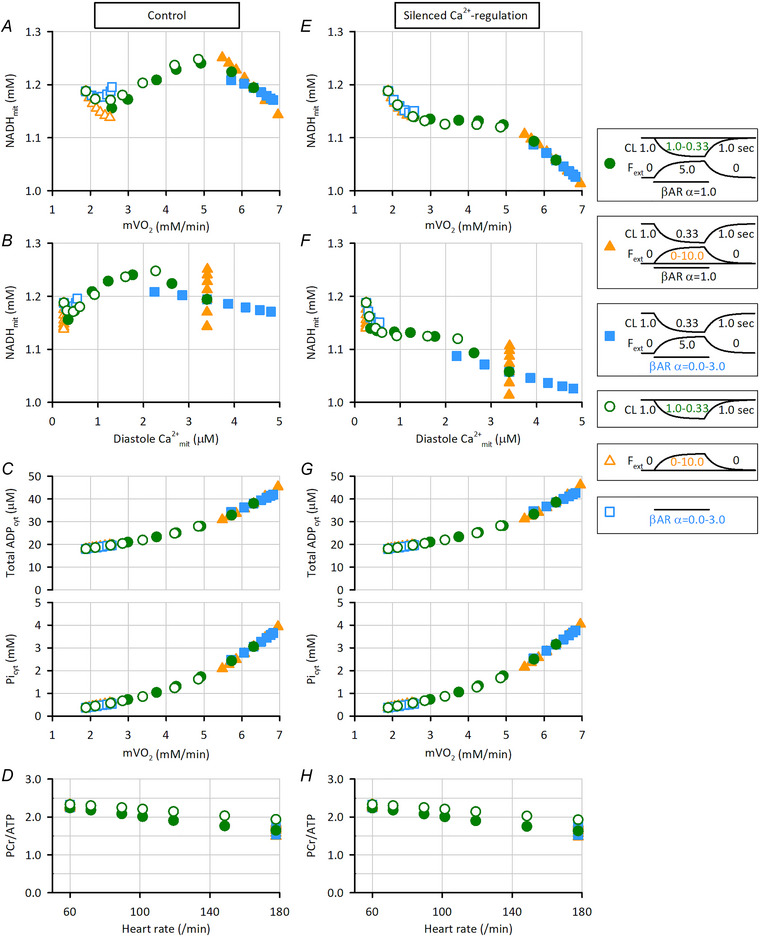
Dependence of NADH_mit_, total ATP_cyt_ and Pi_cyt_ on various workloads The workload increase by various factors was applied at 50 s with a time constant of 40 s. Values at 200 s after the onset of the workload increase were obtained without (*A–D*) or with (*E–H*) silenced Ca^2+^_mit_‐dependent regulation. Interventions were: cycle length (CL) shortening from 1.00 to 1.00, 0.83, 0.67, 0.59, 0.50, 0.40, 0.33 s with (green filled circles) or without (green open circles) *F*_ext_ increase to 5.00 plus application of α = 1.0 for β‐adrenergic stimulation, *F*_ext_ increase from 0.00 to 0.00, 0.90, 1.98, 3.34, 5.00, 7.25 and 10.00 with (orange filled triangles) or without (orange open triangles) cycle length shortening to 0.33 s plus application of α = 1.0 for β‐adrenergic stimulation, and application of α = 0.0, 0.5, 1.0, 1.5, 2.0, 2.5, 3.0 for β‐adrenergic stimulation with (sky‐blue filled squares) or without (sky‐blue open squares) cycle length shortening to 0.33 s plus *F*_ext_ increase to 5.0. *A* and *E*) dependence of NADH_mit_ on mVO_2_. *B* and *F*) dependence of NADH_mit_ on diastolic Ca^2+^_mit_. *C* and *G*) dependence of total ATP_cyt_ (upper) or Pi_cyt_ (lower) on mVO_2_. *D* and *H*) dependence of PCr/ATP on heart rate. The initial condition was the same as in Fig. 4.

Next, the three factors were varied in different combinations to simulate various extent of exercise, and the parameters 200 s after the application of the factors were obtained (Fig. 9). The cycle length shortenings from 1.00 to 1.00–0.33 s with (green filled circles) and without (green open circles) *F*_ext_ increase to 5.0 plus standard β‐adrenergic receptor stimulation (α = 1.0) covered a wide 3.35‐fold mVO_2_ change (Fig. 9*A*). Changing *F*_ext_ (orange triangles) and β‐adrenergic receptor stimulation (sky‐blue squares) did not produce as large an mVO_2_ difference as changing cycle length, regardless of whether it was combined with other factors (compare filled *vs*. open symbols). Meanwhile, the NADH_mit_ concentration at the end of various exercise regimes was 1.14–1.25 mm, −4.2 to +5.3% (*n* = 38, standard deviation (SD) = 2.6%) of that before starting exercise. For this small NADH_mit_ change, the increase in *F*_ext_ had a relatively large effect; see the steeper change of NADH_mit_ per mVO_2_ (orange triangles) than the one for different cycle lengths (green circles) or different α values in β‐adrenergic receptor stimulation (sky‐blue squares). Interestingly, in the mVO_2_ range of 2.5–5.0 mM/min, NADH_mit_ became larger with larger mVO_2_. This is due to the Ca^2+^_mit_‐dependent regulation of dehydrogenases (Fig. 9*B*). The NADH_mit_ concentration with different *F*_ext_ values aligned along the vertical lines, since stretch did not affect Ca^2+^_cyt_ nor Ca^2+^_mit_ but increased ATP consumption by contraction (see orange triangles in Fig. 9*B*). On the other hand, with different cycle lengths and different β‐adrenergic receptor stimulations, NADH_mit_ concentration showed three phases as Ca^2+^_mit_ increased: decrease, increase and decrease (see green circles and sky‐blue squares in Fig. 9*B*). At Ca^2+^_mit_ < ∼400 nm, NADH_mit_ decreased as Ca^2+^_mit_ increased because of increased ATP consumption by SERCA, *I*_pCa_ and contraction induced by larger Ca^2+^_cyt_. At Ca^2+^_mit_ of ∼400 nM–∼2.2 μM, NADH_mit_ increased because of Ca^2+^_mit_‐dependent activation of dehydrogenases; note that the Ca^2+^ affinity for PDHC, ICDH and OGDH are around several hundred nanomolar (see Online Supplementary Material Table S9 and McCormack et al., 1990; Rutter & Denton, 1988). The third NADH_mit_ decrease phase at Ca^2+^_mit_ > ∼2.2 μM was again a result of increased ATP consumption by SERCA, *I*_pCa_ and contraction, because dehydrogenase activities were saturated. Since an increase in mVO_2_ reflects an increase in ATP consumption, the products ADP_cyt_ and Pi_cyt_ increased as mVO_2_ increased, as expected (Fig. 9*C*).

When the Ca^2+^_mit_‐dependent regulation was silenced, NADH_mit_ shifted to a lower level, and the NADH_mit_ increasing phase disappeared (Fig. 9*E, F*). The overall NADH_mit_ difference increased to 0.0 to −14.7% (*n* = 38, SD = 4.0%; concentration at the end of various exercise regimes was 1.19–1.01 mm) of that before starting exercise, which was still small but larger than the values of −4.2 to +5.3% in the control model. On the other hand, silencing the Ca^2+^_mit_‐dependent regulation did not alter ADP_cyt_, Pi_cyt_ and PCr/ATP (Fig. 9*C* *vs*. *G*, *D*, *vs*. *H*).

These analyses revealed that the Ca^2+^_mit_‐dependent regulation made a small but remarkable contribution to the maintenance of NADH_mit_ during dynamic workload increases.

### Roles of uneven distributions of Ca^2+^_mit_ and Ca^2+^_SR_ handling proteins to cardiac energetics

**Figure 10:**
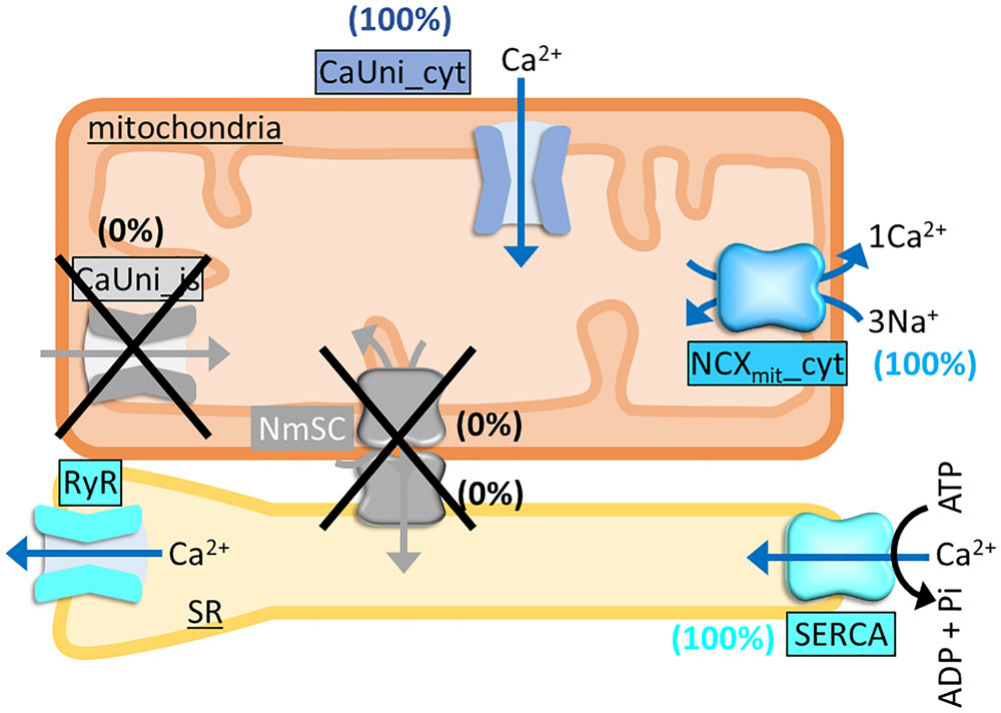
Localization settings of mitochondrial and SR Ca^2+^ handling proteins in the non‐MSI model The fractions of CaUni_js and NCX_mit_‐SERCA complex NmSC were set as 0%, and those of CaUni_cyt, NCX_mit__cyt and SERCA as 100%.

The contribution of spatial and functional couplings of mitochondria and SR via CaUni‐RyR and NCX_mit_‐SERCA were analysed using a non‐MSI model. In this model, NmSC and CaUni_js were removed and all NCX_mit_, CaUni and SERCA were set to face the cytoplasm (see Fig. 10). The basic characteristics of the non‐MSI model with cycle length of 1 s are presented in the Table 1 and Appendix Fig. A5. Action potential configuration and most ionic currents were not greatly affected (compare Appendix Fig. A2*A* and A5*A*). However, Ca^2+^_cyt_ transients, Ca^2+^_mit_, and therefore the extents of active force generation and hsmL shortening, as well as Ca^2+^ flux via RyR were diminished (see Appendix Fig. A5Da, De, Eb, Ea and Ba), resulting in lower ATP consumption (Table 1). Accordingly, mVO_2_ became smaller, NADH_mit_ and PCr_cyt_ became larger in the non‐MSI model than in the MSI model (Table 1).

**Figure 11:**
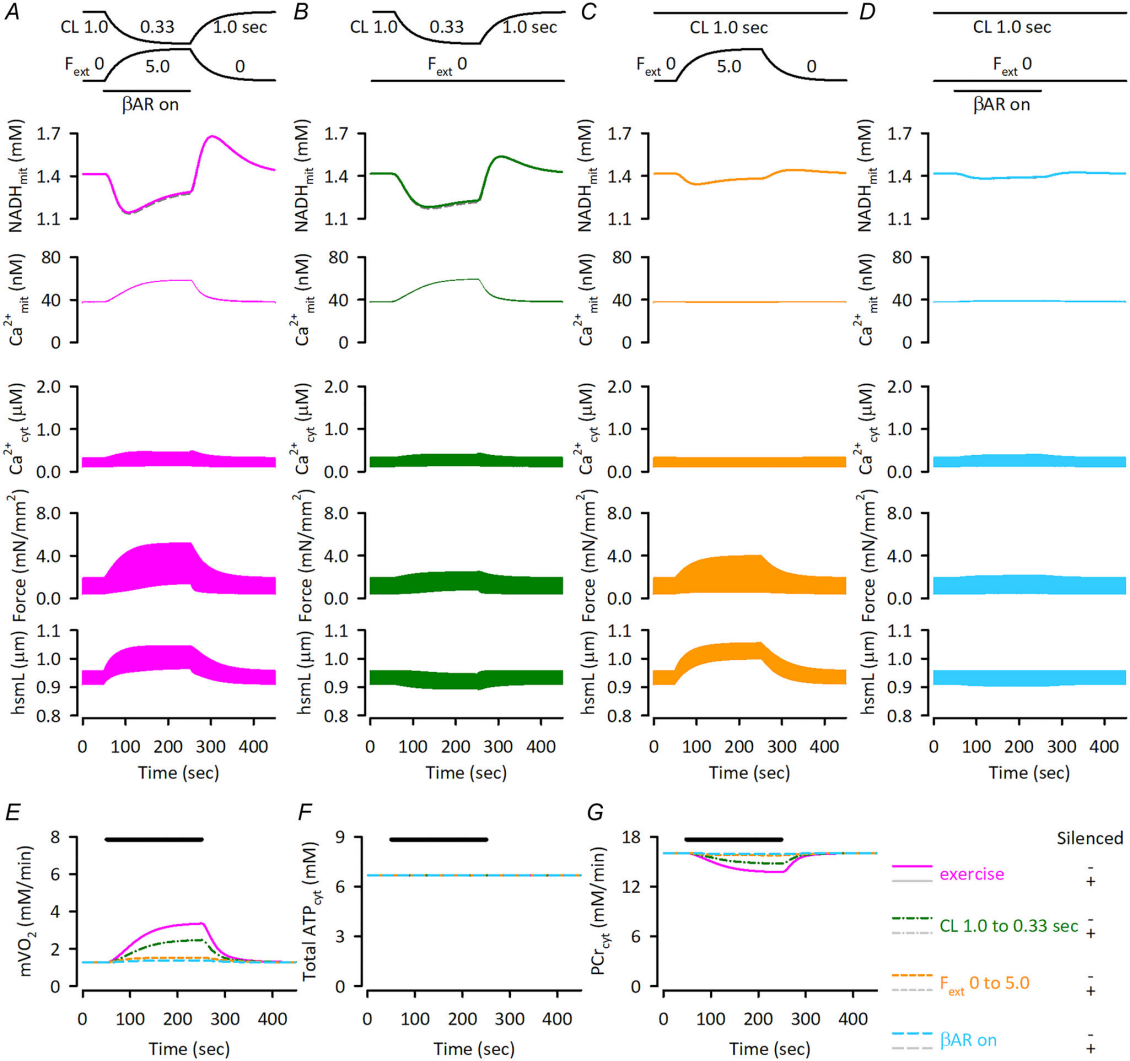
Responses of the non‐MSI model to dynamic changes of workload by various interventions *A*) responses of NADH_mit_, Ca^2+^_mit_, force and hsmL without (control; magenta continuous lines) or with (dashed grey lines) silenced Ca^2+^_mit_‐dependent regulation to exercise protocol, composed of cycle length (CL) shortening to 0.33 s, *F*_ext_ increase to 5.0, and application of α = 1.0 for β‐adrenergic stimulation. *B*) the responses when only cycle length was shortened to 0.33 s. *C*) the responses when only *F*_ext_ was increased to 5.0. *D*) the responses when only β‐adrenergic stimulation was applied, α = 1.0. The dashed grey lines are with silenced Ca^2+^_mit_‐dependent regulation (*B–D*). *E–G*) responses of mVO_2_ (*E*), total ATP_cyt_ concentration (*F*), and PCr_cyt_ concentration (*G*) to dynamic workload changes by exercise (magenta continuous lines), cycle length shortening to 0.33 s (green dotted‐dashed lines), *F*_ext_ increase to 5.0 (orange dashed lines), and application of α = 1.0 for β‐adrenergic stimulation (sky‐blue long dashed lines). Simulation results with silenced Ca^2+^_mit_‐dependent regulation are shown as grey lines, though they overlap with those with the control regulations. The initial condition of the non‐MSI model, isotonic contraction without applying *F*_ext_, is shown in the Online Supplementary Material Table S14. Then the same protocols were applied as in Fig. 7.

Then responses of cardiac energetics to dynamic workload transition were investigated. Exercise protocol application to the non‐MSI model induced biphasic NADH_mit_ change, an initial decrease followed by an increase (Fig. 11*A*), as observed in the control MSI model (see Fig. 7A). However, the decrease was larger and the increase was slower than in the MSI model, thus NADH_mit_ did not return to its pre‐exercise level despite the 1.88 times lower mVO_2_ during exercise (Fig. 11*E*; compare with Fig. 7*E*). The lower mVO_2_ in the non‐MSI model was due to the smaller increases in Ca^2+^_cyt_ transients, Ca^2+^_mit_, active force generation and hsmL shortening (see Fig. 11*A*; compare with Fig. 7*A*). The effects of changing the three factors one at a time on Ca^2+^_cyt_ transients, active force generation and hsmL shortening were smaller than in the MSI model (Fig. 11*B‐D*). Note that pre‐exercise diastolic Ca^2+^_mit_ concentration in the non‐MSI model was 36 nm, 6.91 times lower than that in the MSI model, and Ca^2+^_mit_ increased to only 57 nm during exercise, which was far below the Ca^2+^_mit_ affinity of dehydrogenases. Accordingly, silencing Ca^2+^_mit_‐dependent regulation had a negligible effect in the non‐MSI model.

**Figure 12:**
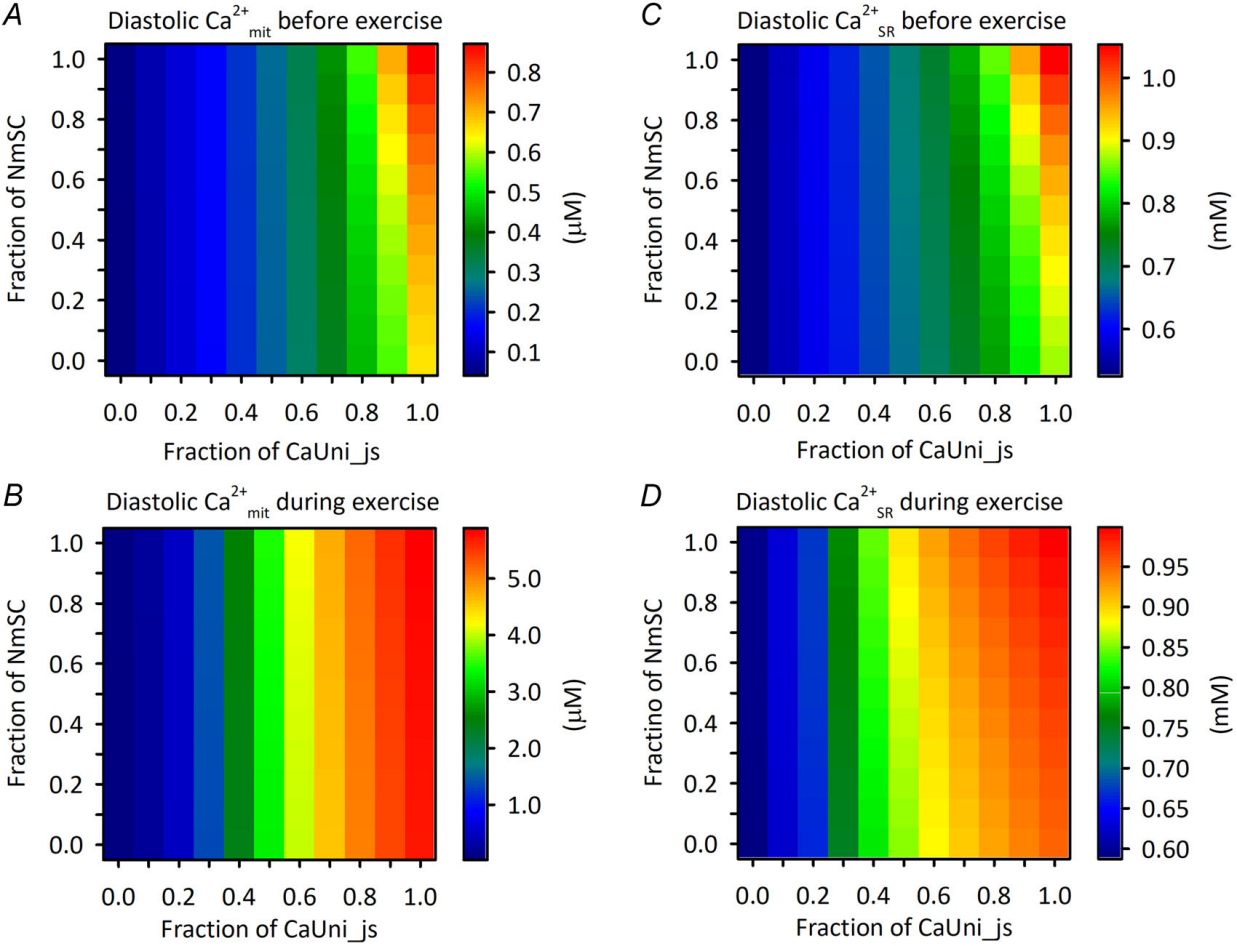
Effects of varying fractional ratio of CaUni_js and NmSC on Ca^2+^_mit_ and Ca^2+^_SR_ The fractional ratios of CaUni_js and NmSC were systematically varied from 0.0–1.0 in 0.1 increments. The model cell was stimulated with a cycle length of 1 s without applying *F*_ext_ under the condition of isotonic contraction for 20 min, and then the same exercise protocol as in Figs 7*A* and 11*A* was applied. The values just before and 200 s after the exercise onset were obtained. *A*) diastolic Ca^2+^_mit_ just before exercise onset. *B*) diastolic Ca^2+^_mit_ 200 s after exercise onset. *C*) diastolic Ca^2+^_SR_ just before exercise onset. *D*) diastolic Ca^2+^_SR_ 200 s after exercise onset.

**Figure 13:**
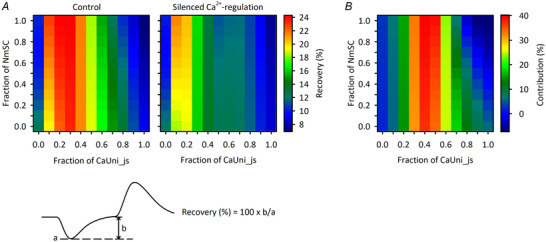
Contribution of Ca^2+^_mit_‐dependent regulation of dehydrogenases to NADH_mit_ dynamics The fractional ratios of CaUni_js and NmSC were systematically varied as Fig. 12 and the exercise protocol in Fig. 7*A* was applied. *A*) recovery of NADH_mit_ from initial drop without (left) and with (right) silenced Ca^2+^_mit_‐dependent regulation. Recovery of NADH_mit_ was calculated as 100 × *b*/*a* (see lower panel). *B*) contribution of Ca^2+^_mit_‐dependent regulation of dehydrogenases. The contribution (%) was calculated as; $\mathrm{Contribution}\;\left(\%\right)=\frac{\left(\mathrm{Recovery}\;\left(\%\right)\;\mathrm{in}\;“\mathrm{Control}”-\;\mathrm{Recovery}\;\left(\%\right)\;\mathrm{with}\;“\mathrm{silenced}\;{\mathrm{Ca}}^{2+}-\;\mathrm{regulation}”\right)}{\mathrm{Recovery}\;(\%)\;\mathrm{in}\;“\mathrm{Control}”}\times 100.$

Finally, the roles of the uneven distributions of Ca^2+^_mit_ and Ca^2+^_SR_ handling proteins on cardiomyocyte functions were investigated. The fractional ratios of CaUni_js and NmSC to total CaUni and NCX_mit_, respectively, were systematically varied from 0.0–1.0 in 0.1 increments. The exercise protocols were then applied to the models after stimulation with a cycle length of 1 s for 20 min. Note that the combination of both CaUni_js and NmSC fractions as 0.0 corresponds to the non‐MSI model described above (Figs 10, 11). It was clearly demonstrated that diastolic Ca^2+^_mit_ and Ca^2+^_SR_ concentrations strongly depend on CaUni_js fraction, regardless of exercise application (Fig. 12*A–D*). When the CaUni_js fractions were 0.0 and 0.1, i.e. the percentages of CaUni_js to total CaUni were 0% and 10%, respectively, the diastolic Ca^2+^_mit_ during exercise was below 400 nm, which was the threshold for the Ca^2+^_mit_‐dependent regulation to become predominant, as shown in the Fig. 9*A* and *B*. The NmSC fraction also had positive relationships with Ca^2+^_mit_ and Ca^2+^_SR_ concentrations, though the impacts were smaller than with the CaUni_js fraction (Fig. 12*A–D*). In order to evaluate the contribution of the uneven distributions of Ca^2+^_mit_ and Ca^2+^_SR_ handling proteins to the Ca^2+^_mit_‐dependent regulation of dehydrogenases during exercise, the percentage of NADH_mit_ recovery at the end of the exercise protocol from the initial drop was obtained (Fig. 13). For most combinations of CaUni_js and NmSC fractions, the NADH_mit_ recovery was greater in the model with Ca^2+^_mit_‐dependent regulation than in the one with the silenced regulation (Fig. 13*A*). The contribution of Ca^2+^_mit_‐dependent regulation was expressed as the difference in NADH_mit_ recovery between without and with silenced regulation. As expected, the contribution was minor for CaUni_js fractions of 0.0 and 0.1, since the Ca^2+^_mit_ concentration was below the threshold level for the regulation. The contribution also became smaller for larger fractions of CaUni_js. This is because Ca^2+^_mit_‐dependent regulation of dehydrogenases was already saturated yet ATP‐consuming processes were still increased by the increase in Ca^2+^_cyt_. Accordingly, the NADH production increase was obscured by the NADH consumption increase. The maximum contribution of ∼40% was obtained with a CaUni_js fraction of 0.4. The standard setting of the control MSI model – CaUni_js and NmSC fractions of 0.5 and 0.7, respectively, values that were adopted from experiments – showed near‐maximal contribution, 36.9% (Fig. 13*B*).

## Discussion

It has been suggested that distinct spatial distributions of the Ca^2+^_mit_ handling proteins MCU and NCLX, and their coupling with the Ca^2+^_SR_ handling proteins RyR and SERCA, respectively, contribute to efficient SR‐mitochondria Ca^2+^ signalling in cardiomyocytes (De La Fuente et al., 2016, 2018; Takeuchi & Matsuoka, 2022). However, there was little information on how efficient the signalling is, nor on how it contributes to ventricular myocyte functions. In the present study, we developed a new Integrated Human Ventricular Cell Model considering the spatial and functional couplings of Ca^2+^_mit_ and Ca^2+^_SR_ handling proteins, and successfully simulated excitation‐contraction‐energetics coupling. Quantitative model analyses revealed that uneven distributions of the handling proteins contribute to Ca^2+^_mit_ accumulation particularly during exercise, which promotes more stability of NADH_mit_ through activating NADH‐producing dehydrogenases. Furthermore, the analyses revealed that the uneven distribution of the handling proteins, uncovered by *in vitro* experiments (De La Fuente et al., 2016; Takeuchi & Matsuoka, 2022), optimizes the effect of Ca^2+^_mit_ ‐dependent regulation of dehydrogenases to stabilize NADH_mit_.

Among the three factors comprising exercise, changing cycle length drastically affected Ca^2+^_mit_, which was attributable to faster CaUni flux than NCX_mit_ flux. That is, with longer cycle length, there was enough time left for the slower NCX_mit_ to extrude more Ca^2+^_mit_. As the cycle length was shortened, the next stimulus came before sufficient amounts of Ca^2+^_mit_ was extruded, resulting in a staircase‐like Ca^2+^_mit_ accumulation. Although it is still controversial whether Ca^2+^_mit_ oscillates beat‐by‐beat, most reports in the literature agree that Ca^2+^_mit_ accumulates with shorter stimulus cycle length (Brandes & Bers, 2002; Jo et al., 2006; Lu et al., 2013; Maack et al., 2006; Mason et al., 2020; Wust et al., 2017).

Extensive simulation analyses of systematically varying the distributions of Ca^2+^_mit_ and Ca^2+^_SR_ handling proteins revealed that the fraction of CaUni_js greatly affects the extent of Ca^2+^_mit_ accumulation during exercise; i.e. Ca^2+^_mit_ concentration increased 6.76–8.73 times assuming all CaUni faced the JS, while only 1.57–1.96 times assuming all CaUni faced the cytoplasm (Fig. 12). The role of CaUni_js in efficient Ca^2+^_mit_ accumulation is well comparable to the model estimation by Maack et al. (2006) showing that the introduction of a hypothetical extra‐mitochondrial Ca^2+^ microdomain and increasing the number and/or amplitude of Ca^2+^ increase pulses effectively accumulate Ca^2+^_mit_. Interestingly, a larger fraction of NmSC resulted in a slight increase in Ca^2+^_mit_ for each given fraction of CaUni_js (Fig. 12*A*). This is explained as follows. Since NmSC supplies Ca^2+^ from mitochondria to SR, a larger NmSC fraction facilitates Ca^2+^ movement from mitochondria, increasing Ca^2+^_SR_ (Fig. 12*C*). Upon stimulation, more Ca^2+^ is hence released from the SR via RyR, and CaUni_js flux is then increased and Ca^2+^_mit_ replenished. Thus, the overall contribution of the uneven distribution of NCX_mit_ is additive to that of CaUni.

Although *in vitro* experiments clearly demonstrated that Ca^2+^ activates NADH‐producing dehydrogenases, it has been a matter of controversy whether Ca^2+^‐dependent regulation is required for metabolite constancy under *in vivo* physiological conditions (see reviews by Aon & Cortassa, 2012; Beard & Kushmerick, 2009; Glancy & Balaban, 2012; Korzeniewski, 2017; Saks et al., 2006; Takeuchi & Matsuoka, 2020). In fact, mice lacking MCU exhibited nearly normal cardiac function, including energy metabolism, with the exception of abrupt cardiac responses to catecholamine stimulation (Kwong et al., 2015; Luongo et al., 2015; Wu et al., 2015). We previously demonstrated, using a detailed model of mitochondria connected with simplified ATP consumption, that the contribution of Ca^2+^ to steady state metabolite constancy at various workloads is small when the physiological metabolic substrate composition is set as for *in vivo* measurements (Saito et al., 2016). In the present Integrated Human Ventricular Cell Model, the Ca^2+^_mit_‐dependent regulation accelerated NADH_mit_ recovery from the initial drop during exercise, with a contribution of about 36.9%. The quasi‐steady state NADH_mit_ during the exercise protocol was maintained in a narrow range of −4.2 to +5.3% of the resting level (SD = 2.6%) in the control MSI model, and the variation was increased to 0 to −14.7% (SD = 4.0%) when Ca^2+^_mit_‐dependent regulation of dehydrogenases was silenced. The decrease in SD suggested that the contribution of the Ca^2+^_mit_‐dependent regulation is 35% ((4.0 − 2.6)/4.0 × 100). Accordingly, NADH_mit_ became more stable in the presence of the regulation (Figs 7 and 9). The model predicted that Ca^2+^_mit_‐dependent regulation would be effective only when Ca^2+^_mit_ increased to a certain level, i.e. > 400 nm (Fig. 9). This is in line with experimental findings that MCU knockout phenotypes became apparent only when cells were challenged with catecholamine (Kwong et al., 2015; Luongo et al., 2015; Wu et al., 2015).

As mentioned above, NADH_mit_ change during exercise was kept relatively small even in the absence of Ca^2+^_mit_‐dependent activation of dehydrogenases; see almost constant NADH_mit_, especially in the range of 2–5 mM/min mVO_2_ (Fig. 9*E*). What contributes to the stability of NADH_mit_? One candidate regulator is Pi, an ATP hydrolysis product. Although early experiments reported a constant Pi concentration during workload transition (Katz et al., 1989), Wu et al. (2008) succeeded in detecting the change in Pi concentration during workload transition in *in vivo* canine hearts using ^31^P MRS combined with model simulations. They estimated that the Pi concentration was 0.29 mm at baseline and reached 2.3 mm during workload increase. Our model analyses yielded comparable but slightly higher Pi_cyt_ change; i.e. 0.37 mm at baseline, reaching 3.05 mm during exercise. This Pi entered the mitochondria, and allosterically activated OGDH, as reported experimentally (Rodriguez‐Zavala et al., 2000), stabilizing NADH_mit_ levels. It should be noted that allosteric activation by Pi_mit_ of Complex III of the electron transport chain, which was considered in our previous model (Saito et al., 2016) but was subsequently refuted experimentally (Bazil et al., 2016; Vinnakota, Bazil et al., 2016), has been removed in the present model. Another mechanism for NADH_mit_ stability is feedback regulation of dehydrogenases by the substrate NADH/product NAD^+^. As the substrate NADH is consumed and the product NAD^+^ increases, then the fluxes of dehydrogenases should become slowed, preventing further decrease in NADH. In any case, the NADH_mit_ stability should not be dependent solely on one regulatory mechanism, but on multiplex mechanisms.

In the present simulation analysis of exercise, mVO_2_ increased 3.69‐fold at maximum, which was comparable to values in the literature in humans (Binak et al., 1967; Heiss et al., 1976). At this exercise intensity, ATP_cyt_ concentration remained almost constant at the expense of PCr_cyt_ decrease, resulting in a decline of PCr/ATP (see Fig. 9*D*). Note that the extent of PCr/ATP decrease during standard exercise, 29.4%, was larger than the model‐estimate by Bakermans et al. (2017), 10%, probably due to the higher workload in our model; mVO_2_ was 6.31 mM/min in our model *vs*. 5 mM/min in Bakermans et al. (2017). The ATP_cyt_ concentration and the extent of the decrease in PCr_cyt_ and increases in Pi_cyt_ and total ADP_cyt_, and thus the decrease in PCr/ATP, were all unaffected by silencing Ca^2+^_mit_‐dependent regulation (Fig. 7*F, G* and Fig. 9*C, D, G, H*). These results suggest that Ca^2+^_mit_‐dependent regulation has negligible effects on phosphate metabolites and ATP hydrolysis potential, and that adaptation of ATP supply to increased demand may proceed even in the absence of NADH_mit_ change. The hypothesis is comparable to the report by Tran et al. (2015), who demonstrated using an excitation‐contraction‐energetics coupling model with simplified mitochondrial function that introducing Ca^2+^_mit_‐dependent activation of dehydrogenase increased NADH_mit_, yet did not affect ATP production. The contribution of Ca^2+^_mit_‐dependent activation of dehydrogenases to ATP production should become apparent under more extreme physiological and pathophysiological conditions accompanying excessive ATP utilization, such as tachycardia and ventricular fibrillation (Badeer & Feisal, 1965; Kusuoka et al., 1992). Given our previous findings that metabolic substrate composition is an important determinant of the contribution of Ca^2+^_mit_‐dependent activation of dehydrogenases (Saito et al., 2016), conditions accompanying metabolic perturbations would also be candidate situations when the Ca^2+^_mit_‐dependent activation of dehydrogenases plays a more significant role. In fact, there are several inherited and acquired disorders associated with deficiencies in mitochondrial enzymes and therefore with metabolite remodelling (see reviews by Lopaschuk et al., 2021; Pell et al., 2016; Stanley et al., 2005). We tentatively focused on MDH, whose genetic mutations were recently reported (Priestley et al., 2022). When exercise simulation was performed with MDH amplitude reduced to 0.1%, the extent of exercise‐induced PCr_cyt_ reduction was 43.3%, which was greater than 29.6% for 100% MDH. Interestingly, when the Ca^2+^_mit_‐dependent regulation of dehydrogenases was silenced, the PCr_cyt_ reduction became even greater, 55.2%. Similar results were obtained when all metabolic substrates concentrations were reduced to 15%; i.e. the extent of PCr_cyt_ reduction by exercise was 39.9% and 53.6% in the presence and absence of the Ca^2+^_mit_‐dependent regulation. It would be interesting to evaluate excitation‐contraction‐mitochondrial energetics coupling by considering the remodelling of Ca^2+^ handling proteins and energetics‐related proteins as well as of metabolite composition. However, these are beyond the scope of the present study.

## Limitation of the model

Our model has several limitations. First, we compared our model analyses data with human *in vivo* experimental data. Since the model is of a single ventricular myocyte, there are some deviations. For example, the mVO_2_ value calculated form the model, 1.88 mM/min at stimulation cycle length of 1 s, is approximately half the value estimated from human data; i.e. 3.57 mM/min (calculated from ∼8 ml/100 g/min at rest (Strauer, 1979)) and can increase by ∼10‐fold at maximal exercise (see also textbook by Herring & Paterson, 2018). Factors which were not included in this single ventricular myocyte model such as afterload, oxygen delivery, involvements of atria, and so on, should affect the results. Most recently, Sturgess et al. (2024) reported mechanistic insights into myocardial perfusion and oxygen delivery during exercise, using a sophisticated model that integrates whole‐body cardiovascular haemodynamics, cardiac mechanics and myocardial work. Our integrated cardiomyocyte model analyses in combination with whole‐body modelling analyses would provide more accurate and interesting information, albeit at an enormous computational cost.

Second, we assume an extremely strong coupling between mitochondria and SR, as was done in the HL‐1 cell model, since the details of coupling mechanisms remain unresolved (Takeuchi & Matsuoka, 2022). The contribution of NmSC to Ca^2+^_mit_ and Ca^2+^_SR_ dynamics might be overestimated in the model.

Third, the present model calculates the ATP consuming steps according to the reported stoichiometry and does not consider the ATP‐dependence of SERCA, *I*_NaK_, *I*_pCa_, and myosin ATPase. In addition to this, we did not consider the ATP‐sensitive K^+^ channels at the plasma membrane and mitochondria that open upon ATP starvation (Noma, 1983; see also review Foster & Coetzee, 2016), making it impossible to simulate ATP‐starved conditions. Introduction of thermodynamically consistent models for ATP‐consuming components (Pan et al., 2019; Tran et al., 2010), as well as models for ATP‐sensitive K^+^ channels (Himeno et al., 2024; Zhou et al., 2009), and testing the ATP‐starving conditions such as ischemia‐reperfusion, would provide further insights into excitation‐contraction‐mitochondrial energetics coupling.

Fourth, the present model does not include glycolysis and β‐oxidation of fatty acid. However, the absence of these processes is unlikely to have much influence on the main conclusion. When the Ca^2+^_mit_‐dependent activation of ICDH and OGDH was silenced but that of PDHC was intact during the exercise simulation, the time course of NADH_mit_ almost overlapped with the dashed grey line in the Fig. 7*A*; the recovery of NADH_mit_ from its initial drop was 12% in both simulations. This is mainly due to the relatively small Ca^2+^‐sensitive component of PDHC (Saito et al., 2016). Therefore, the potential Ca^2+^_mit_ effects would not be greatly affected even when the PDHC contribution is changed by incorporating glycolysis and β‐oxidation of fatty acid. However, substrate usage of mitochondria is an important issue. It would be interesting to examine the effects of glucose‐fuelled *versus* fatty acid‐fuelled respiration on cardiac energetics during workload transition, but it is beyond the scope of the present study.

## Additional information

## Competing interests

The authors declare that they have no conflicts of interest with the contents of this article.

## Author contributions

A.T.: conceptualization, study design, software, data analyses, visualization, writing the original draft. S.M.: conceptualization, study design, software, data analyses, visualization, review & editing the originals draft. Both authors approved the final version of the manuscript, and agreed to be accountable for all aspects of the work in ensuring that questions related to the accuracy or integrity of any part of the work are appropriately investigated and resolved, and confirmed that both persons designated as authors qualify for authorship and all those who qualify for authorship are listed.

## Funding

This work was supported by JSPS KAKENHI grant Numbers 18K06869 (A.T.), 22K06841 (A.T.), 19H03400 (S. M.), and 23K24065 (S. M.), by Life Science Innovation Centre at University of Fukui (A.T.), Research Grants from the University of Fukui (FY 2023) (A.T.), and by the Kurata Grants of The Hitachi Global Foundation (A.T., grant Number 1511).

**Figure A1:**
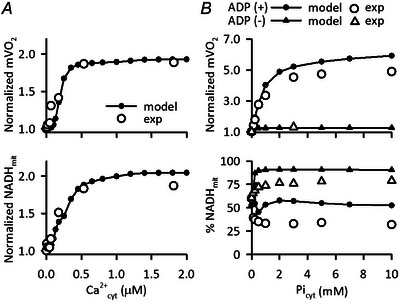
Dependence of mVO_2_ and NADH_mit_ on Ca^2+^_cyt_ and Pi_cyt_ in the isolated mitochondrial model *A*) dependence of Ca^2+^_cyt_. The isolated mitochondrial model was calculated for 1 h with Ca^2+^_cyt_ of 10^−9^−2.0 × 10^−3^ mM. The steady state values were obtained and normalized to those at 10^−9^ mM Ca^2+^_cyt_ (filled circles). Experimental data using isolated porcine cardiac mitochondria were from Territo et al. (2000). *B)* dependence of Pi_cyt_. The isolated mitochondrial model was serially applied with Pi_cyt_ of 10^−6^−10 mm and ADP_cyt_ of 1.3 mm at 110 s and 170 s, respectively. The values at 169 s (state IV) and 229 s (state III) were obtained. For NADH_mit_, the values were expressed as the percentage of the reduced form, i.e. NADH_mit_, to total NAD^+^_mit_ plus NADH_mit_ (filled triangles). For mVO_2_, the values were normalized to those at 10^−6^ mm Pi_cyt_ (filled circles). Experimental data using isolated porcine cardiac mitochondria were from Bose et al. (2003).

**Figure A2:**
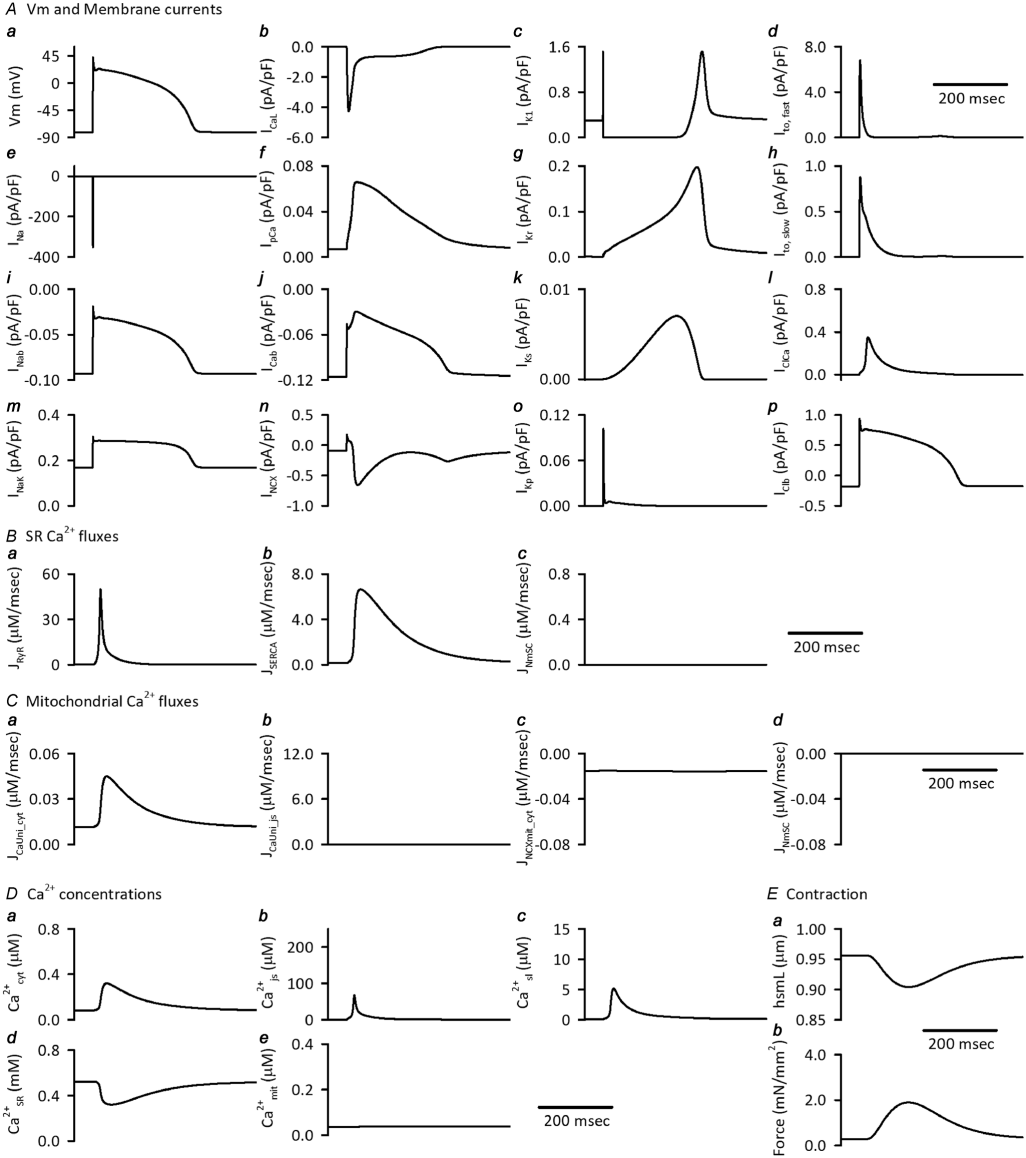
Configurations of action potential, major ionic currents, Ca^2+^ fluxes and Ca^2+^ concentrations in each compartment of the Integrated Human Ventricular Cell Model The standard initial condition of the model was obtained without applying *F*_ext_, i.e. 0.950 μm diastolic hsmL, under the condition of isotonic contraction, stimulated with a cycle length of 1 s for 30 min. The steady state values are listed in the Online Supplementary Material Table S14.

**Figure A3:**
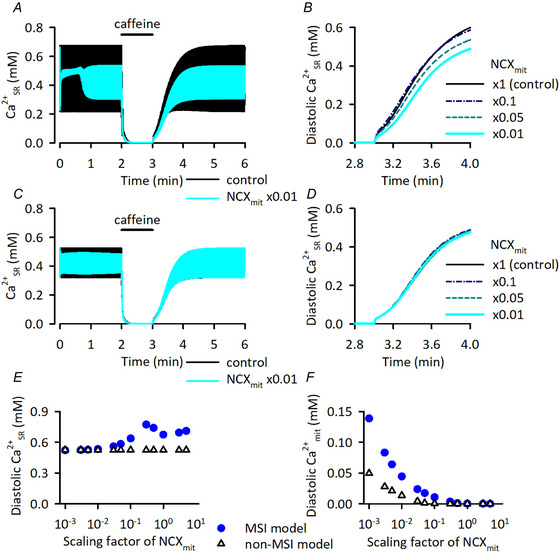
Effects of NCX_mit_ reduction on the responses to caffeine application and removal of the Integrated Human Ventricular Cell Model with and without mitochondria‐SR interaction *A*) time courses of Ca^2+^_SR_ concentration in the model with MSI. Black and blue lines represent data using the model with NCX_mit_ amplitude factor of ×1.0 (control) and ×0.01, respectively. *B*) same protocol was applied with various amplitude factors for NCX_mit_. The Ca^2+^_SR_ reuptake phase is magnified. *C* and *D*) the same protocols were applied to the model without MSI (non‐MSI), as in *A* and *B*, respectively. *E*) relationships between NCX_mit_ amplitude factor and diastolic Ca^2+^_SR_ at the end of the protocol, in the model with (control; blue filled circles) and without (open triangles) MSI. *F*) relationships between NCX_mit_ amplitude factor and diastolic Ca^2+^_mit_ at the end of the protocol, in the model with (control; blue filled circles) and without (open triangles) MSI.

**Figure A4:**
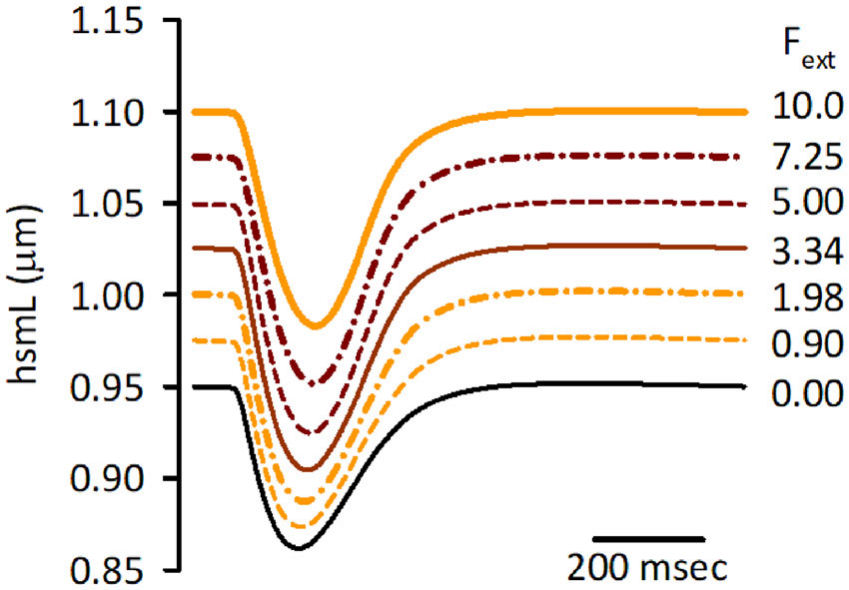
The hsmL shortenings with different *F*_ext_ in the Integrated Human Ventricular Cell Model The model cell was stimulated with a cycle length of 1 s with various *F*_ext_ values of 0.00–10.00; i.e. 0.950–1.100 μm diastolic hsmL. The hsmL shortenings at steady state were plotted.

**Figure A5:**
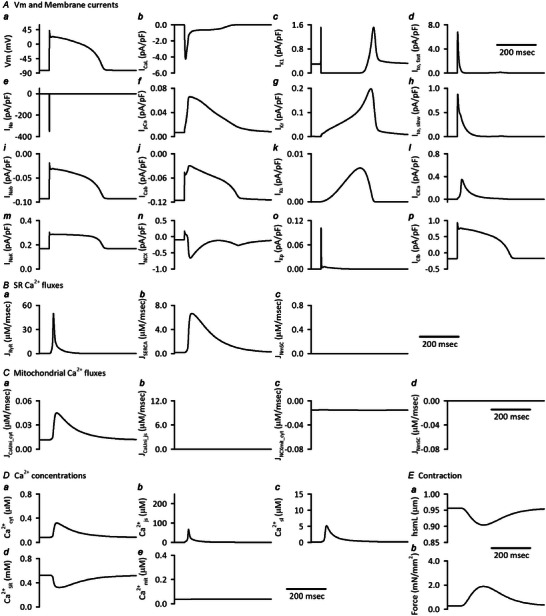
Configurations of action potential, major ionic currents, Ca^2+^ fluxes and Ca^2+^ concentrations in each compartment of the Integrated Human Ventricular Cell Model without mitochondria‐SR interaction (non‐MSI) The Integrated Human Ventricular Cell Model without mitochondria‐SR interaction (non‐MSI) was obtained by changing the fractions of NmSC and CaUni_js to 0, and then calculated for 20 min using the standard initial conditions of the MSI model. The steady state values are shown in the Online Supplementary Material Table S14.

## Supporting information




Peer Review History

Online Supplementary Material

## Data Availability

The source code is available on GitHub at https://github.com/atakeuti/IHVCM. The original contributions presented in the study are included in the article and in the Online Supplementary Material; further inquiries can be directed to the corresponding author.

## Appendix

Figures A1, A2, A3, A4, A5
