# Supplementary material for: A simulation study on the role of mitochondria‐sarcoplasmic reticulum Ca2+ interaction in cardiomyocyte energetics during exercise
Source: J Physiol. 2024 Oct 10;603(18):4921–49. doi: 10.1113/JP286054 (PMC12456394; doi:10.1113/JP286054)
Supplement: Supplementary file 2 — Online Supplementary Material [file TJP-603-4921-s002.pdf]

Online Supplementary Material

A simulation study on the role of mitochondria–sarcoplasmic reticulum Ca^{2+} interaction in cardiomyocyte energetics during exercise

Ayako Takeuchi, Satoshi Matsuoka
Department of Integrative and Systems Physiology, Faculty of Medical Sciences, and Life Science Innovation Center, University of Fukui, Fukui 910-1193, Japan

Description of the Integrated Human Ventricular Cell Model with/without mitochondria–SR interaction (MSI model/non-MSI model)

Table S1. Model variables and constants

R	Gas constant, 8.3143 C· mV· K^{-1}· $mmol^{-1}$
F	Faraday's constant, 96.4867 C· $mmol^{-1}$
T	Absolute temperature, 310.15 K
Vm	Membrane potential in mV
$\Delta\Psi$	Mitochondrial membrane potential in mV
hsmL	Half sarcomere length in μm
hsmX	Length composed of half of the thick filament and the free portion of the thin filament in μm
F_b	Cross-bridge force in mN· mm^{-2}
F_p	Force of parallel elastic component in mN· mm^{-2}
F_{ext}	External load
$[Ca^{2+}]_o$	Extracellular Ca^{2+} concentration, 1.8 mM
$[Ca^{2+}]_{cyt}$	Cytoplasmic Ca^{2+} concentration in mM
$[Ca^{2+}]_{JS}$	Junctional space Ca^{2+} concentration in mM
$[Ca^{2+}]_{SL}$	Subsarcolemmal space Ca^{2+} concentration in mM
$[Ca^{2+}]_{SR}$	Sarcoplasmic reticulum Ca^{2+} concentration in mM
$[Ca^{2+}]_{mit}$	Mitochondrial free Ca^{2+} concentration in mM
$[Ca^{2+}]_{totalmit}$	Mitochondrial total Ca^{2+} concentration in mM
$[Cl^{-}]_o$	Extracellular Cl^{-} concentration, 150 mM
$[Cl^{-}]_{cyt}$	Cytoplasmic Cl^{-} concentration, 15 mM
$[H^{+}]_{cyt}$	Cytoplasmic H^{+} concentration, $5.01187 \cdot 10^{-5}$ mM
$[H^{+}]_{mit}$	Mitochondrial H^{+} concentration in mM
$[K^{+}]_o$	Extracellular K^{+} concentration, 5.4 mM
$[K^{+}]_{cyt}$	Cytoplasmic K^{+} concentration, 120 mM
$[K^{+}]_{mit}$	Mitochondrial K^{+} concentration in mM
$[Mg^{2+}]_{cyt}$	Cytoplasmic free Mg^{2+} concentration, 1.0 mM

$[Mg^{2+}]_{mit}$	Mitochondrial free Mg^{2+} concentration, 0.38 mM
$[Na^{+}]_{o}$	Extracellular Na^{+} concentration, 140 mM
$[Na^{+}]_{cyt}$	Cytoplasmic Na^{+} concentration in mM
$[Na^{+}]_{JS}$	Junctional space Na^{+} concentration in mM
$[Na^{+}]_{SL}$	Subsarcolemmal space Na^{+} concentration in mM
$[Na^{+}]_{mit}$	Mitochondrial Na^{+} concentration in mM
$[totalADP]_{cyt}$	Total ADP concentration in cytoplasm in mM
$[freeADP]_{cyt}$	Free ADP concentration in cytoplasm in mM
$[MgADP]_{cyt}$	Mg^{2+}-bound ADP concentration in cytoplasm in mM
$[totalADP]_{mit}$	Total ADP concentration in mitochondria in mM
$[freeADP]_{mit}$	Free ADP concentration in mitochondria in mM
$[MgADP]_{mit}$	Mg^{2+}-bound ADP concentration in mitochondria in mM
$[ALA]_{mit}$	Mitochondrial alanine concentration, 1.0 mM
$[AMP]_{cyt}$	Cytoplasmic AMP concentration in mM
$[ASP]_{cyt}$	Cytoplasmic aspartate concentration, 3.0 mM
$[ASP]_{mit}$	Mitochondrial aspartate concentration in mM
$[totalATP]_{cyt}$	Total ATP concentration in cytoplasm in mM
$[freeATP]_{cyt}$	Free ATP concentration in cytoplasm in mM
$[MgATP]_{cyt}$	Mg^{2+}-bound ATP concentration in cytoplasm in mM
$[totalATP]_{mit}$	Total ATP concentration in mitochondria in mM
$[freeATP]_{mit}$	Free ATP concentration in mitochondria in mM
$[MgATP]_{mit}$	Mg^{2+}-bound ATP concentration in mitochondria in mM
$[totalAdenine]_{cyt}$	Cytoplasmic total adenine nucleotide concentration, 6.7 mM
$[totalAdenine]_{mit}$	Mitochondrial total adenine nucleotide concentration, 16.26 mM
$[CIT]_{cyt}$	Cytoplasmic citrate concentration, 0.3 mM
$[CIT]_{mit}$	Mitochondrial citrate concentration in mM
$[CoA]_{mit}$	Mitochondrial CoA concentration in mM
$[AcCoA]_{mit}$	Mitochondrial acetyl-CoA concentration in mM
$[ScCoA]_{mit}$	Mitochondrial succinyl-CoA concentration in mM
$[totalCoA]_{mit}$	Mitochondrial total CoA concentration, 0.3 mM
$[Cr]_{cyt}$	Cytoplasmic creatine concentration in mM
$[PCr]_{cyt}$	Cytoplasmic phosphocreatine concentration in mM
$[totalCr]_{cyt}$	Cytoplasmic total creatine concentration, 25 mM
$[Cytar]_{mit}$	Mitochondrial reduced cytochrome a concentration in mM
$[Cytao]_{mit}$	Mitochondrial oxidized cytochrome a concentration in mM
$[totalCyta]_{mit}$	Mitochondrial total cytochrome a concentration, 0.135 mM
$[Cytcr]_{mit}$	Mitochondrial reduced cytochrome c concentration in mM

$[Cytco]_{mit}$	Mitochondrial oxidized cytochrome c concentration in mM
$[totalCytc]_{mit}$	Mitochondrial total cytochrome c concentration, 0.27 mM
$[FUM]_{mit}$	Mitochondrial fumarate concentration in mM
$[GLU]_{cyt}$	Cytoplasmic glutamate concentration, 5.0 mM
$[GLU]_{mit}$	Mitochondrial glutamate concentration in mM
$[GDP]_{mit}$	Mitochondrial GDP concentration in mM
$[GTP]_{mit}$	Mitochondrial GTP concentration in mM
$[totalGuanine]_{mit}$	Mitochondrial total guanine nucleotide concentration, 1.0 mM
$[HCO_3^-]_{mit}$	Mitochondrial HCO_3^- concentration, 1 mM
$[ISOC]_{mit}$	Mitochondrial isocitrate concentration in mM
$[freeISOC]_{mit}$	Mitochondrial free isocitrate concentration in mM
$[MgISOC]_{mit}$	Mitochondrial Mg^{2+}-bound isocitrate concentration in mM
$[MAL]_{cyt}$	Cytoplasmic malate concentration, 1.0 mM
$[MAL]_{mit}$	Mitochondrial malate concentration in mM
$[NADH]_{mit}$	Mitochondrial NADH concentration in mM
$[NAD^+]_{mit}$	Mitochondrial NAD^+ concentration in mM
$[totalNAD]_{mit}$	Mitochondrial total NAD concentration, 2.97 mM
$[OAA]_{mit}$	Mitochondrial oxaloacetate concentration in mM
$[O_2]_{mit}$	Mitochondrial O_2 concentration, 0.24 mM
$[OG]_{cyt}$	Cytoplasmic 2-oxoglutarate concentration, 0.5 mM
$[OG]_{mit}$	Mitochondrial 2-oxoglutarate concentration in mM
$[Pi^-]_{cyt}$	Cytoplasmic Pi^- concentration in mM
$[Pi^-]_{mit}$	Mitochondrial Pi^- concentration in mM
$[PYR]_{cyt}$	Cytoplasmic pyruvate concentration, 0.3 mM
$[PYR]_{mit}$	Mitochondrial pyruvate concentration in mM
$[SUC]_{mit}$	Mitochondrial succinate concentration in mM
$[UQH_2]_{mit}$	Mitochondrial ubiquinol concentration in mM
$[UQ]_{mit}$	Mitochondrial ubiquinone concentration in mM
$[totalUQ]_{mit}$	Mitochondrial total ubiquinone concentration, 1.35 mM
$B_{maxCamit}$	Maximum concentration of Ca^{2+} buffering molecules in mitochondria, 2.0 mM
$K_{dCabuffmit}$	Binding constant to Ca^{2+} buffering molecules in mitochondria, 0.001 mM
$K_{dATPcyt}$	ATP magnesium binding constant in cytoplasm, 0.024 mM
$K_{dATPmit}$	ATP magnesium binding constant in mitochondria, 0.017 mM
$K_{dADPcyt}$	ADP magnesium binding constant in cytoplasm, 0.347 mM
$K_{dADPmit}$	ADP magnesium binding constant in mitochondria, 0.282 mM
K_{dISOC}	Isocitrate magnesium binding constant, 1.92 mM
ΔpH	pH gradient in mV

Δp	Proton motive force in mV
pH_{cyt}	Cytoplasmic pH, 7.3
pH_{mit}	Mitochondrial pH
$rbuffer_{mit}$	H^+ buffering capacity coefficient for mitochondria
E_{mN}	NAD redox potential in mV
$E_{mN,0}$	NAD standard redox potential, −320 mV
E_{mU}	Ubiquinone redox potential in mV
$E_{mU,0}$	Ubiquinone standard redox potential, 40 mV
E_{mc}	Cytochrome c redox potential in mV
$E_{mc,0}$	Cytochrome c standard redox potential, 250 mV
E_{ma}	Cytochrome a redox potential in mV
$E_{ma,0}$	Cytochrome a standard redox potential, 540 mV

Table S2. Cell property

	Abbreviation	Value	Unit
Total cellular volume	Vol_i	33000	μm^3
Cytoplasmic volume	Vol_{cyt}	$0.65 \cdot Vol_i$	μm^3
Junctional space volume	Vol_{JS}	$5.39 \cdot 10^{-4} \cdot Vol_i$	μm^3
Subsarcolemmal space volume	Vol_{SL}	$0.02 \cdot Vol_i$	μm^3
SR volume	Vol_{SR}	$0.035 \cdot Vol_i$	μm^3
Mitochondrial volume	Vol_{mit}	$0.23 \cdot Vol_i$	μm^3
Cell capacitance	Cm	138.1	pF
Mitochondrial inner membrane capacitance	Cmit	1.0	$mM \cdot mV^{-1}$
Fractional current of junctional space	F_{JS}	0.11	
Fractional current of subsarcolemmal space	F_{SL}	$1-F_{JS}$	
Fractional current through I_{CaL} of junctional space	F_{JSCa}	0.9	
Fractional current through I_{CaL} of subsarcolemmal space	F_{SLCa}	$1-F_{JSCa}$	

Table S3. Ion fluxes at junctional and subsarcolemmal membrane

I_{Na}; Voltage-dependent Na^+ current

	Abbreviation	Value	Unit
conductance	G_{Na}	23.0	$nS \cdot pF^{-1}$

$$m_{ss} = \frac{1}{\left(1 + \exp\left(-\frac{56.86 + Vm}{9.03}\right)\right)^2}$$

$$\tau_m = 0.1292 \cdot \exp\left(-\left(\frac{Vm + 45.79}{15.54}\right)^2\right) + 0.06487 \cdot \exp\left(-\left(\frac{Vm - 4.823}{51.12}\right)^2\right)$$

$$h_{ss} = \frac{1}{\left(1 + \exp\left(\frac{Vm + 71.55}{7.43}\right)\right)^2}$$

$$\alpha_h = \begin{cases} 0.057 \cdot \exp\left(-\frac{Vm + 80}{6.8}\right), & Vm < -40\ mV \\ 0, & \text{otherwise} \end{cases}$$

$$\beta_h = \begin{cases} 2.7 \cdot \exp(0.079 \cdot Vm) + 3.1 \cdot 10^5 \cdot \exp(0.3485 \cdot Vm), & Vm < -40\ mV \\ \frac{0.77}{0.13 \cdot \left(1 + \exp\left(-\frac{Vm + 10.66}{11.1}\right)\right)}, & \text{otherwise} \end{cases}$$

$$\tau_h = \frac{1}{\alpha_h + \beta_h}$$

$$j_{ss} = \frac{1}{\left(1 + \exp\left(\frac{Vm + 71.55}{7.43}\right)\right)^2}$$

$$\alpha_j = \begin{cases} \frac{\left(-2.5428 \cdot 10^4 \cdot \exp(0.2444 \cdot Vm) - 6.948 \cdot 10^{-6} \cdot \exp(-0.04391 \cdot Vm)\right) \cdot (Vm + 37.78)}{1 + \exp\left(0.311 \cdot (Vm + 79.23)\right)}, & Vm < -40\ mV \\ 0, & \text{otherwise} \end{cases}$$

$$\beta_j = \begin{cases} \frac{0.02424 \cdot \exp(-0.01052 \cdot Vm)}{1 + \exp\left(-0.1378 \cdot (Vm + 40.14)\right)}, & Vm < -40\ mV \\ \frac{0.6 \cdot \exp(0.057 \cdot Vm)}{1 + \exp\left(-0.1 \cdot (Vm + 32)\right)}, & \text{otherwise} \end{cases}$$

$$\tau_j = \frac{1}{\alpha_j + \beta_j}$$

$$\frac{dm}{dt} = \frac{m_{ss} - m}{\tau_m}$$

$$\frac{dh}{dt} = \frac{h_{ss} - h}{\tau_h}$$

$$\frac{dj}{dt} = \frac{j_{ss} - j}{\tau_j}$$

$$I_{Na\_JS} = F_{JS} \cdot G_{Na} \cdot m^3 \cdot h \cdot j \cdot \left(Vm - E_{Na\_JS}\right)$$

$$I_{Na\_SL} = F_{SL} \cdot G_{Na} \cdot m^3 \cdot h \cdot j \cdot \left(Vm - E_{Na\_SL}\right)$$

$$I_{Na} = I_{Na\_JS} + I_{Na\_SL}$$

I_{Nab}; Background Na^+ current

	Abbreviation	Value	Unit

conductance	G_{Nab}	$5.97 \cdot 10^{-4}$	$nS \cdot pF^{-1}$

$$I_{Nab\_JS} = F_{JS} \cdot G_{Nab} \cdot (Vm - E_{Na\_JS})$$
$$I_{Nab\_SL} = F_{SL} \cdot G_{Nab} \cdot (Vm - E_{Na\_SL})$$
$$I_{Nab} = I_{Nab\_JS} + I_{Nab\_SL}$$

I_{NaK}; Sarcolemmal Na^{+}/K^{+} pump current

	Abbreviation	Value	Unit
maximum velocity	$V_{max\_INaK,0}$	1.62	$pA \cdot pF^{-1}$
dissociation constant	Km_{Nai}	11.0	mM
dissociation constant	Km_{Ko}	1.5	mM

$$\sigma = \frac{\exp\left(\frac{[Na^+]_o}{67.3}\right) - 1}{7}$$

$$f_{NaK} = \frac{1}{1 + 0.1245 \cdot \exp\left(-0.1 \cdot Vm \cdot \frac{F}{RT}\right) + 0.0365 \cdot \sigma \cdot \exp\left(-Vm \cdot \frac{F}{RT}\right)}$$

$$I_{NaK\_JS} = F_{JS} \cdot \frac{V_{max\_INaK} \cdot f_{NaK}}{1 + \left(\frac{Km_{Na_i}}{[Na^+]_{JS}}\right)^4} \cdot \frac{[K^+]_o}{[K^+]_o + Km_{K_o}}$$

$$I_{NaK\_SL} = F_{SL} \cdot \frac{V_{max\_INaK} \cdot f_{NaK}}{1 + \left(\frac{Km_{Na_i}}{[Na^+]_{SL}}\right)^4} \cdot \frac{[K^+]_o}{[K^+]_o + Km_{K_o}}$$

$$I_{NaK} = I_{NaK\_JS} + I_{NaK\_SL}$$

For β-adrenergic stimulation

$$AI_{NaK} = 1 + \alpha \cdot 0.2, \text{where } \alpha = 1.0 \text{ for standard } \beta - \text{adrenergic stimulation}$$
$$V_{max\_INaK} = AI_{NaK} \cdot V_{max\ \_INaK,0}$$

ATP consumption rate in $mM \cdot msec^{-1}$

$$\frac{dATPuse\_I_{NaK}}{dt} = \frac{I_{NaK} \cdot Cm}{(Vol_{cyt} + Vol_{JS} + Vol_{SL}) \cdot F}$$

I_{Kr}; Rapidly activating K^{+} current

	Abbreviation	Value	Unit
conductance	G_{Kr}	0.035	$nS \cdot pF^{-1}$

$$xkr_{ss} = \frac{1}{1 + \exp\left(-\frac{Vm + 10}{5}\right)}$$

$$rkr = \frac{1}{1 + \exp\left(\frac{Vm + 74}{24}\right)}$$

$$\frac{dxkr}{dt} = \frac{xkr_{ss} - xkr}{\tau_{xkr}}$$

$$I_{Kr\_JS} = F_{JS} \cdot G_{Kr} \cdot \sqrt{\frac{[K^+]_o}{5.4}} \cdot xkr \cdot rkr \cdot (Vm - E_K)$$

$$I_{Kr\_SL} = F_{SL} \cdot G_{Kr} \cdot \sqrt{\frac{[K^+]_o}{5.4}} \cdot xkr \cdot rkr \cdot (Vm - E_K)$$

$$I_{Kr} = I_{Kr\_JS} + I_{Kr\_SL}$$

I_{Ks}; Slowly activating K^+ current

	Abbreviation	Value	Unit
conductance	$G_{Ks,0}$	0.0035	$nS \cdot pF^{-1}$
	pNaK	0.01833	

$$xks_{ss} = \frac{1}{1 + \exp\left(-\frac{Vm + 3.8 + Vshift}{14.25}\right)}$$

$$\tau_{xks} = \frac{990.1}{1 + \exp\left(-\frac{Vm + 2.436 + Vshift}{14.12}\right)}$$

$$\frac{dxks}{dt} = \frac{xks_{ss} - xks}{\tau_{xks}}$$

$$I_{Ks\_JS} = F_{JS} \cdot G_{Ks} \cdot xks^2 \cdot (Vm - E_{Ks\_JS})$$

$$I_{Ks\_SL} = F_{SL} \cdot G_{Ks} \cdot xks^2 \cdot (Vm - E_{Ks\_SL})$$

$$I_{Ks} = I_{Ks\_JS} + I_{Ks\_SL}$$

For β-adrenergic stimulation

$AI_{Ks} = 1 + \alpha \cdot 11$, where $\alpha = 1.0$ for standard β − adrenergic stimulation

$$G_{Ks} = AI_{Ks} \cdot G_{Ks,0}$$

$$Vshift = \alpha \cdot 5.0$$

I_{Kp}; Plateau K^+ current

	Abbreviation	Value	Unit
conductance	G_{Kp}	0.002	$nS \cdot pF^{-1}$

$$kp_{kp} = \frac{1}{1 + \exp\left(7.488 - \frac{Vm}{5.98}\right)}$$

$$I_{Kp\_JS} = F_{JS} \cdot G_{Kp} \cdot kp_{kp} \cdot (Vm - E_K)$$

$$I_{Kp\_SL} = F_{SL} \cdot G_{Kp} \cdot kp_{kp} \cdot (Vm - E_K)$$

$I_{Kp} = I_{Kp\_JS} + I_{Kp\_SL}$

$I_{to,f}$; Transient outward K^+ current, fast component

	Abbreviation	Value	Unit
conductance	$G_{to,f}$	0.1144	$nS \cdot pF^{-1}$

$$xto_{ss} = \frac{1}{1 + \exp\left(-\frac{Vm - 19.0}{13}\right)}$$

$$\tau_{xto,f} = 0.5 + 8.5 \cdot \exp\left(-\left(\frac{Vm + 45}{50}\right)^2\right)$$

$$yto_{ss} = \frac{1}{1 + \exp\left(\frac{Vm + 19.5}{5}\right)}$$

$$\tau_{yto,f} = 7.0 + 85 \cdot \exp\left(-\left(\frac{Vm + 40}{220}\right)^2\right)$$

$$\frac{dxto,f}{dt} = \frac{xto_{ss} - xto,f}{\tau_{xto,f}}$$

$$\frac{dyto,f}{dt} = \frac{yto_{ss} - yto,f}{\tau_{yto,f}}$$

$$I_{to,f\_JS} = F_{JS} \cdot G_{to,f} \cdot xto,f \cdot yto,f \cdot (Vm - E_K)$$

$$I_{to,f\_SL} = F_{SL} \cdot G_{to,f} \cdot xto,f \cdot yto,f \cdot (Vm - E_K)$$

$$I_{to,f} = I_{to,f\_JS} + I_{to,f\_SL}$$

$I_{to,s}$; Transient outward K^+ current, slow component

	Abbreviation	Value	Unit
conductance	$G_{to,s}$	0.0156	$nS \cdot pF^{-1}$

$$xto_{ss} = \frac{1}{1 + \exp\left(-\frac{Vm - 19.0}{13}\right)}$$

$$\tau_{xto,s} = 0.5 + \frac{9}{1 + \exp\left(\frac{Vm + 3}{15}\right)}$$

$$yto_{ss} = \frac{1}{1 + \exp\left(\frac{Vm + 19.5}{5}\right)}$$

$$\tau_{yto,s} = 30 + \frac{800}{1 + \exp\left(\frac{Vm + 60.0}{10}\right)}$$

$$\frac{dxto,s}{dt} = \frac{xto_{ss} - xto,s}{\tau_{xto,s}}$$

$$\frac{dyto,s}{dt} = \frac{yto_{ss} - yto,s}{\tau_{yto,s}}$$

$$I_{to,s\_JS} = F_{JS} \cdot G_{to,s} \cdot xto,s \cdot yto,s \cdot (Vm - E_K)$$
$$I_{to,s\_SL} = F_{SL} \cdot G_{to,s} \cdot xto,s \cdot yto,s \cdot (Vm - E_K)$$
$$I_{to,s} = I_{to,s\_JS} + I_{to,s\_SL}$$

I_{K1}; Inward rectifier K^+ current

	Abbreviation	Value	Unit
conductance	G_{K1}	0.35	$nS \cdot pF^{-1}$

$$\alpha_{K1} = \frac{1.02}{1 + \exp\left(0.2385 \cdot (Vm - E_K - 59.215)\right)}$$
$$\beta_{K1} = \frac{0.49124 \cdot \exp\left(0.08032 \cdot (Vm + 5.476 - E_K)\right) + \exp\left(0.06175 \cdot (Vm - 594.31 - E_K)\right)}{1 + \exp\left(-0.5143 \cdot (Vm + 4.753 - E_K)\right)}$$
$$K1_{ss} = \frac{\alpha_{K1}}{\alpha_{K1} + \beta_{K1}}$$
$$I_{K1\_JS} = F_{JS} \cdot G_{K1} \cdot \sqrt{\frac{[K^+]_o}{5.4}} \cdot K1_{ss} \cdot (Vm - E_K)$$
$$I_{K1\_SL} = F_{SL} \cdot G_{K1} \cdot \sqrt{\frac{[K^+]_o}{5.4}} \cdot K1_{ss} \cdot (Vm - E_K)$$
$$I_{K1} = I_{K1\_JS} + I_{K1\_SL}$$

I_{ClCa}; Ca^{2+} activated Cl^- current

	Abbreviation	Value	Unit
conductance	G_{ClCa}	0.0548125	$nS \cdot pF^{-1}$
	Kd_{ClCa}	0.1	mM

$$I_{ClCa\_JS} = \frac{F_{JS} \cdot G_{ClCa} \cdot (Vm - E_{Cl})}{1 + \frac{Kd_{ClCa}}{[Ca^{2+}]_{JS}}}$$
$$I_{ClCa\_SL} = \frac{F_{SL} \cdot G_{ClCa} \cdot (Vm - E_{Cl})}{1 + \frac{Kd_{ClCa}}{[Ca^{2+}]_{SL}}}$$
$$I_{ClCa} = I_{ClCa\_JS} + I_{ClCa\_SL}$$

I_{Clb}; Background Cl^- current

	Abbreviation	Value	Unit
conductance	G_{Clb}	0.009	$nS \cdot pF^{-1}$

$$I_{Clb\_JS} = F_{JS} \cdot G_{Clb} \cdot (Vm - E_{Cl})$$
$$I_{Clb\_SL} = F_{SL} \cdot G_{Clb} \cdot (Vm - E_{Cl})$$
$$I_{Clb} = I_{Clb\_JS} + I_{Clb\_SL}$$

I_{CaL}; L-type Ca^{2+} current

	Abbreviation	Value	Unit
permeability	$P_{Ca,0}$	0.1215	$cm \cdot sec^{-1}$
permeability	$P_{Na,0}$	$3.375 \cdot 10^{-6}$	$cm \cdot sec^{-1}$
permeability	$P_{K,0}$	$6.075 \cdot 10^{-5}$	$cm \cdot sec^{-1}$

$$d_{ss} = \frac{1}{1 + \exp\left(-\frac{Vm + 5}{6.0}\right)}$$
$$\tau_d = d_{ss} \cdot \frac{1 - \exp\left(-\frac{Vm + 5}{6.0}\right)}{0.035 \cdot (Vm + 5)}$$
$$f_{ss} = \frac{1}{1 + \exp\left(\frac{Vm + 35}{9}\right)} + \frac{0.6}{1 + \exp\left(\frac{50 - Vm}{20}\right)}$$
$$\tau_f = \frac{1}{0.0197 \cdot \exp\left(-\left(0.0337 \cdot (Vm + 14.5)\right)^2\right) + 0.02}$$
$$\frac{dd}{dt} = \frac{d_{ss} - d}{\tau_d}$$
$$\frac{df}{dt} = \frac{f_{ss} - f}{\tau_f}$$
$$\frac{dfCaB_{JS}}{dt} = 1.7 \cdot [Ca^{2+}]_{JS} \cdot \left(1 - fCaB_{JS}\right) - (11.9 \cdot 10^{-3}) \cdot fCaB_{JS}$$
$$\frac{dfCaB_{SL}}{dt} = 1.7 \cdot [Ca^{2+}]_{SL} \cdot (1 - fCaB_{SL}) - (11.9 \cdot 10^{-3}) \cdot fCaB_{SL}$$
$$I_{CaL\_CaJS} = F_{JSCa} \cdot d \cdot f \cdot \left(1 - fCaB_{JS}\right) \cdot \frac{P_{Ca} \cdot 4 \cdot Vm \cdot F \cdot \frac{F}{RT} \cdot \left(0.341 \cdot [Ca^{2+}]_{JS} \cdot \exp\left(2 \cdot Vm \cdot \frac{F}{RT}\right) - 0.341 \cdot [Ca^{2+}]_o\right)}{\exp\left(2 \cdot Vm \cdot \frac{F}{RT}\right) - 1}$$
$$I_{CaL\_CaSL} = F_{SLCa} \cdot d \cdot f \cdot (1 - fCaB_{SL}) \cdot \frac{P_{Ca} \cdot 4 \cdot Vm \cdot F \cdot \frac{F}{RT} \cdot \left(0.341 \cdot [Ca^{2+}]_{SL} \cdot \exp\left(2 \cdot Vm \cdot \frac{F}{RT}\right) - 0.341 \cdot [Ca^{2+}]_o\right)}{\exp\left(2 \cdot Vm \cdot \frac{F}{RT}\right) - 1}$$
$$I_{CaL\_Ca} = I_{CaL\_CaJS} + I_{CaL\_CaSL}$$
$$I_{CaL\_NaJS} = F_{JSCa} \cdot d \cdot f \cdot \left(1 - fCaB_{JS}\right) \cdot \frac{P_{Na} \cdot Vm \cdot F \cdot \frac{F}{RT} \cdot \left(0.75 \cdot [Na^{+}]_{JS} \cdot \exp\left(Vm \cdot \frac{F}{RT}\right) - 0.75 \cdot [Na^{+}]_o\right)}{\exp\left(Vm \cdot \frac{F}{RT}\right) - 1}$$

$$I_{CaL\_NaSL} = F_{SLCa} \cdot d \cdot f \cdot (1 - fCaB_{SL}) \cdot \frac{P_{Na} \cdot Vm \cdot F \cdot \frac{F}{RT} \cdot \left(0.75 \cdot [Na^+]_{SL} \cdot \exp\left(Vm \cdot \frac{F}{RT}\right) - 0.75 \cdot [Na^+]_o\right)}{\exp\left(Vm \cdot \frac{F}{RT}\right) - 1}$$

$$I_{CaL\_Na} = I_{CaL\_NaJS} + I_{CaL\_NaSL}$$

$$I_{CaL\_KJS} = F_{JSCa} \cdot d \cdot f \cdot \left(1 - fCaB_{JS}\right) \cdot \frac{P_K \cdot Vm \cdot F \cdot \frac{F}{RT} \cdot \left(0.75 \cdot [K^+]_{JS} \cdot \exp\left(Vm \cdot \frac{F}{RT}\right) - 0.75 \cdot [K^+]_o\right)}{\exp\left(Vm \cdot \frac{F}{RT}\right) - 1}$$

$$I_{CaL\_KSL} = F_{SLCa} \cdot d \cdot f \cdot (1 - fCaB_{SL}) \cdot \frac{P_K \cdot Vm \cdot F \cdot \frac{F}{RT} \cdot \left(0.75 \cdot [K^+]_{SL} \cdot \exp\left(Vm \cdot \frac{F}{RT}\right) - 0.75 \cdot [K^+]_o\right)}{\exp\left(Vm \cdot \frac{F}{RT}\right) - 1}$$

$$I_{CaL\_K} = I_{CaL\_KJS} + I_{CaL\_KSL}$$

$$I_{CaL} = I_{CaL\_Ca} + I_{CaL\_Na} + I_{CaL\_K}$$

For β-adrenergic stimulation

$AI_{CaL} = 1 + \alpha \cdot 0.3$, where $\alpha = 1.0$ for standard $\beta -$ adrenergic stimulation

$$P_{Ca} = AI_{CaL} \cdot P_{Ca,0}$$

$$P_{Na} = AI_{CaL} \cdot P_{Na,0}$$

$$P_K = AI_{CaL} \cdot P_{K,0}$$

I_{pCa}; Sarcolemmal Ca^{2+} pump current

	Abbreviation	Value	Unit
maximum velocity	V_{max_pCa}	0.0673	$pA \cdot pF^{-1}$
dissociation constant	Km_{pCa}	$5.0 \cdot 10^{-4}$	mM

$$I_{pCa\_JS} = \frac{F_{JS} \cdot V_{max\_pCa} \cdot [Ca^{2+}]_{JS}{}^{1.6}}{Km_{pCa}^{1.6} + [Ca^{2+}]_{JS}{}^{1.6}}$$

$$I_{pCa\_SL} = \frac{F_{SL} \cdot V_{max\_pCa} \cdot [Ca^{2+}]_{SL}{}^{1.6}}{Km_{pCa}^{1.6} + [Ca^{2+}]_{SL}{}^{1.6}}$$

$$I_{pCa} = I_{pCa\_JS} + I_{pCa\_SL}$$

ATP consumption rate in $mM \cdot msec^{-1}$

$$\frac{dATPuse\_I_{pCa}}{dt} = \frac{I_{pCa} \cdot Cm}{(Vol_{cyt} + Vol_{JS} + Vol_{SL}) \cdot F}$$

I_{Cab}; Background Ca^{2+} current

	Abbreviation	Value	Unit

conductance	G_{Cab}	$5.513\cdot 10^{-4}$	$nS\cdot pF^{-1}$

$$I_{Cab\_JS} = F_{JS}\cdot G_{Cab}\cdot (Vm - E_{Ca\_JS})$$

$$I_{Cab\_SL} = F_{SL}\cdot G_{Cab}\cdot (Vm - E_{Ca\_SL})$$

$$I_{Cab} = I_{Cab\_JS} + I_{Cab\_SL}$$

I_{NCX}; Sarcolemmal Na^{+}-Ca^{2+} exchange current

	Abbreviation	Value	Unit
maximum velocity	V_{max_NCX}	4.5	$pA\cdot pF^{-1}$
dissociation constant	Kd_{act}	$1.5\cdot 10^{-4}$	mM
dissociation constant	Km_{Cai}	0.00359	mM
dissociation constant	Km_{Cao}	1.3	mM
dissociation constant	Km_{Nai}	12.29	mM
dissociation constant	Km_{Nao}	87.5	mM
	nu	0.27	
	k_{sat}	0.32	

$$Ka_{JS} = \frac{1}{1+\left(\frac{Kd_{act}}{[Ca^{2+}]_{JS}}\right)^{2}}$$

$$Ka_{SL} = \frac{1}{1+\left(\frac{Kd_{act}}{[Ca^{2+}]_{SL}}\right)^{2}}$$

$$s1_{JS} = \exp\left(nu\cdot Vm\cdot\frac{F}{RT}\right)\cdot [Na^{+}]_{JS}{}^{3}\cdot [Ca^{2+}]_{o}$$

$$s1_{SL} = \exp\left(nu\cdot Vm\cdot\frac{F}{RT}\right)\cdot [Na^{+}]_{SL}{}^{3}\cdot [Ca^{2+}]_{o}$$

$$s2_{JS} = \exp\left((nu-1)\cdot Vm\cdot\frac{F}{RT}\right)\cdot [Na^{+}]_{o}{}^{3}\cdot [Ca^{2+}]_{JS}$$

$$s2_{SL} = \exp\left((nu-1)\cdot Vm\cdot\frac{F}{RT}\right)\cdot [Na^{+}]_{o}{}^{3}\cdot [Ca^{2+}]_{SL}$$

$$s3_{JS} = Km_{Ca_i}\cdot [Na^{+}]_{o}{}^{3}\cdot\left(1+\left(\frac{[Na^{+}]_{JS}}{Km_{Na_i}}\right)^{3}\right) + Km_{Na_o}^{3}\cdot [Ca^{2+}]_{JS}\cdot\left(1+\frac{[Ca^{2+}]_{JS}}{Km_{Ca_i}}\right) + Km_{Ca_o}\cdot [Na^{+}]_{JS}{}^{3} + [Na^{+}]_{JS}{}^{3}\cdot [Ca^{2+}]_{o} + [Na^{+}]_{o}{}^{3}\cdot [Ca^{2+}]_{JS}$$

$$s3_{SL} = Km_{Ca_i}\cdot [Na^{+}]_{o}{}^{3}\cdot\left(1+\left(\frac{[Na^{+}]_{SL}}{Km_{Na_i}}\right)^{3}\right) + Km_{Na_o}^{3}\cdot [Ca^{2+}]_{SL}\cdot\left(1+\frac{Ca_{SL}}{Km_{Ca_i}}\right) + Km_{Ca_o}\cdot [Na^{+}]_{SL}{}^{3} + [Na^{+}]_{SL}{}^{3}\cdot [Ca^{2+}]_{o} + [Na^{+}]_{o}{}^{3}\cdot [Ca^{2+}]_{SL}$$

$$I_{NCX\_JS} = \frac{F_{JS}\cdot V_{max\_NCX}\cdot Ka_{JS}\cdot (s1_{JS} - s2_{JS})}{s3_{JS}\cdot\left(1+k_{sat}\cdot\exp\left((nu-1)\cdot Vm\cdot\frac{F}{RT}\right)\right)}$$

$$I_{NCX\_SL} = \frac{F_{SL} \cdot V_{max\_NCX} \cdot Ka_{SL} \cdot (s1_{SL} - s2_{SL})}{s3_{SL} \cdot \left(1 + k_{sat} \cdot \exp\left((nu - 1) \cdot Vm \cdot \frac{F}{RT}\right)\right)}$$ $$I_{NCX} = I_{NCX\_JS} + I_{NCX\_SL}$$

Table S4. Ion diffusions between compartments

$J_{Ca\_cyt\_SL}$; Ca^{2+} diffusion between cytoplasmic space and subsarcolemmal space

	Abbreviation	Value	Unit
permeability	PCa_{cyt_SL}	4096.73	$\mu m^3 \cdot msec^{-1}$
$J_{Ca\_cyt\_SL} = PCa_{cyt\_SL} \cdot ([Ca^{2+}]_{SL} - [Ca^{2+}]_{cyt})$			

$J_{Ca\_JS\_SL}$; Ca^{2+} diffusion between junctional space and subsalcolemmal space

	Abbreviation	Value	Unit
permeability	PCa_{JS_SL}	906.543	$\mu m^3 \cdot msec^{-1}$
$J_{Ca\_JS\_SL} = PCa_{JS\_SL} \cdot ([Ca^{2+}]_{SL} - [Ca^{2+}]_{JS})$			

$J_{Na\_cyt\_SL}$; Na^{+} diffusion between cytoplasmic space and subsarcolemmal space

	Abbreviation	Value	Unit
permeability	PNa_{cyt_SL}	1638.63	$\mu m^3 \cdot msec^{-1}$
$J_{Na\_cyt\_SL} = PNa_{cyt\_SL} \cdot ([Na^{+}]_{SL} - [Na^{+}]_{cyt})$			

$J_{Na\_JS\_SL}$; Na^{+} diffusion between junctional space and subsalcolemmal space

	Abbreviation	Value	Unit
permeability	PNa_{JS_SL}	18.3128	$\mu m^3 \cdot msec^{-1}$
$J_{Na\_JS\_SL} = PNa_{JS\_SL} \cdot ([Na^{+}]_{SL} - [Na^{+}]_{JS})$			

Table S5. Junctional space

J_{CaB_JS}; Junctional space Ca^{2+} buffers

	Abbreviation	Value	Unit
association constant	kon_{JSlow}	100	$mM^{-1} \cdot msec^{-1}$
dissociation constant	$koff_{JSlow}$	1.3	$msec^{-1}$
total low affinity Ca^{2+} buffer in JS ($CaB_{\_JS\_low}$) concentration	$Bmax_{JSlow}$	$(4.6 \cdot 10^{-4}) \cdot \frac{Vol_{cyt}}{Vol_{JS}}$	mM
association constant	kon_{JShigh}	100	$mM^{-1} \cdot msec^{-1}$
dissociation constant	$koff_{JShigh}$	0.03	$msec^{-1}$
total high affinity Ca^{2+} buffer in JS ($CaB_{\_JS\_high}$) concentration	$Bmax_{JShigh}$	$(1.65 \cdot 10^{-4}) \cdot \frac{Vol_{cyt}}{Vol_{JS}}$	mM

$$\frac{d[CaB_{JS\_low}]}{dt} = kon_{JSlow} \cdot [Ca^{2+}]_{JS} \cdot (Bmax_{JSlow} - [CaB_{JS\_low}]) - koff_{JSlow} \cdot [CaB_{JS\_low}]$$ $$\frac{d[CaB_{JS\_high}]}{dt} = kon_{JShigh} \cdot [Ca^{2+}]_{JS} \cdot (Bmax_{JShigh} - [CaB_{JS\_high}]) - koff_{JShigh} \cdot [CaB_{JS\_high}]$$ $$J_{CaB\_JS} = \frac{d[CaB_{JS\_low}]}{dt} + \frac{d[CaB_{JS\_high}]}{dt}$$

J_{NaB_JS}; Junctional space Na^+ buffer

	Abbreviation	Value	Unit
association constant	kon_{JS}	$1.0 \cdot 10^{-4}$	$mM^{-1} \cdot msec^{-1}$
dissociation constant	$koff_{JS}$	$1.0 \cdot 10^{-3}$	$msec^{-1}$
total Na^+ buffer in JS ($NaB_{\_JS}$) concentration	$Bmax_{JS}$	7.561	mM
$$J_{NaB\_JS} = \frac{d[NaB_{JS}]}{dt} = kon_{JS} \cdot [Na^+]_{JS} \cdot (Bmax_{JS} - [NaB_{JS}]) - koff_{JS} \cdot [NaB_{JS}]$$			

Table S6. Subsarcolemmal space

J_{CaB_SL}; Subsarcolemmal space Ca^{2+} buffers

	Abbreviation	Value	Unit
association constant	kon_{SLlow}	100	$mM^{-1} \cdot msec^{-1}$
dissociation constant	$koff_{SLlow}$	1.3	$msec^{-1}$
total low affinity Ca^{2+} buffer in SL ($CaB_{\_SL\_low}$) concentration	$Bmax_{SLlow}$	$0.0374 \cdot \frac{Vol_{cyt}}{Vol_{SL}}$	mM
association constant	kon_{SLhigh}	100	$mM^{-1} \cdot msec^{-1}$
dissociation constant	$koff_{SLhigh}$	0.03	$msec^{-1}$
total high affinity Ca^{2+} buffer in SL ($CaB_{\_SL\_high}$) concentration	$Bmax_{SLhigh}$	$0.0134 \cdot \frac{Vol_{cyt}}{Vol_{SL}}$	mM
$$\frac{d[CaB_{SL\_low}]}{dt} = kon_{SLlow} \cdot [Ca^{2+}]_{SL} \cdot (Bmax_{SLlow} - [CaB_{SL\_low}]) - koff_{SLlow} \cdot [CaB_{SL\_low}]$$ $$\frac{d[CaB_{SL\_high}]}{dt} = kon_{SLhigh} \cdot [Ca^{2+}]_{SL} \cdot (Bmax_{SLhigh} - [CaB_{SL\_high}]) - koff_{SLhigh} \cdot [CaB_{SL\_high}]$$ $$J_{CaB\_SL} = \frac{d[CaB_{SL\_low}]}{dt} + \frac{d[CaB_{SL\_high}]}{dt}$$			

J_{NaB_SL}; Subsarcolemmal space Na^+ buffer

	Abbreviation	Value	Unit
association constant	kon_{SL}	$1.0 \cdot 10^{-4}$	$mM^{-1} \cdot msec^{-1}$
dissociation constant	$koff_{SL}$	$1.0 \cdot 10^{-3}$	$msec^{-1}$

total Na^+ buffer in SL ($NaB_{\_SL}$) concentration n	$Bmax_{SL}$	1.65	mM

$$J_{NaB\_SL} = \frac{d[NaB_{SL}]}{dt} = kon_{SL} \cdot [Na^+]_{SL} \cdot (Bmax_{SL} - [NaB_{SL}]) - koff_{SL} \cdot [NaB_{SL}]$$

Table S7. Cytoplasmic space

Contraction

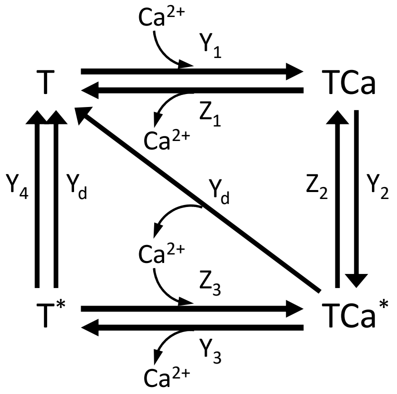

	Abbreviation	Value	Unit
adjustment factor	a_{cm}	12.8	$mM^{-1} \cdot msec^{-1}$
adjustment factor	b_{cm}	0.054	$msec^{-1}$
adjustment factor	f_{cm}	0.0000851	$msec^{-1}$
adjustment factor	g_{cm}	0.000649	$msec^{-1}$
rate constant	B_{eff}	−0.001887	$\mu m \cdot msec^{-1}$
equilibrium length of the cross bridge	hc	0.005	μm
	A_{Fb}	380000	$mN \cdot mm^{-2} \cdot \mu m^{-1} \cdot mM^{-1}$
	K_{PE}	3.0	$mN \cdot mm^{-2}$
	K_{PL}	30.0	$mN \cdot mm^{-2}$
	D	10.0	
	L_0	0.965	μm
total troponin concentration	[totalTroponin]	0.05	mM

$$Y_1 = a_{cm} \cdot [Ca^{2+}]_{cyt}{}^{0.81} \ (msec^{-1})$$

$$Z_1 = b_{cm} \cdot (1 + f_{23} \cdot (\exp(-2.09) - 1))^2 \cdot (1 + f_3 \cdot (\exp 0.73 - 1))^2 (msec^{-1})$$

$$Y_2 = f_{cm} \cdot (1 + f_{23} \cdot (\exp 2.96 - 1))^2 \cdot (1 + f_3 \cdot (\exp(-2.1) - 1))^2 (msec^{-1})$$

$$Z_2 = g_{cm} \cdot (1 + f_3 \cdot (\exp(-0.26) - 1))^2 (msec^{-1})$$

$$Y_3 = Z_1 (msec^{-1})$$

$$Z_3 = 40 \cdot Y_1 \ (msec^{-1})$$

$$Y_4 = 0.24 \ (msec^{-1})$$

$$Y_d = \begin{cases} 180 \cdot \left(\frac{dX}{dt}\right)^2, \frac{dX}{dt} > 0 \\ 9000 \cdot \left(\frac{dX}{dt}\right)^2, \text{otherwise} \end{cases} \quad (\text{msec} \cdot \mu\text{m}^{-2})$$

$$f_{23} = \frac{1}{\left(1 + \exp\left(\frac{hsmL - 0.85}{-0.08}\right)\right) \cdot \left(1 + \exp\left(\frac{hsmL - 1.4}{0.06}\right)\right)} \cdot \frac{[TCa] + [TCa^*] + [T^*]}{[totalTroponin]}$$

$$f_3 = \frac{1}{\left(1 + \exp\left(\frac{hsmL - 0.85}{-0.08}\right)\right) \cdot \left(1 + \exp\left(\frac{hsmL - 1.4}{0.06}\right)\right)} \cdot \frac{[TCa^*] + [T^*]}{[totalTroponin]}$$

$$F_b = A_{Fb} \cdot \frac{1}{\left(1 + \exp\left(\frac{hsmL - 0.85}{-0.08}\right)\right) \cdot \left(1 + \exp\left(\frac{hsmL - 1.4}{0.06}\right)\right)} \cdot ([TCa^*] + [T^*]) \cdot (hsmL - hsmX)$$

$$F_p = \begin{cases} K_{PE} \cdot \left(\exp\left(D \cdot \left(\frac{hsmL}{L_0} - 1\right)\right) - 1\right), hsmL \geq L_0 \\ -K_{PL} \cdot \left(1 - \frac{hsmL}{L_0}\right), \text{otherwise} \end{cases}$$

$$\frac{dhsmX}{dt} = B_{eff} \cdot \left(\exp\left(\frac{hc - hsmL + hsmX}{0.00225}\right) - 1\right)$$

$$\frac{dhsmL}{dt} = \frac{F_{ext} - (F_b + F_p)}{10.0}$$

$$\frac{d[TCa]}{dt} = Y_1 \cdot [T] + Z_2 \cdot [TCa^*] - (Z_1 + Y_2) \cdot [TCa]$$

$$\frac{d[TCa^*]}{dt} = Y_2 \cdot [TCa] + Z_3 \cdot [T^*] - (Z_2 + Y_3 + Y_d) \cdot [TCa^*]$$

$$\frac{d[T^*]}{dt} = Y_3 \cdot [TCa^*] - (Z_3 + Y_4 + Y_d) \cdot [T^*]$$

$$[T] = [totalTroponin] - ([TCa] + [TCa^*] + [T^*])$$

$$J_{Ca\_troponin} = Z_1 \cdot [TCa] - Y_1 \cdot [T] + Y_d \cdot [TCa^*] + Y_3 \cdot [TCa^*] - Z_3 \cdot [T^*]$$

ATP consumption rate in mM· msec^{-1}

$$\frac{dATPuse\_contraction}{dt} = (2.4 \cdot 10^{-5}) \cdot A_{Fb} \cdot (Z_2 \cdot [TCa^*] + Y_4 \cdot [T^*])$$

J_{CaB_total}; Cytoplasmic Ca^{2+} buffers

	Abbreviation	Value	Unit
association constant	kon_{TnChCa}	2.37	$mM^{-1} \cdot msec^{-1}$
dissociation constant	$koff_{TnChCa}$	$3.2 \cdot 10^{-5}$	$msec^{-1}$
association constant	kon_{TnChMg}	0.003	$mM^{-1} \cdot msec^{-1}$

dissociation constant	$koff_{TnChMg}$	0.00333	$msec^{-1}$
total TnC_{high} concentration	$Bmax_{TnChigh}$	0.14	mM
association constant	kon_{CaM}	34.0	$mM^{-1} \cdot msec^{-1}$
dissociation constant	$koff_{CaM}$	0.238	$msec^{-1}$
total CaM concentration	$Bmax_{CaM}$	0.024	mM
association constant	$kon_{myocinCa}$	13.8	$mM^{-1} \cdot msec^{-1}$
dissociation constant	$koff_{myocinCa}$	$4.6 \cdot 10^{-4}$	$msec^{-1}$
association constant	$kon_{myocinMg}$	0.0157	$mM^{-1} \cdot msec^{-1}$
dissociation constant	$koff_{myocinMg}$	$5.7 \cdot 10^{-5}$	$msec^{-1}$
total myocin concentration	$Bmax_{myocin}$	0.14	mM
association constant	kon_{SRB}	100	$mM^{-1} \cdot msec^{-1}$
dissociation constant	$koff_{SRB}$	0.06	$msec^{-1}$
total SRB concentration	$Bmax_{SRB}$	0.0171	mM

$$\frac{d[TnC_{hCa}]}{dt} = kon_{TnChCa} \cdot [Ca^{2+}]_{cyt} \cdot \left(Bmax_{TnChigh} - [TnC_{hCa}] - [TnC_{hMg}]\right) - koff_{TnChCa} \cdot [TnC_{hCa}]$$

$$\frac{d[TnC_{hMg}]}{dt} = kon_{TnChMg} \cdot [Mg^{2+}]_{cyt} \cdot \left(Bmax_{TnChigh} - [TnC_{hCa}] - [TnC_{hMg}]\right) - koff_{TnChMg} \cdot [TnC_{hMg}]$$

$$\frac{d[CaM]}{dt} = kon_{CaM} \cdot [Ca^{2+}]_{cyt} \cdot (Bmax_{CaM} - [CaM]) - koff_{CaM} \cdot [CaM]$$

$$\frac{d[Myocin_{Ca}]}{dt} = kon_{myocinCa} \cdot [Ca^{2+}]_{cyt} \cdot \left(Bmax_{myocin} - [Myocin_{Ca}] - [Myocin_{Mg}]\right) - koff_{myocinCa} \cdot [Myocin_{Ca}]$$

$$\frac{d[Myocin_{Mg}]}{dt} = kon_{myocinMg} \cdot [Mg^{2+}]_{cyt} \cdot \left(Bmax_{myocin} - [Myocin_{Ca}] - [Myocin_{Mg}]\right) - koff_{myocinMg} \cdot [Myocin_{Mg}]$$

$$\frac{d[SRB]}{dt} = kon_{SRB} \cdot [Ca^{2+}]_{cyt} \cdot (Bmax_{SRB} - [SRB]) - koff_{SRB} \cdot [SRB]$$

$$J_{CaB\_total} = \frac{d[TnC_{hCa}]}{dt} + \frac{d[TnC_{hMg}]}{dt} + \frac{d[CaM]}{dt} + \frac{d[Myocin_{Ca}]}{dt} + \frac{d[Myocin_{Mg}]}{dt} + \frac{d[SRB]}{dt}$$

J_{AK}; Adenylate kinase (AK) flux

	Abbreviation	Value	Unit
association constant	$k_{AK,f}$	49.283	$mM^{-1} \cdot msec^{-1}$
dissociation constant	$k_{AK,b}$	1.30035	$mM^{-1} \cdot msec^{-1}$

$$J_{AK} = k_{AK,f} \cdot [freeADP]_{cyt} \cdot [MgADP]_{cyt} - k_{AK,b} \cdot [MgATP]_{cyt} \cdot [AMP]_{cyt}$$

J_{CK}; Creatine kinase (CK) flux

	Abbreviation	Value	Unit
association constant	$k_{CK,f}$	0.01101	$mM^{-1}\cdot msec^{-1}$
dissociation constant	$k_{CK,b}$	$5.0042\cdot 10^{-5}$	$mM^{-1}\cdot msec^{-1}$
$J_{CK} = k_{CK,f}\cdot[totalADP]_{cyt}\cdot[PCr]_{cyt} - k_{CK,b}\cdot[totalATP]_{cyt}\cdot[Cr]_{cyt}$			

Table S8. SR Ca^{2+} fluxes

J_{SERCA}; SR Ca^{2+} pump SERCA flux

	Abbreviation	Value	Unit
maximum velocity	$Vmax_{\_SERCA,0}$	0.011382	$mM\cdot msec^{-1}$
dissociation constant	Km_f	$2.46\cdot 10^{-4}$	mM
dissociation constant	Km_r	1.7	mM
fraction of SERCA facing cytoplasm	fSERCA		

$$J_{SERCA} = fSERCA\cdot V_{max\ \_SERCA}\cdot\frac{\left(\left(\frac{[Ca^{2+}]_{cyt}}{Km_f}\right)^{1.787}-\left(\frac{[Ca^{2+}]_{SR}}{Km_r}\right)^{1.787}\right)}{1+\left(\frac{[Ca^{2+}]_{cyt}}{Km_f}\right)^{1.787}+\left(\frac{[Ca^{2+}]_{SR}}{Km_r}\right)^{1.787}}$$

For MSI model

$$fSERCA = 0.7\left(= 1.0 - fNmSC\cdot\frac{0.3}{0.7}, \text{where } fNmSC = 0.7\right)$$

For non-MSI model

$$fSERCA = 1.0\left(= 1.0 - fNmSC\cdot\frac{0.3}{0.7}, \text{where } fNmSC = 0.0\right)$$

For β-adrenergic stimulation

$ASERCA = 1 + \alpha\cdot 0.3$, where $\alpha = 1.0$ for standard β − adrenergic stimulation

$V_{max\_SERCA} = ASERCA\cdot V_{max\ \_SERCA,0}$

ATP consumption rate in mM· msec^{-1}

$$\frac{dATPuse\_SERCA}{dt} = \frac{J_{SERCA}\cdot Vol_{SR}}{2\cdot(Vol_{cyt}+Vol_{JS}+Vol_{SL})}$$

J_{RyR}; RyR channel Ca^{2+} flux

	Abbreviation	Value	Unit
	ks	37.5	$msec^{-1}$
	$ec50_{SR}$	0.45	mM
	ko_{Ca}	10.0	$mM^{2}\cdot msec^{-1}$

	ko_m	0.06	$msec^{-1}$
	ki_{Ca}	0.5	$mM^{-1} \cdot msec^{-1}$
	ki_m	0.005	$msec^{-1}$

$$Max_{SR} = 15$$

$$Min_{SR} = 1$$

$$k_{Ca_{SR}} = Max_{SR} - \frac{Max_{SR} - Min_{SR}}{1 + \left(\frac{ec_{50_{SR}}}{[Ca^{2+}]_{SR}}\right)^{2.5}}$$

$$ko_{SR_{Ca}} = \frac{ko_{Ca}}{k_{Ca_{SR}}}$$

$$ki_{SR_{Ca}} = ki_{Ca} \cdot k_{Ca_{SR}}$$

$$RI = 1 - RyR_r - RyR_o - RyR_i$$

$$\frac{dRyR_r}{dt} = \left(ki_m \cdot RI - ki_{SR_{Ca}} \cdot [Ca^{2+}]_{JS} \cdot RyR_r\right) - \left(ko_{SR_{Ca}} \cdot {[Ca^{2+}]_{JS}}^2 \cdot RyR_r - ko_m \cdot RyR_o\right)$$

$$\frac{dRyR_o}{dt} = \left(ko_{SR_{Ca}} \cdot {[Ca^{2+}]_{JS}}^2 \cdot RyR_r - ko_m \cdot RyR_o\right) - \left(ki_{SR_{Ca}} \cdot [Ca^{2+}]_{JS} \cdot RyR_o - ki_m \cdot RyR_i\right)$$

$$\frac{dRyR_i}{dt} = \left(ki_{SR_{Ca}} \cdot [Ca^{2+}]_{JS} \cdot RyR_o - ki_m \cdot RyR_i\right) - \left(ko_m \cdot RyR_i - ko_{SR_{Ca}} \cdot {[Ca^{2+}]_{JS}}^2 \cdot RI\right)$$

$$J_{RyR} = k_s \cdot RyR_o \cdot \left([Ca^{2+}]_{SR} - [Ca^{2+}]_{JS}\right)$$

J_{leak}; SR Ca^{2+} leak flux

	Abbreviation	Value	Unit
maximum velocity	V_{max_leak}	$1.23004 \cdot 10^{-5}$	$msec^{-1}$

$$J_{leak} = V_{max\_leak} \cdot \left([Ca^{2+}]_{SR} - [Ca^{2+}]_{JS}\right)$$

J_{Csqn}; Ca^{2+} buffer in SR (Calsequestrin)

	Abbreviation	Value	Unit
association constant	kon_{csqn}	100	$mM^{-1} \cdot msec^{-1}$
dissociation constant	$koff_{csqn}$	65.0	$msec^{-1}$
total calsequestrin concentration	$Bmax_{csqn}$	$0.14 \cdot \frac{Vol_{cyt}}{Vol_{SR}}$	mM

$$J_{Csqn} = \frac{d[Csqn_b]}{dt} = kon_{csqn} \cdot [Ca^{2+}]_{SR} \cdot \left(Bmax_{csqn} - [Csqn_b]\right) - koff_{csqn} \cdot [Csqn_b]$$

Table S9. Mitochondrial ion/metabolite/enzyme fluxes

<u>Cation transporting system</u>

J_{CaUni}; Mitochondrial Ca^{2+} uniporter flux

	Abbreviation	Value	Unit

	P_{CaUni}	0.03681	$msec^{-1}$
	α_{mit}	0.2	
	α_i	0.341	
	Ki_{Camit}	0.01	mM
	K_{inh}	$5.0 \cdot 10^{-5}$	mM
	K_{rec}	$8.0 \cdot 10^{-4}$	mM
hill coefficient	n	2	
fraction of CaUni facing JS	fCaUni_js		

$$Ca_{mit\_inh} = \frac{{Ki_{Camit}}^{n}}{{[Ca^{2+}]_{mit}}^{n} + {Ki_{Camit}}^{n}}$$

$$Ca_{mit\_reg} = 0.9 - \left(\frac{[Ca^{2+}]_{mit}}{[Ca^{2+}]_{mit} + K_{inh}} - 0.3\right) \cdot \left(\frac{{K_{rec}}^{n}}{{[Ca^{2+}]_{mit}}^{n} + {K_{rec}}^{n}} - 0.3\right)$$

$$J_{CaUni\_JS} = fCaUni\_js \cdot P_{CaUni} \cdot Ca_{mit\_inh} \cdot Ca_{mit\_reg} \cdot \left(2 \cdot \Delta\psi \cdot \frac{F}{RT}\right) \cdot \left(\frac{\alpha_{mit} \cdot [Ca^{2+}]_{mit} - \alpha_i \cdot [Ca^{2+}]_{JS} \cdot \exp\left(-2 \cdot \Delta\psi \cdot \frac{F}{RT}\right)}{\exp\left(-2 \cdot \Delta\psi \cdot \frac{F}{RT}\right) - 1}\right)$$

$$J_{CaUni\_cyt} = (1 - fCaUni\_js) \cdot P_{CaUni} \cdot Ca_{mit\_inh} \cdot Ca_{mit\_reg} \cdot \left(2 \cdot \Delta\psi \cdot \frac{F}{RT}\right) \cdot \left(\frac{\alpha_{mit} \cdot [Ca^{2+}]_{mit} - \alpha_i \cdot [Ca^{2+}]_{cyt} \cdot \exp\left(-2 \cdot \Delta\psi \cdot \frac{F}{RT}\right)}{\exp\left(-2 \cdot \Delta\psi \cdot \frac{F}{RT}\right) - 1}\right)$$

For MSI model

fCaUni_js = 0.5

For non-MSI model

fCaUni_js = 0.0

J_{NmSC}; NCLX–SERCA complex flux & J_{NCXmit_cyt}; Mitochondrial Na^+-Ca^{2+} exchange flux

	Abbreviation	Value	Unit
	P_{NmSC}	$1.0616 \cdot 10^{-3}$	$mM \cdot msec^{-1}$
dissociation constants	Kd_{Namit}	18.7137	mM
	Kd_{Nai}	32.0	mM
	Kd_{Camit}	0.0025	mM
	Kd_{Cai}	0.0125	mM
hill coefficient	n	3	
fraction of NmSC	fNmSC		

$$PNa_{mit} = \frac{[Na^+]_{mit}{}^n}{[Na^+]_{mit}{}^n + Kd_{Namit}{}^n \cdot \left(1 + \frac{[Ca^{2+}]_{mit}}{Kd_{Camit}}\right)}$$

$$PCa_{mit} = \frac{[Ca^{2+}]_{mit}}{[Ca^{2+}]_{mit} + Kd_{Camit} \cdot \left(1 + \left(\frac{[Na^+]_{mit}}{Kd_{Namit}}\right)^n\right)}$$

$$PNa_i = \frac{[Na^+]_{cyt}{}^n}{[Na^+]_{cyt}{}^n + Kd_{Nai}{}^n \cdot \left(1 + \frac{[Ca^{2+}]_{cyt}}{Kd_{Cai}}\right)}$$

$$PCa_i = \frac{[Ca^{2+}]_{cyt}}{[Ca^{2+}]_{cyt} + Kd_{Cai} \cdot \left(1 + \left(\frac{[Na^+]_{cyt}}{Kd_{Nai}}\right)^n\right)}$$

$$k1 = 1.0 \cdot \exp\left(0.2 \cdot \Delta\psi \cdot \frac{F}{RT}\right) \cdot PNa_{mit}$$

$$k2 = 1.0 \cdot \exp\left((0.2 - 1.0) \cdot \Delta\psi \cdot \frac{F}{RT}\right) \cdot PNa_i$$

$$k3 = 1.0 \cdot PCa_{mit}$$

$$k4 = 1.0 \cdot PCa_i$$

$$\alpha = k2 + k4$$

$$\beta = k1 + k3$$

$$tE1 = \frac{\alpha}{\alpha + \beta}$$

$$tE2 = 1 - tE1$$

$$J_{NmSC} = -fNmSC \cdot P_{NmSC} \cdot (tE2 \cdot k2 - tE1 \cdot k1)$$

$$J_{NCXmit\_cyt} = -(1 - fNmSC) \cdot P_{NmSC} \cdot (tE2 \cdot k2 - tE1 \cdot k1)$$

For MSI model

fNmSC = 0.7

For non-MSI model

fNmSC = 0.0

ATP consumption rate by NmSC in mM· msec^{-1}

$$\frac{dATPuse\_NmSC}{dt} = \frac{-J_{NmSC} \cdot Vol_{mit}}{2 \cdot (Vol_{cyt} + Vol_{JS} + Vol_{SL})}$$

J_{KUni}; Mitochondrial K^+ uniporter flux

	Abbreviation	Value	Unit
	k_{KUni}	$1.45 \cdot 10^{-7}$	msec^{-1}

$$J_{KUni} = k_{KUni} \cdot \left([K^+]_{cyt} \cdot \exp\left(-\Delta\Psi \cdot \frac{F}{2 \cdot RT}\right) - [K^+]_{mit} \cdot \exp\left(\Delta\Psi \cdot \frac{F}{2 \cdot RT}\right)\right)$$

J_{KHE}; Mitochondrial K^+/H^+ exchange flux

	Abbreviation	Value	Unit
	k_{KHE}	0.150162	$msec^{-1}$
$J_{KHE} = k_{KHE} \cdot ([K^+]_{mit} \cdot [H^+]_{cyt} - [K^+]_{cyt} \cdot [H^+]_{mit})$			

J_{NHE}; Mitochondrial Na^+/H^+ exchange flux

	Abbreviation	Value	Unit
	V_{max_NHE}	4.84155	$mM \cdot msec^{-1}$
	K_{Namit}	6.0	mM
	K_{Nacyt}	20.0	mM
	K_H	$1.0 \cdot 10^{-7}$	mM
	K_{Hreg}	$7.57 \cdot 10^{-5}$	mM

$$J_{NHE} = V_{max\_NHE} \cdot \left(\frac{\frac{[H^+]_{mit}}{[H^+]_{mit} + K_{Hreg}} \left(\frac{[Na^+]_{mit} \cdot [H^+]_{cyt}}{K_{Namit} \cdot K_H} - \frac{[Na^+]_{cyt} \cdot [H^+]_{mit}}{K_{Nacyt} \cdot K_H} \right)_{max}}{1 + \frac{[H^+]_{cyt}}{K_H} + \frac{[Na^+]_{mit}}{K_{Namit}} + \frac{[Na^+]_{mit} \cdot [H^+]_{cyt}}{K_{Namit} \cdot K_H} + \frac{[H^+]_{mit}}{K_H} + \frac{[Na^+]_{cyt}}{K_{Nacyt}} + \frac{[Na^+]_{cyt} \cdot [H^+]_{mit}}{K_{Nacyt} \cdot K_H}} \right)$$

Metabolite transporting system

J_{MCT}; Mitochondrial monocarboxylate transport flux

	Abbreviation	Value	Unit
	E_{total_MCT}	0.4	mM
	k_{catf}	$3.81 \cdot 10^{-3}$	$msec^{-1}$
	k_{catr}	$2.39 \cdot 10^{-3}$	$msec^{-1}$
	K_{mA}	$8.89 \cdot 10^{-6}$	mM
	K_{mB}	0.711	mM
	K_{mP}	0.659	mM
	K_{mQ}	$1.36 \cdot 10^{-6}$	mM
	α	0.14	
	β	1	
	γ	1	
	δ	1	

J_{MCT} $= E_{total\_MCT} \cdot \left(\frac{\frac{[A]\cdot[B]}{\alpha\cdot K_{mA}\cdot K_{mB}}\cdot k_{catf} - \frac{[P]\cdot[Q]}{\beta\cdot K_{mP}\cdot K_{mQ}}\cdot k_{catr}}{1+\frac{[A]}{K_{mA}}+\frac{[B]}{K_{mB}}+\frac{[P]}{K_{mP}}+\frac{[Q]}{K_{mQ}}+\frac{[A]\cdot[B]}{\alpha\cdot K_{mA}\cdot K_{mB}}+\frac{[P]\cdot[Q]}{\beta\cdot K_{mP}\cdot K_{mQ}}+\frac{[B]\cdot[Q]}{\gamma\cdot K_{mB}\cdot K_{mQ}}+\frac{[A]\cdot[P]}{\delta\cdot K_{mA}\cdot K_{mP}}} \right)$ where A = $[H^+]_{cyt}$, B = $[PYR]_{cyt}$, P = $[PYR]_{mit}$, Q = $[H^+]_{mit}$

J_{DCT}; Mitochondrial dicarboxylate transport flux

	Abbreviation	Value	Unit
	E_{total_DCT}	0.4	mM
	k_{catf}	$2.57\cdot 10^{-3}$	$msec^{-1}$
	k_{catr}	$2.90 \cdot 10^{-3}$	$msec^{-1}$
	K_{mA}	0.519	mM
	K_{mB}	0.670	mM
	K_{mP}	0.830	mM
	K_{mQ}	0.780	mM
	α	0.84	
	β	1	
	γ	1	
	δ	1	
J_{DCT} $= E_{total\_DCT} \cdot \left(\frac{\frac{[A]\cdot[B]}{\alpha\cdot K_{mA}\cdot K_{mB}}\cdot k_{catf} - \frac{[P]\cdot[Q]}{\beta\cdot K_{mP}\cdot K_{mQ}}\cdot k_{catr}}{1+\frac{[A]}{K_{mA}}+\frac{[B]}{K_{mB}}+\frac{[P]}{K_{mP}}+\frac{[Q]}{K_{mQ}}+\frac{[A]\cdot[B]}{\alpha\cdot K_{mA}\cdot K_{mB}}+\frac{[P]\cdot[Q]}{\beta\cdot K_{mP}\cdot K_{mQ}}+\frac{[B]\cdot[Q]}{\gamma\cdot K_{mB}\cdot K_{mQ}}+\frac{[A]\cdot[P]}{\delta\cdot K_{mA}\cdot K_{mP}}} \right)$ where A = $[MAL]_{cyt}$, B = $[Pi]_{mit}$, P = $[Pi]_{cyt}$, Q = $[MAL]_{mit}$			

J_{TCT}; Mitochondrial tricarboxylate transport flux

	Abbreviation	Value	Unit
	E_{total_TCT}	0.5	mM
	k_{catf}	$5.25\cdot 10^{-3}$	$msec^{-1}$
	k_{catr}	$5.75\cdot 10^{-3}$	$msec^{-1}$
	K_{mA}	0.039	mM
	K_{mB}	0.055	mM
	K_{mP}	0.35	mM
	K_{mQ}	0.042	mM
	α	1	

	β	1	
	γ	1	
	δ	1	

$$J_{TCT} = E_{total\_TCT} \cdot \left(\frac{\frac{[A]\cdot[B]}{\alpha\cdot K_{mA}\cdot K_{mB}}\cdot k_{catf} - \frac{[P]\cdot[Q]}{\beta\cdot K_{mP}\cdot K_{mQ}}\cdot k_{catr}}{1+\frac{[A]}{K_{mA}}+\frac{[B]}{K_{mB}}+\frac{[P]}{K_{mP}}+\frac{[Q]}{K_{mQ}}+\frac{[A]\cdot[B]}{\alpha\cdot K_{mA}\cdot K_{mB}}+\frac{[P]\cdot[Q]}{\beta\cdot K_{mP}\cdot K_{mQ}}+\frac{[B]\cdot[Q]}{\gamma\cdot K_{mB}\cdot K_{mQ}}+\frac{[A]\cdot[P]}{\delta\cdot K_{mA}\cdot K_{mP}}} \right)$$

where A = $[CIT]_{cyt}$, B = $[MAL]_{mit}$, P = $[MAL]_{cyt}$, Q = $[CIT]_{mit}$

J_{OGC}; Mitochondrial 2-oxoglutarate/malate carrier flux

	Abbreviation	Value	Unit
	E_{total_OGC}	0.272	mM
	k_{catf}	$4.36 \cdot 10^{-3}$	$msec^{-1}$
	k_{catr}	0.0169	$msec^{-1}$
	K_{mA}	0.035	mM
	K_{mB}	2.37	mM
	K_{mP}	0.21	mM
	K_{mQ}	1.0	mM
	α	0.65	
	β	1	
	γ	1	
	δ	1	

$$J_{OGC} = E_{total\_OGC} \cdot \left(\frac{\frac{[A]\cdot[B]}{\alpha\cdot K_{mA}\cdot K_{mB}}\cdot k_{catf} - \frac{[P]\cdot[Q]}{\beta\cdot K_{mP}\cdot K_{mQ}}\cdot k_{catr}}{1+\frac{[A]}{K_{mA}}+\frac{[B]}{K_{mB}}+\frac{[P]}{K_{mP}}+\frac{[Q]}{K_{mQ}}+\frac{[A]\cdot[B]}{\alpha\cdot K_{mA}\cdot K_{mB}}+\frac{[P]\cdot[Q]}{\beta\cdot K_{mP}\cdot K_{mQ}}+\frac{[B]\cdot[Q]}{\gamma\cdot K_{mB}\cdot K_{mQ}}+\frac{[A]\cdot[P]}{\delta\cdot K_{mA}\cdot K_{mP}}} \right)$$

where A = $[OG]_{cyt}$, B = $[MAL]_{mit}$, P = $[MAL]_{cyt}$, Q = $[OG]_{mit}$

J_{AGC}; Mitochondrial aspartate/glutamate carrier flux

	Abbreviation	Value	Unit
	E_{total_AGC}	2.0	mM
	k_{cat}	$2.74176 \cdot 10^{-5}$	$msec^{-1}$
	K_{mA}	0.0298	mM
	K_B	1.78	mM
	K_P	0.228	mM

	K_{mQ}	2.8	mM
	K_{HG}	3.16×10^{-4}	mM
	$\alpha_{\Delta\Psi}$	0.3713	
	K_{aCa}	$3.4218 \cdot 10^{-4}$	mM
	n_{Ca}	4.6	
	L_{Ca}	31.262	
	α	1.49	
	β	1	
	γ	1	
	δ	1	

$$k_{catf} = k_{cat} \cdot \left(1 + \frac{L_{Ca} \cdot [Ca^{2+}]_{cyt}^{nCa}}{[Ca^{2+}]_{cyt}^{nCa} + K_{aCa}^{nCa}}\right) \cdot \exp\left((1 - \alpha_{\Delta\Psi}) \cdot \Delta\Psi \cdot \frac{F}{RT}\right)$$

$$k_{catr} = k_{cat} \cdot \left(1 + \frac{L_{Ca} \cdot [Ca^{2+}]_{cyt}^{nCa}}{[Ca^{2+}]_{cyt}^{nCa} + K_{aCa}^{nCa}}\right) \cdot \exp\left(-\alpha_{\Delta\Psi} \cdot \Delta\Psi \cdot \frac{F}{RT}\right)$$

$$K_{mB} = \frac{K_B \cdot K_{HG}}{[H^+]_{mit}}$$

$$K_{mP} = \frac{K_P \cdot K_{HG}}{[H^+]_{cyt}}$$

$$J_{AGC} = E_{total\_AGC} \cdot \left(\frac{\frac{[A] \cdot [B]}{\alpha \cdot K_{mA} \cdot K_{mB}} \cdot k_{catf} - \frac{[P] \cdot [Q]}{\beta \cdot K_{mP} \cdot K_{mQ}} \cdot k_{catr}}{1 + \frac{[A]}{K_{mA}} + \frac{[B]}{K_{mB}} + \frac{[P]}{K_{mP}} + \frac{[Q]}{K_{mQ}} + \frac{[A] \cdot [B]}{\alpha \cdot K_{mA} \cdot K_{mB}} + \frac{[P] \cdot [Q]}{\beta \cdot K_{mP} \cdot K_{mQ}} + \frac{[B] \cdot [Q]}{\gamma \cdot K_{mB} \cdot K_{mQ}} + \frac{[A] \cdot [P]}{\delta \cdot K_{mA} \cdot K_{mP}}} \right)$$

where A = $[ASP]_{cyt}$, B = $[GLU]_{mit}$, P = $[GLU]_{cyt}$, Q = $[ASP]_{mit}$

Citric acid cycle

J_{CS}; Citrate synthase flux

	Abbreviation	Value	Unit
	E_{total_CS}	0.0552	mM
	k_{cat}	0.259	$msec^{-1}$
	K_H	$4.5 \cdot 10^{-5}$	mM
	K_{mA}	$6.45 \cdot 10^{-3}$	mM
	K_{mB}	$4.97 \cdot 10^{-3}$	mM
	K_{iATP}	0.518	mM
	K_{iADP}	1.42	mM
	K_{iScCoA}	0.130	mM
	K_{iCIT}	1.60	mM

	K_{iCoA}	0.067	mM

$$\alpha_1 = \frac{1}{1 + \frac{[H^+]_{mit}}{K_H}}$$

$$\alpha_2 = 1 + \frac{[CIT]_{mit}}{K_{iCIT}}$$

$$\alpha_3 = 1 + \frac{[CoA]_{mit}}{K_{iCoA}} + \frac{[ScCoA]_{mit}}{K_{iScCoA}} + \frac{[freeATP]_{mit}}{K_{iATP}} + \frac{[freeADP]_{mit}}{K_{iADP}}$$

$$J_{CS} = E_{total\_MCT} \cdot \left(\frac{k_{cat} \cdot [A] \cdot [B] \cdot \alpha_1}{[A] \cdot [B] + K_{mA} \cdot [B] \cdot \alpha_2 + K_{mB} \cdot [A] \cdot \alpha_3 + K_{mA} \cdot K_{mB} \cdot \alpha_2 \cdot \alpha_3} \right)$$

where A = $[OAA]_{mit}$, B = $[AcCoA]_{mit}$

J_{ACO}; Aconitase flux

	Abbreviation	Value	Unit
	E_{total_ACO}	1.0	mM
	k_{catf}	0.0723	$msec^{-1}$
	k_{catr}	0.0706	$msec^{-1}$
	K_{mA}	0.776	mM
	K_{mP}	0.0621	mM
	K_{ii}	1.45	mM
	K_{is}	0.547	mM

$$J_{ACO} = E_{total\_ACO} \cdot \left(\frac{k_{catf} \cdot K_{mP} \cdot [A] - k_{catr} \cdot K_{mA} \cdot [P]}{K_{mA} \cdot K_{mP} \cdot \left(1 + \frac{[OAA]^2}{K_{is}}\right) + (K_{mP} \cdot [A] + K_{mA} \cdot [P]) \cdot \left(1 + \frac{[OAA]^2}{K_{ii}}\right)} \right)$$

where A = $[CIT]_{mit}$, P = $[ISOC]_{mit}$

J_{ICDH}; NAD-dependent isocitrate dehydrogenase flux

	Abbreviation	Value	Unit
	E_{total_ICDH}	0.218	mM
	k_{cat}	0.148	$msec^{-1}$
	K_H	$2.88 \cdot 10^{-3}$	mM
	K_{mA}	0.131	mM
	K_{mB}	0.251	mM
	K_{iq}	$3.51 \cdot 10^{-3}$	mM
	K_{ADP}	0.614	mM
	α_{ADP}	0.024	
	β_{ADP}	0.86	

	K_{ATP}	1.78	mM
	α_{ATP}	0.25	
	β_{ATP}	0.86	
	K_{Ca1}	0.00388	mM
	α_{Ca1}	0.0011	
	β_{Ca1}	0.97	
	K_{Ca2}	0.0263	mM
	α_{Ca2}	0.027	
	β_{Ca2}	1.02	
	K_{iMg1}	0.698	mM
	K_{iMg2}	1.25	mM

$$Ca_{Act1\_1} = 1 + \frac{\alpha_{ADP} \cdot \beta_{Ca1} \cdot [Ca^{2+}]_{mit}}{\alpha_{Ca1} \cdot \beta_{ADP} \cdot K_{Ca1} \cdot \left(1 + \left(\frac{[Mg^{2+}]_{mit}}{K_{iMg2}}\right)^2\right)}$$

$$Ca_{Act1\_2} = 1 + \frac{\alpha_{ADP} \cdot [Ca^{2+}]_{mit}}{\alpha_{Ca1} \cdot K_{Ca1} \cdot \left(1 + \left(\frac{[Mg^{2+}]_{mit}}{K_{iMg2}}\right)^2\right)}$$

$$Ca_{Act1\_3} = 1 + \frac{[Ca^{2+}]_{mit}}{K_{Ca1} \cdot \left(1 + \left(\frac{[Mg^{2+}]_{mit}}{K_{iMg2}}\right)^2\right)}$$

$$Ca_{Act2\_1} = 1 + \frac{\alpha_{ATP} \cdot \beta_{Ca2} \cdot [Ca^{2+}]_{mit}}{\alpha_{Ca2} \cdot \beta_{ATP} \cdot K_{Ca2} \cdot \left(1 + \left(\frac{[Mg^{2+}]_{mit}}{K_{iMg2}}\right)^2\right)}$$

$$Ca_{Act2\_2} = 1 + \frac{\alpha_{ATP} \cdot [Ca^{2+}]_{mit}}{\alpha_{Ca2} \cdot K_{Ca2} \cdot \left(1 + \left(\frac{[Mg^{2+}]_{mit}}{K_{iMg2}}\right)^2\right)}$$

$$Ca_{Act2\_3} = 1 + \frac{[Ca^{2+}]_{mit}}{K_{Ca2} \cdot \left(1 + \left(\frac{[Mg^{2+}]_{mit}}{K_{iMg2}}\right)^2\right)}$$

$$\alpha_1 = \frac{\left(1 + \frac{\beta_{ADP} \cdot K_{mA} \cdot [totalADP]_{mit}}{\alpha_{ADP} \cdot K_{ADP} \cdot [A]} \cdot Ca_{Act1\_1} + \frac{\beta_{ATP} \cdot [totalATP]_{mit}}{\alpha_{ATP} \cdot K_{ATP}} \cdot Ca_{Act2\_1}\right)}{\left(1 + \frac{K_{mA} \cdot [totalADP]_{mit}}{\alpha_{ADP} \cdot K_{ADP} \cdot [A]} \cdot Ca_{Act1\_2} + \frac{[totalATP]_{mit}}{\alpha_{ATP} \cdot K_{ATP}} \cdot Ca_{Act2\_2}\right)}$$

$$\alpha_2 = \frac{\left(1 + \frac{K_{mA} \cdot [totalADP]_{mit}}{K_{ADP} \cdot [A]} \cdot Ca_{Act1\_3} + \frac{[totalATP]_{mit}}{K_{ATP}} \cdot Ca_{Act2\_3}\right)}{\left(1 + \frac{K_{mA} \cdot [totalADP]_{mit}}{\alpha_{ADP} \cdot K_{ADP} \cdot [A]} \cdot Ca_{Act1\_2} + \frac{[totalATP]_{mit}}{\alpha_{ATP} \cdot K_{ATP}} \cdot Ca_{Act2\_2}\right)} \cdot \left(1 + \frac{[Mg^{2+}]_{mit}}{K_{iMg1}}\right)$$

$$\alpha_3 = 1 + \frac{[Q]}{K_{iq}}$$

$$\alpha_H = \frac{1}{1 + \frac{[H^+]_{mit}}{K_H}}$$

$$J_{ICDH} = E_{total\_ICDH} \cdot \left(\frac{k_{cat} \cdot [A]^3 \cdot [B] \cdot \alpha_1 \cdot \alpha_H}{[A]^3 \cdot [B] + K_{mA}{}^3 \cdot [B] \cdot \alpha_2 + K_{mB} \cdot [A]^3 \cdot \alpha_3 + K_{mA}{}^3 \cdot K_{mB} \cdot \alpha_2 \cdot \alpha_3} \right)$$

where A = $[MgISOC]_{mit}$, B = $[NAD^+]_{mit}$, Q = $[NADH]_{mit}$

J_{OGDH}; 2-oxoglutarate dehydrogenase flux

	Abbreviation	Value	Unit
	E_{total_OGDH}	0.0196	mM
	k_{cat}	0.177	$msec^{-1}$
	K_{mA}	1.59	mM
	K_{mB}	0.0171	mM
	K_{mC}	0.0324	mM
	K_{mP}	0.3	mM
	K_{mR}	0.6	mM
	K_{ia}	0.72	mM
	K_{ib}	0.74	mM
	K_{ic}	0.1	mM^2
	K_{ip}	$1.1 \cdot 10^{-3}$	mM
	K_{iq}	0.0874	mM
	K_{ir}	$8.08 \cdot 10^{-3}$	mM
	K_{Mg}	0.0262	mM
	α_{Mg}	1.1	
	β_{Mg}	3.17	
	K_{Ca}	$3.0 \cdot 10^{-4}$	mM
	α_{Ca}	0.236	
	β_{Ca}	1.0	
	n_{Ca}	1.63	
	K_{ADP}	0.115	mM
	α_{ADP}	0.663	
	β_{ADP}	2.1	
	K_{Pi1}	1.2	mM
	α_{Pi1}	1.0	
	β_{Pi1}	1.58	
	n_{Pi1}	3.6	
	K_{Pi2}	10.8	mM

	α_{Pi2}	1.0	
	β_{Pi2}	1.27	
	n_{Pi2}	9.3	

$$Ca_{Act1} = 1 + \left(\frac{[Ca^{2+}]_{mit}}{K_{Ca}}\right)^{nCa}$$

$$Ca_{Act2} = 1 + \left(\frac{[Ca^{2+}]_{mit}}{\alpha_{Ca} \cdot K_{Ca}}\right)^{nCa}$$

$$Ca_{Act3} = 1 + \beta_{Ca} \cdot \left(\frac{[Ca^{2+}]_{mit}}{\alpha_{Ca} \cdot K_{Ca}}\right)^{nCa}$$

$$\alpha = \frac{Ca_{Act1} \cdot \left(1 + \frac{[Mg^{2+}]_{mit}}{K_{Mg}}\right) \cdot \left(1 + \frac{[totalADP]_{mit}}{K_{ADP}}\right) \cdot \left(1 + \left(\frac{[Pi]_{mit}}{K_{Pi1}}\right)^{nPi1}\right) \cdot \left(1 + \left(\frac{[Pi]_{mit}}{K_{Pi2}}\right)^{nPi2}\right)}{Ca_{Act2} \cdot \left(1 + \frac{[Mg^{2+}]_{mit}}{\alpha_{Mg} \cdot K_{Mg}}\right) \cdot \left(1 + \frac{[totalADP]_{mit}}{\alpha_{ADP} \cdot K_{ADP}}\right) \cdot \left(1 + \left(\frac{[Pi]_{mit}}{\alpha_{Pi1} \cdot K_{Pi1}}\right)^{nPi1}\right) \cdot \left(1 + \left(\frac{[Pi]_{mit}}{\alpha_{Pi2} \cdot K_{Pi2}}\right)^{nPi2}\right)}$$

$$\beta = \frac{Ca_{Act3} \cdot \left(1 + \frac{\beta_{Mg} \cdot [Mg^{2+}]_{mit}}{\alpha_{Mg} \cdot K_{Mg}}\right) \cdot \left(1 + \frac{\beta_{ADP} \cdot [totalADP]_{mit}}{\alpha_{ADP} \cdot K_{ADP}}\right) \cdot \left(1 + \beta_{Pi1} \cdot \left(\frac{[Pi]_{mit}}{\alpha_{Pi1} \cdot K_{Pi1}}\right)^{nPi1}\right) \cdot \left(1 + \beta_{Pi2} \cdot \left(\frac{[Pi]_{mit}}{\alpha_{Pi2} \cdot K_{Pi2}}\right)^{nPi2}\right)}{Ca_{Act2} \cdot \left(1 + \frac{[Mg^{2+}]_{mit}}{\alpha_{Mg} \cdot K_{Mg}}\right) \cdot \left(1 + \frac{[totalADP]_{mit}}{\alpha_{ADP} \cdot K_{ADP}}\right) \cdot \left(1 + \left(\frac{[Pi]_{mit}}{\alpha_{Pi1} \cdot K_{Pi1}}\right)^{nPi1}\right) \cdot \left(1 + \left(\frac{[Pi]_{mit}}{\alpha_{Pi2} \cdot K_{Pi2}}\right)^{nPi2}\right)}$$

$$\begin{aligned} denominator = {} & [A] \cdot [B] \cdot [C] + \alpha \cdot K_{mA} \cdot [B] \cdot [C] + K_{mB} \cdot [C] \cdot [A] + K_{mC} \cdot [A] \cdot [B] \\ & + \frac{\alpha \cdot K_{mA} \cdot K_{mP} \cdot K_{ib} \cdot K_{ic} \cdot [Q] \cdot [R]}{K_{ip} \cdot K_{iq} \cdot K_{mR}} + \frac{K_{mC} \cdot [A] \cdot [B] \cdot [R]}{K_{ir}} + \frac{K_{mB} \cdot [A] \cdot [C] \cdot [Q]}{K_{iq}} \\ & + \frac{\alpha \cdot K_{mA} \cdot K_{mP} \cdot K_{ib} \cdot K_{ic} \cdot [A] \cdot [Q] \cdot [R]}{K_{ia} \cdot K_{ip} \cdot K_{iq} \cdot K_{mR}} \end{aligned}$$

$$J_{OGDH} = E_{total\_OGDH} \cdot \left(\frac{k_{cat} \cdot [A] \cdot [B] \cdot [C] \cdot \beta}{denominator}\right)$$

where $A = [OG]_{mit}$, $B = [CoA]_{mit}$, $C = [NAD^{+}]_{mit}$, $Q = [ScCoA]_{mit}$, $R = [NADH]_{mit}$

J_{SCS}; Succinyl-CoA synthase flux

	Abbreviation	Value	Unit
	E_{total_SCS}	0.18	mM
	k_{cat1}	0.163	$msec^{-1}$
	k_{cat2}	0.00199	$msec^{-1}$
	K_{mA}	0.0556	mM
	K_{mB}	0.0245	mM
	K_{mC}	30.9	mM
	K_{mC2}	0.151	mM
	K_{mP2}	0.914	mM
	K_{mQ}	0.0212	mM

	K_{ia}	0.162	mM^2
	K_{ic}	0.704	mM
	K_{ip}	40.1	mM
	K_{iq}	0.0185	mM
	K_{ir}	0.0249	mM
	K_{eq}	7.605	

$$\text{numerator} = \left([A]\cdot[B]\cdot[C] - \frac{[P]\cdot[Q]\cdot[R]}{K_{eq}}\right)\cdot\left(k_{cat1} + k_{cat2}\cdot\left(\frac{K_{mC}\cdot[P]}{K_{mC2}\cdot K_{ip}} + \frac{[C]}{K_{mC2}}\right)\right)$$

$$\begin{aligned}\text{denominator} = {} & K_{ia}\cdot K_{mB}\cdot[C] + K_{mC}\cdot[A]\cdot[B] + K_{mA}\cdot[B]\cdot[C] + K_{mB}\cdot[C]\cdot[A] + [A]\cdot[B]\cdot[C] \\ & + \frac{[A]\cdot[B]\cdot[C]^2}{K_{mC2}} + \frac{K_{ia}\cdot K_{mB}\cdot K_{mC}\cdot[P]}{K_{ip}} + \frac{K_{ia}\cdot K_{mB}\cdot K_{mC}\cdot[P]\cdot[Q]}{K_{ip}\cdot K_{iq}} \\ & + \frac{K_{ia}\cdot K_{mB}\cdot K_{mC}\cdot[P]\cdot[R]}{K_{ip}\cdot K_{ir}} + \frac{K_{ia}\cdot K_{mB}\cdot K_{ic}\cdot[Q]\cdot[R]}{K_{mQ}\cdot K_{ir}} \\ & + \frac{K_{ia}\cdot K_{mB}\cdot K_{mC}[P]\cdot[Q]\cdot[R]}{K_{ip}\cdot K_{mQ}\cdot K_{ir}} + \frac{K_{ia}\cdot K_{mB}\cdot K_{mC}\cdot[P]^2\cdot[Q]\cdot[R]}{K_{mP2}\cdot K_{ip}\cdot K_{mQ}\cdot K_{ir}} \\ & + \frac{K_{ia}\cdot K_{mB}\cdot[C]\cdot[Q]}{K_{iq}} + \frac{K_{ia}\cdot K_{mB}\cdot[C]\cdot[R]}{K_{ir}} + \frac{K_{ia}\cdot K_{mB}\cdot[C]\cdot[Q]\cdot[R]}{K_{ir}\cdot K_{mQ}} \\ & + \frac{K_{ia}\cdot K_{mB}\cdot[C]\cdot[P]\cdot[Q]\cdot[R]}{K_{mP2}\cdot K_{ir}\cdot K_{mQ}} + \frac{K_{mB}\cdot K_{mC}\cdot[A]\cdot[P]}{K_{ip}} + \frac{K_{mA}\cdot K_{mC}\cdot[B]\cdot[P]}{K_{ip}} \\ & + \frac{K_{mC}\cdot[A]\cdot[B]\cdot[P]}{K_{ip}} + \frac{K_{mC}\cdot[A]\cdot[B]\cdot[C]\cdot[P]}{K_{mC2}\cdot K_{ip}} + \frac{K_{mA}\cdot[B]\cdot[C]\cdot[Q]}{K_{iq}} \\ & + \frac{K_{mB}\cdot[A]\cdot[C]\cdot[R]}{K_{ir}} + \frac{K_{mA}\cdot K_{mC}\cdot[B]\cdot[P]\cdot[Q]}{K_{ip}\cdot K_{iq}} + \frac{K_{mB}\cdot K_{mC}\cdot[A]\cdot[P]\cdot[R]}{K_{ip}\cdot K_{ir}}\end{aligned}$$

$$J_{SCS} = E_{total\_SCS}\cdot\left(\frac{\text{numerator}}{\text{denominator}}\right)$$

where $A = [GDP]_{mit}$, $B = [ScCoA]_{mit}$, $C = [Pi]_{mit}$, $P = [SUC]_{mit}$, $Q = [GTP]_{mit}$, $R = [CoA]_{mit}$

J_{SDH}; Succinate dehydrogenase flux

	Abbreviation	Value	Unit
	E_{total_SDH}	0.1328	mM
	k_{catf}	0.0783333	$msec^{-1}$
	k_{catr}	0.0019583	$msec^{-1}$
	K_{mA}	0.13	mM
	K_{mB}	$3.0\cdot 10^{-4}$	mM
	K_{mP}	0.025	mM
	K_{mQ}	$1.5\cdot 10^{-3}$	mM
	K_{ia}	0.03	mM
	K_{ip}	0.15	mM

	K_{iOAA1}	0.02	mM^2
	K_{iOAA2}	0.2	mM

$$J_{SDH} = E_{total\_SDH} \cdot \left(\frac{k_{catf}}{1 + \frac{K_{mA}}{[A]} \cdot \left(1 + \frac{[P]}{K_{ip}}\right) \cdot \left(1 + \frac{[OAA]_{mit}}{K_{iOAA1}}\right) + \frac{K_{mB}}{[B]}} - \frac{k_{catr}}{1 + \frac{K_{mP}}{[P]} \cdot \left(1 + \frac{[A]}{K_{ia}}\right) \cdot \left(1 + \frac{[OAA]_{mit}}{K_{iOAA2}}\right) + \frac{K_{mQ}}{[Q]}} \right)$$

where $A = [SUC]_{mit}$, $B = [UQ]_{mit}$, $P = [FUM]_{mit}$, $Q = [UQH_2]_{mit}$

J_{FH}; Fumarate hydratase flux

	Abbreviation	Value	Unit
	E_{total_FH}	0.2	mM
	k_{catf}	1.81667	$msec^{-1}$
	k_{catr}	1.31667	$msec^{-1}$
	K_A	$3.56 \cdot 10^{-3}$	mM
	K_P	0.0113	mM
	K_{iATP}	0.0115	mM
	K_{HA}	$2.64 \cdot 10^{-4}$	mM
	K_{HF}	0.06607	mM
	K_{HM}	0.01862	mM
	K_{aE}	$6.310 \cdot 10^{-4}$	mM
	K_{bE}	$1.584 \cdot 10^{-4}$	mM
	K_{aEF}	$5.012 \cdot 10^{-3}$	mM
	K_{bEF}	$5.012 \cdot 10^{-5}$	mM
	K_{aEM}	$2.512 \cdot 10^{-4}$	mM
	K_{bEM}	$3.981 \cdot 10^{-6}$	mM

$$K_{mA} = \frac{\left(1 + \frac{[H^+]_{mit}}{K_{a_E}} + \frac{K_{bE}}{[H^+]_{mit}}\right) \cdot \left(1 + \frac{[H^+]_{mit}}{K_{HF}}\right) \cdot K_A}{\left(1 + \frac{[H^+]_{mit}}{K_{a_{EF}}} + \frac{K_{bEF}}{[H^+]_{mit}}\right)}$$

$$K_{mP} = \frac{\left(1 + \frac{[H^+]_{mit}}{K_{a_E}} + \frac{K_{bE}}{[H^+]_{mit}}\right) \cdot \left(1 + \frac{[H^+]_{mit}}{K_{HM}}\right) \cdot K_P}{\left(1 + \frac{[H^+]_{mit}}{K_{a_{EM}}} + \frac{K_{bEM}}{[H^+]_{mit}}\right)}$$

$$J_{FH} = E_{total\_FH} \cdot \left(\frac{\dfrac{k_{catf}}{1 + \dfrac{[H^+]_{mit}}{K_{a_{EF}}} + \dfrac{K_{bEF}}{[H^+]_{mit}}} \cdot \dfrac{[A]}{K_{mA}} - \dfrac{k_{catr}}{1 + \dfrac{[H^+]_{mit}}{K_{a_{EM}}} + \dfrac{K_{bEM}}{[H^+]_{mit}}} \cdot \dfrac{[P]}{K_{mP}}}{\left(1 + \dfrac{[freeATP]_{mit}}{K_{iATP}} \cdot \left(1 + \dfrac{K_{HA}}{[H^+]_{mit}}\right)^{-1}\right) + \dfrac{[A]}{K_{mA}} + \dfrac{[P]}{K_{mP}}} \right)$$

where A = $[FUM]_{mit}$, P = $[MAL]_{mit}$

J_{MDH}; Malate dehydrogenase flux

	Abbreviation	Value	Unit
	E_{total_MDH}	0.644	mM
	k_{catf}	0.1177	$msec^{-1}$
	K_{mA}	0.0758	mM
	K_{mB}	0.498	mM
	K_{mP}	0.0391	mM
	K_{mQ}	0.111	mM
	K_{ia}	0.205	mM
	K_{ib}	0.860	mM
	K_{ip}	$4.02 \cdot 10^{-3}$	mM
	K_{iq}	0.0143	mM
	K_{CIT}	105.6	mM
	α_{CIT}	0.428	
	β_{CIT}	3.12	
	K_{iCIT}	15.3	mM
	K_{MAL}	3.0	mM
	α_{MAL}	9.97	
	β_{MAL}	1.78	
	K_{iOAA1}	5.51	mM
	K_{iOAA2}	6.26	mM
	K_{iATP}	0.1832	mM
	K_{iADP}	0.3944	mM
	K_{eq}	$1.36 \cdot 10^{-4}$	

$$\alpha_1 = 1 + \frac{[freeATP]_{mit}}{K_{iATP}} + \frac{[freeADP]_{mit}}{K_{iADP}}$$

$$\alpha_2 = \frac{1 + \dfrac{\beta_{CIT} \cdot [CIT]_{mit}}{\alpha_{CIT} \cdot K_{CIT}} + \dfrac{\beta_{MAL} \cdot [B]}{\alpha_{MAL} \cdot K_{MAL}}}{1 + \dfrac{[CIT]_{mit}}{\alpha_{CIT} \cdot K_{CIT}} + \dfrac{[B]}{\alpha_{MAL} \cdot K_{MAL}}}$$

$$\alpha_3 = \frac{1 + \frac{[\mathrm{CIT}]_{mit}}{K_{CIT}} + \frac{[B]}{K_{MAL}}}{1 + \frac{[\mathrm{CIT}]_{mit}}{\alpha_{CIT} \cdot K_{CIT}} + \frac{[B]}{\alpha_{MAL} \cdot K_{MAL}}}$$

$$\alpha_4 = \left(1 + \frac{[\mathrm{CIT}]_{mit}}{K_{iCIT}}\right) \cdot \left(1 + \frac{[P]}{K_{iOAA1}}\right)$$

$$\alpha_5 = 1 + \frac{[P]}{K_{iOAA2}}$$

$$\begin{aligned} \mathrm{denominator} = {} & K_{ia} \cdot K_{mB} \cdot \alpha_1 \cdot \alpha_3 + K_{mB} \cdot [A] \cdot \alpha_3 + K_{mA} \cdot [B] \cdot \alpha_1 + [A] \cdot [B] \\ & + \frac{K_{ia} \cdot K_{mB} \cdot K_{mQ} \cdot [P] \cdot \alpha_1 \cdot \alpha_3}{K_{mP} \cdot K_{iq}} + \frac{K_{ia} \cdot K_{mB} \cdot [Q] \cdot \alpha_3}{K_{iq} \cdot \alpha_4} + \frac{K_{mB} \cdot K_{mQ} \cdot [A] \cdot [P] \cdot \alpha_3}{K_{iq} \cdot K_{mP}} \\ & + \frac{K_{ia} \cdot K_{mB} \cdot [P] \cdot [Q] \cdot \alpha_3 \cdot \alpha_5}{K_{mP} \cdot K_{iq} \cdot \alpha_4} + \frac{K_{mA} \cdot [B] \cdot [Q]}{K_{iq} \cdot \alpha_4} + \frac{[A] \cdot [B] \cdot [P]}{K_{ip}} \\ & + \frac{K_{ia} \cdot K_{mB} \cdot [B] \cdot [P] \cdot [Q]}{K_{ib} \cdot K_{mP} \cdot K_{iq} \cdot \alpha_4} \end{aligned}$$

$$J_{MDH} = E_{total\_MDH} \cdot \frac{k_{catf} \cdot \left([A] \cdot [B] \cdot \alpha_2 - \frac{[P] \cdot [Q]}{K_{eq} \cdot \alpha_2 \cdot \alpha_4}\right)}{\mathrm{denominator}}$$

where $A = [NAD^+]_{mit}$, $B = [MAL]_{mit}$, $P = [OAA]_{mit}$, $Q = [NADH]_{mit}$

J_{NDK}; Nucleotide diphosphate kinase flux

	Abbreviation	Value	Unit
	E_{total_NDK}	0.2	mM
	k_{catf}	10.03	$msec^{-1}$
	K_{mA}	0.31	mM
	K_{mB}	0.043	mM
	K_{mP}	0.05	mM
	K_{ia}	0.21	mM
	K_{ib}	0.04	mM
	K_{ip}	0.057	mM
	K_{iq}	0.35	mM
	K_{eq}	1.28	

$$\begin{aligned} \mathrm{denominator} = {} & K_{mB} \cdot [A] + K_{mA} \cdot [B] + [A] \cdot [B] + \frac{K_{ia} \cdot K_{mB} \cdot [P]}{K_{ip}} + \frac{K_{mA} \cdot K_{ib} \cdot [Q]}{K_{iq}} \\ & + \frac{K_{mA} \cdot K_{ib} \cdot [P] \cdot [Q]}{K_{mP} \cdot K_{iq}} + \frac{K_{mB} \cdot [A] \cdot [P]}{K_{ip}} + \frac{K_{mA} \cdot [B] \cdot [Q]}{K_{iq}} \end{aligned}$$

$$J_{NDK} = E_{total\_NDK} \cdot \frac{k_{catf} \cdot \left([A] \cdot [B] - \frac{[P] \cdot [Q]}{K_{eq}}\right)}{\mathrm{denominator}}$$

where A = $[MgATP]_{mit}$, B = $[GDP]_{mit}$, P = $[MgADP]_{mit}$, Q = $[GTP]_{mit}$

J_{AST}; Aspartate amino transferase flux

	Abbreviation	Value	Unit
	E_{total_AST}	0.6	mM
	K_{cF}	0.300	$msec^{-1}$
	K_{cR}	0.580	$msec^{-1}$
	K_{mA}	1.58	mM
	K_{mB}	0.149	mM
	K_{mP}	0.0399	mM
	K_{mQ}	2.5	mM
	K_{ia}	2.0	mM
	K_{iq}	1.83	mM
	K_{eq}	0.113	

$$\text{denominator} = K_{cR}\cdot K_{mB}\cdot[A] + K_{cR}\cdot K_{mA}\cdot[B] + K_{cR}\cdot[A]\cdot[B] + \frac{K_{cF}\cdot K_{mQ}\cdot[P]}{K_{eq}} + \frac{K_{cF}\cdot K_{mP}\cdot[Q]}{K_{eq}} + \frac{K_{cF}\cdot K_{mQ}\cdot[A]\cdot[P]}{K_{ia}\cdot K_{eq}} + \frac{K_{cF}\cdot[P]\cdot[Q]}{K_{eq}} + \frac{K_{cR}\cdot K_{mA}\cdot[B]\cdot[Q]}{K_{iq}}$$

$$J_{AST} = E_{total\_AST}\cdot\frac{K_{cF}\cdot K_{cR}\cdot\left([A]\cdot[B] - \frac{[P]\cdot[Q]}{K_{eq}}\right)}{\text{denominator}}$$

where A = $[ASP]_{mit}$, B = $[OG]_{mit}$, P = $[OAA]_{mit}$, Q = $[GLU]_{mit}$

Pyruvate pathway

J_{PDHC}; Pyruvate dehydrogenase complex flux

	Abbreviation	Value	Unit
	E_{total_PDHC}	0.284	mM
	k_{cat}	0.308	$msec^{-1}$
	K_{mA}	0.025	mM
	K_{mB}	0.013	mM
	K_{mC}	0.050	mM
	K_{mP}	0.00059	mM
	K_{mR}	0.00069	mM
	K_{ia}	0.55	mM
	K_{ib}	0.30	mM
	K_{ic}	0.18	mM
	K_{ip}	0.060	mM

	K_{iq}	0.035	mM
	K_{ir}	0.036	mM
	u_1	13.84	
	u_2	0.03389	
	K_{Ca}	0.0002556	mM
	n_{Ca}	0.9497	

$$f_{PDHa} = \left(1 + u_2 \cdot \left(1 + \frac{u_1 \cdot {K_{Ca}}^{nCa}}{{K_{Ca}}^{nCa} + {[Ca^{2+}]_{mit}}^{nCa}}\right)\right)^{-1}$$

$$\begin{aligned}\text{denominator} = {} & [A] \cdot [B] \cdot [C] + K_{mA} \cdot [B] \cdot [C] + K_{mB} \cdot [C] \cdot [A] + K_{mC} \cdot [A] \cdot [B] \\ & + \frac{K_{mA} \cdot K_{mP} \cdot K_{ib} \cdot K_{ic} \cdot [Q] \cdot [R]}{K_{mR} \cdot K_{ip} \cdot K_{iq}} + \frac{K_{mC} \cdot [A] \cdot [B] \cdot [R]}{K_{ir}} + \frac{K_{mB} \cdot [C] \cdot [A] \cdot [Q]}{K_{iq}} \\ & + \frac{K_{mA} \cdot K_{mP} \cdot K_{ib} \cdot K_{ic} \cdot [A] \cdot [Q] \cdot [R]}{K_{mR} \cdot K_{ia} \cdot K_{ip} \cdot K_{iq}}\end{aligned}$$

$$J_{PDHC} = E_{total\_PDHC} \cdot f_{PDHa} \cdot \left(\frac{k_{cat} \cdot [A] \cdot [B] \cdot [C]}{\text{denominator}}\right)$$

where A = $[PYR]_{mit}$, B = $[CoA]_{mit}$, C = $[NAD^+]_{mit}$, Q = $[AcCoA]_{mit}$, R = $[NADH]_{mit}$

J_{PC}; Pyruvate carboxylase flux

	Abbreviation	Value	Unit
	E_{total_PC}	$2.0 \cdot 10^{-6}$	mM
	K_{cF}	0.07985	$msec^{-1}$
	K_{cR}	0.06861	$msec^{-1}$
	K_{mA}	0.207	mM
	K_{mB}	1.77	mM
	K_{mC}	0.693	mM
	K_{mP}	0.618	mM
	K_{mQ}	14.7	mM
	K_{mR}	0.0370	mM
	K_{ia}	0.138	mM
	K_{ib}	7.79	mM
	K_{ic}	0.0496	mM
	K_{ip}	0.190	mM
	K_{iq}	15.1	mM
	K_{ir}	0.167	mM
	K_{eq}	9.00	

$$\text{denominator} = K_{cR}\cdot K_{ia}\cdot K_{mB}\cdot[C] + K_{cR}\cdot K_{mC}\cdot[A]\cdot[B] + K_{cR}\cdot K_{mA}\cdot[B]\cdot[C] + K_{cR}\cdot K_{mB}\cdot[A]\cdot[C] + K_{cR}\cdot[A]\cdot[B]\cdot[C] + \frac{K_{cF}\cdot K_{ip}\cdot K_{mQ}\cdot[R]}{K_{eq}} + \frac{K_{cF}\cdot K_{mQ}\cdot[P]\cdot[R]}{K_{eq}} + \frac{K_{cF}\cdot K_{mP}\cdot[Q]\cdot[R]}{K_{eq}} + \frac{K_{cF}\cdot K_{mR}\cdot[P]\cdot[Q]}{K_{eq}} + \frac{K_{cF}\cdot[P]\cdot[Q]\cdot[R]}{K_{eq}} + \frac{K_{cR}\cdot K_{ia}\cdot K_{mB}\cdot[C]\cdot[P]}{K_{ip}} + \frac{K_{cR}\cdot K_{ia}\cdot K_{mB}\cdot[C]\cdot[Q]}{K_{iq}} + \frac{K_{cF}\cdot K_{mP}\cdot K_{iq}\cdot[B]\cdot[R]}{K_{ib}\cdot K_{eq}} + \frac{K_{cF}\cdot K_{mP}\cdot K_{iq}\cdot[A]\cdot[R]}{K_{ia}\cdot K_{eq}} + \frac{K_{cR}\cdot K_{mC}\cdot[A]\cdot[B]\cdot[R]}{K_{ir}} + \frac{K_{cF}\cdot K_{mR}\cdot[C]\cdot[P]\cdot[Q]}{K_{ic}\cdot K_{eq}} + \frac{K_{cR}\cdot K_{mA}\cdot[B]\cdot[C]\cdot[Q]}{K_{iq}} + \frac{K_{cR}\cdot K_{mA}\cdot[B]\cdot[C]\cdot[P]}{K_{ip}} + \frac{K_{cF}\cdot K_{mP}\cdot[B]\cdot[Q]\cdot[R]}{K_{ib}\cdot K_{eq}} + \frac{K_{cF}\cdot K_{mQ}\cdot[B]\cdot[P]\cdot[R]}{K_{ib}\cdot K_{eq}}$$

$$J_{PC} = E_{total\_PC}\cdot\frac{K_{cF}\cdot K_{cR}\cdot\left([A]\cdot[B]\cdot[C] - \frac{[P]\cdot[Q]\cdot[R]}{K_{eq}}\right)}{\text{denominator}}$$

where A = $[MgATP]_{mit}$, B = $[HCO_3^-]_{mit}$, C = $[PYR]_{mit}$, P = $[MgADP]_{mit}$, Q = $[Pi]_{mit}$, R = $[OAA]_{mit}$

J_{ALT}; Alanine aminotransferase flux

	Abbreviation	Value	Unit
	E_{total_ALT}	$2.0\cdot 10^{-6}$	mM
	k_{catf}	0.1088	$msec^{-1}$
	k_{catr}	0.1088	$msec^{-1}$
	K_{mA}	9.22	mM
	K_{mB}	0.115	mM
	K_{mP}	0.231	mM
	K_{mQ}	7.40	mM
	K_{ip}	0.192	mM
	K_{iq}	2.47	mM
	K_{IA}	555.0	mM
	K_{IQ}	97.4	mM
	K_{eq}	2.30	

$$\text{denominator} = k_{catr}\cdot K_{mA}\cdot[B] + k_{catr}\cdot K_{mB}\cdot[A] + k_{catr}\cdot[A]\cdot[B] + \frac{k_{catf}\cdot K_{mP}\cdot[Q]}{K_{eq}} + \frac{k_{catf}\cdot K_{mQ}\cdot[P]}{K_{eq}} + \frac{k_{catf}\cdot[P]\cdot[Q]}{K_{eq}} + \frac{k_{catr}\cdot K_{mA}\cdot[B]\cdot[Q]}{K_{iq}} + \frac{k_{catr}\cdot K_{mB}\cdot[A]\cdot[P]}{K_{ip}} + \frac{k_{catr}\cdot K_{mB}\cdot[A]^2}{K_{IA}} + \frac{k_{catf}\cdot K_{mP}\cdot[Q]^2}{K_{eq}\cdot K_{IQ}}$$

$$J_{ALT} = E_{total\_ALT} \cdot \frac{k_{catf} \cdot k_{catr} \cdot \left([A] \cdot [B] - \frac{[P] \cdot [Q]}{K_{eq}}\right)}{denominator}$$ where A = $[ALA]_{mit}$, B = $[OG]_{mit}$, P = $[PYR]_{mit}$, Q = $[GLU]_{mit}$

Table S10. Oxidative phosphorylation fluxes

	Abbreviation	Value	Unit
	k_{C1}	$2.2580 \cdot 10^{-4}$	$mM \cdot mV^{-1} \cdot msec^{-1}$
	k_{C3}	$1.25108 \cdot 10^{-5}$	$mM \cdot mV^{-1} \cdot msec^{-1}$
	k_{C4}	6.8037	$mM^{-1} \cdot msec^{-1}$
	K_{mO}	0.15	mM
	K_{mNADH}	0.014	mM
	K_{mUQ}	0.04	mM
	K_{mUQH2}	0.008	mM
	K_{mCytco}	0.0015	mM
	k_{SN}	$1.13146 \cdot 10^{-3}$	$mM \cdot msec^{-1}$
	n_{Ca1}	5.0	
	K_{Ca1}	$7.206 \cdot 10^{-5}$	mM
	L_1	4.0	
	ΔG_{p0}	31.9	$J \cdot mmol^{-1}$
	n_A	3.0	
	k_{ANT}	$8.52788 \cdot 10^{-3}$	$mM \cdot msec^{-1}$
	K_{ADP}	$3.5 \cdot 10^{-3}$	mM
	k_{PiC}	115.393	$mM \cdot msec^{-1}$
	pKa	6.8	
	k_{LK1}	0.0 ($7.710 \cdot 10^{-7}$ for isolated mitochondria)	$mM \cdot msec^{-1}$
	k_{LK2}	0.038	mM

$J_{ComplexI}$; Complex I of electron transport chain flux

$$\Delta G_{C1} = E_{mU} - E_{mN} - 2 \cdot \Delta p$$

$$\alpha = \frac{[NADH]_{mit} \cdot [UQ]_{mit}}{[NADH]_{mit} \cdot [UQ]_{mit} + [NADH]_{mit} \cdot K_{mUQ} + [UQ]_{mit} \cdot K_{mNADH}}$$

$$J_{ComplexI} = k_{C1} \cdot \Delta G_{C1} \cdot \alpha$$

$J_{ComplexIII}$; Complex III of electron transport chain flux

$$\Delta G_{C3} = E_{mc} - E_{mU} - 2 \cdot \Delta p - \Delta\Psi$$

$$\beta = \frac{[UQH_2]_{mit} \cdot [Cytco]_{mit}}{[UQH_2]_{mit} \cdot [Cytco]_{mit} + [Cytco]_{mit} \cdot K_{mUQH2} + [UQH_2]_{mit} \cdot K_{mCytco}}$$

$$J_{ComplexIII} = k_{C3} \cdot \Delta G_{C3} \cdot \beta$$

$J_{ComplexIV}$; Complex IV of electron transport chain flux

$$J_{ComplexIV} = k_{C4} \cdot [Cytar]_{mit} \cdot [Cytcr]_{mit} \cdot \frac{[O_2]}{[O_2] + K_{mO}}$$

$$mVO_2(mM \cdot min^{-1}) = 0.5 \cdot J_{ComplexIV} \cdot 1000 \cdot 60 \cdot \frac{Vol_{mit}}{Vol_i}$$

J_{ATPsyn}; F_1F_o-ATPase (ATP synthase) flux

$$\Delta G_p = 1000 \cdot \frac{\Delta G_p}{RT} + \ln\left(1000 \cdot \frac{[totalATP]_{mit}}{[totalADP]_{mit} \cdot [Pi]_{mit}}\right)$$

$$\Delta G_{SN} = \frac{n_A \cdot \Delta p \cdot F}{RT} - \Delta G_p$$

$$J_{ATPsyn} = k_{SN} \cdot \left(1 + \frac{L_1 \cdot [Ca^{2+}]_{mit}{}^{n_{Cai}}}{K_{Ca1}{}^{n_{Cai}} + [Ca^{2+}]_{mit}{}^{n_{Cai}}}\right) \cdot \left(\frac{\exp(\Delta G_{SN}) - 1}{\exp(\Delta G_{SN}) + 1}\right)$$

J_{ANT}; Adenine nucleotide translocase (ATP/ADP exchanger) flux

$$J_{ANT} = k_{ANT} \cdot \left(\frac{[freeADP]_{cyt}}{[freeADP]_{cyt} + K_{ADP}}\right) \cdot \left(\frac{[freeADP]_{cyt}}{[freeADP]_{cyt} + [freeATP]_{cyt} \cdot \exp\left(0.35 \cdot \Delta\psi \cdot \frac{F}{RT}\right)} - \frac{[freeADP]_{mit}}{[freeADP]_{mit} + [freeATP]_{mit} \cdot \exp\left(-0.65 \cdot \Delta\psi \cdot \frac{F}{RT}\right)}\right)$$

J_{PiC}; Phosphate carrier (PiC) flux

$$J_{PiC} = k_{PiC} \cdot \left(\frac{[Pi]_{cyt}}{1 + 10^{pH_{cyt} - pKa}} \cdot [H^+]_{cyt} - \frac{[Pi]_{mit}}{1 + 10^{pH_{mit} - pKa}} \cdot [H^+]_{mit}\right)$$

J_{HLeak}; Proton leak from cytoplasm to intramitochondria (Proton leak) flux

$$J_{HLeak} = k_{LK1} \cdot (\exp(k_{LK2} \cdot \Delta p) - 1)$$

Table S11. Nernst potentials

$$E_{Na\_JS} = \frac{RT}{F}\ln\frac{[Na^+]_o}{[Na^+]_{JS}}$$

$$E_{Na\_SL} = \frac{RT}{F}\ln\frac{[Na^+]_o}{[Na^+]_{SL}}$$

$$E_K = \frac{RT}{F}\ln\frac{[K^+]_o}{[K^+]_{cyt}}$$

$$E_{Ks\_JS} = \frac{RT}{F}\ln\left(\frac{[K^+]_o + pNaK \cdot [Na^+]_o}{[K^+]_{cyt} + pNaK \cdot [Na^+]_{JS}}\right)$$

$$E_{Ks\_SL} = \frac{RT}{F}\ln\left(\frac{[K^+]_o + pNaK \cdot [Na^+]_o}{[K^+]_{cyt} + pNaK \cdot [Na^+]_{SL}}\right)$$

$$E_{Ca\_JS} = \frac{RT}{2 \cdot F}\ln\frac{[Ca^{2+}]_o}{[Ca^{2+}]_{JS}}$$

$$E_{Ca\_SL} = \frac{RT}{2 \cdot F}\ln\frac{[Ca^{2+}]_o}{[Ca^{2+}]_{SL}}$$

$$E_{Cl} = -\frac{RT}{F}\ln\frac{[Cl^-]_o}{[Cl^-]_{cyt}}$$

Table S12. Ion/substrate concentrations

Junctional space

$$I_{Na\_totJS} = I_{Na\_JS} + I_{Nab\_JS} + 3 \cdot I_{NaK\_JS} + 3 \cdot I_{NCX\_JS} + I_{CaL\_NaJS}$$

$$I_{K\_totJS} = I_{Kr\_JS} + I_{Ks\_JS} + I_{Kp\_JS} + I_{to,f\_JS} + I_{to,s\_JS} + I_{K1\_JS} - 2 \cdot I_{NaK\_JS} + I_{CaL\_KJS}$$

$$I_{Ca\_totJS} = I_{CaL\_CaJS} + I_{pCa\_JS} + I_{Cab\_JS} - 2 \cdot I_{NCX\_JS}$$

$$I_{Cl\_totJS} = I_{ClCa\_JS} + I_{Clb\_JS}$$

Na$^+$ concentration

$$\frac{d[Na^+]_{JS}}{dt} = -I_{Na\_totJS} \cdot \frac{Cm}{Vol_{JS} \cdot F} + \frac{J_{Na\_JS\_SL}}{Vol_{JS}} - J_{NaB\_JS}$$

Ca^{2+} concentration

$$\frac{d[Ca^{2+}]_{JS}}{dt} = -I_{Ca\_totJS} \cdot \frac{Cm}{2 \cdot Vol_{JS} \cdot F} + \frac{J_{Ca\_JS\_SL} + J_{leak} \cdot Vol_{cyt} + J_{RyR} \cdot Vol_{SR} - J_{CaUni\_js} \cdot Vol_{mit}}{Vol_{JS}} - J_{CaB\_JS}$$

Subsarcolemmal space

$$I_{Na\_totSL} = I_{Na\_SL} + I_{Nab\_SL} + 3 \cdot I_{NaK\_SL} + 3 \cdot I_{NCX\_SL} + I_{CaL\_NaSL}$$

$$I_{K\_totSL} = I_{Kr\_SL} + I_{Ks\_SL} + I_{Kp\_SL} + I_{to,f\_SL} + I_{to,s\_SL} + I_{K1\_SL} - 2 \cdot I_{NaK\_SL} + I_{CaL\_KSL}$$

$$I_{Ca\_totSL} = I_{CaL\_CaSL} + I_{pCa\_SL} + I_{Cab\_SL} - 2 \cdot I_{NCX\_SL}$$

$$I_{Cl\_totSL} = I_{ClCa\_SL} + I_{Clb\_SL}$$

Na$^+$ concentration

$$\frac{d[Na^+]_{SL}}{dt} = -I_{Na\_totSL} \cdot \frac{Cm}{Vol_{SL} \cdot F} - \frac{J_{Na\_JS\_SL} + J_{Na\_cyt\_SL}}{Vol_{SL}} - J_{NaB\_SL}$$

Ca^{2+} concentration

$$\frac{d[Ca^{2+}]_{SL}}{dt} = -I_{Ca\_totSL} \cdot \frac{Cm}{2 \cdot Vol_{SL} \cdot F} - \frac{J_{Ca\_JS\_SL} + J_{Ca\_cyt\_SL}}{Vol_{SL}} - J_{CaB\_SL}$$

SR

Ca^{2+} concentration

$$\frac{d[Ca^{2+}]_{SR}}{dt} = J_{SERCA} - J_{RyR} - \left(\frac{J_{leak} \cdot Vol_{cyt} + J_{NmSC} \cdot Vol_{mit}}{Vol_{SR}}\right) - J_{Csqn}$$

Cytoplasmic space

Na^+ concentration

$$\frac{d[Na^+]_{cyt}}{dt} = \frac{J_{Na\_cyt\_SL}}{Vol_{cyt}} + \left(3 \cdot J_{NmSC} + 3 \cdot J_{NCXmit_{cyt}} + J_{NHE}\right) \cdot \frac{Vol_{mit}}{Vol_{cyt}}$$

K^+ concentration

$$\frac{d[K^+]_{cyt}}{dt} = 0$$

Ca^{2+} concentration

$$\frac{d[Ca^{2+}]_{cyt}}{dt} = -\frac{J_{SERCA} \cdot Vol_{SL} + J_{CaUni_{cyt}} \cdot Vol_{mit} + J_{NCXmit\_cyt} \cdot Vol_{mit} - J_{Ca\_cyt\_SL}}{Vol_{cyt}} + J_{Ca\_troponin} - J_{CaB\_total}$$

Cl^- concentration

$$\frac{d[Cl^-]_{cyt}}{dt} = 0$$

Energetics-related equations

$$J_{ATPcons} = \frac{dATPuse\_contraction}{dt} + \frac{dATPuse\_I_{NaK}}{dt} + \frac{dATPuse\_I_{pCa}}{dt} + \frac{dATPuse\_SERCA}{dt} + \frac{dATPuse\_NmSC}{dt}$$

$$\frac{d[H^+]_{cyt}}{dt} = 0$$

$$\frac{d[Pi]_{cyt}}{dt} = (-J_{PiC} + J_{DCT}) \cdot \frac{Vol_{mit}}{Vol_{cyt} + Vol_{JS} + Vol_{SL}} + J_{ATPcons}$$

$$\frac{d[totalATP]_{cyt}}{dt} = J_{ANT} \cdot \frac{Vol_{mit}}{Vol_{cyt} + Vol_{JS} + Vol_{SL}} + J_{AK} + J_{CK} - J_{ATPcons}$$

$$\frac{d[\text{totalADP}]_{\text{cyt}}}{dt} = -J_{\text{ANT}} \cdot \frac{\text{Vol}_{\text{mit}}}{\text{Vol}_{\text{cyt}} + \text{Vol}_{\text{JS}} + \text{Vol}_{\text{SL}}} - 2 \cdot J_{\text{AK}} - J_{\text{CK}} + J_{\text{ATPcons}}$$

$$\frac{d[\text{PCr}]_{\text{cyt}}}{dt} = -J_{\text{CK}}$$

$$[\text{freeATP}]_{\text{cyt}} = \frac{[\text{totalATP}]_{\text{cyt}}}{1 + \frac{[\text{Mg}^{2+}]_{\text{cyt}}}{K_{\text{dATPcyt}}}}$$

$$[\text{MgATP}]_{\text{cyt}} = [\text{totalATP}]_{\text{cyt}} - [\text{freeATP}]_{\text{cyt}}$$

$$[\text{freeADP}]_{\text{cyt}} = \frac{[\text{totalADP}]_{\text{cyt}}}{1 + \frac{[\text{Mg}^{2+}]_{\text{cyt}}}{K_{\text{dADPcyt}}}}$$

$$[\text{MgADP}]_{\text{cyt}} = [\text{totalADP}]_{\text{cyt}} - [\text{freeADP}]_{\text{cyt}}$$

$$[\text{AMP}]_{\text{cyt}} = [\text{totalAdenine}]_{\text{cyt}} - \left([\text{totalATP}]_{\text{cyt}} + [\text{totalADP}]_{\text{cyt}}\right)$$

$$[\text{Cr}]_{\text{cyt}} = [\text{totalCr}]_{\text{cyt}} - [\text{PCr}]_{\text{cyt}}$$

Mitochondria

Na^+ concentration

$$\frac{d[\text{Na}^+]_{\text{mit}}}{dt} = -\left(3 \cdot J_{\text{NmSC}} + 3 \cdot J_{\text{NCXmit}_{\text{cyt}}} + J_{\text{NHE}}\right)$$

K^+ concentration

$$\frac{d[\text{K}^+]_{\text{mit}}}{dt} = J_{\text{KUni}} - J_{\text{KHE}}$$

Ca^{2+} concentration

$$\frac{d[\text{Ca}^{2+}]_{\text{totalmit}}}{dt} = J_{\text{CaUni\_JS}} + J_{\text{CaUni\_cyt}} + J_{\text{NmSC}} + J_{\text{NCXmit\_cyt}}$$

$$[\text{Ca}^{2+}]_{\text{mit}} = 0.5 \cdot \Big(-(K_{\text{dCabuffmit}} + B_{\text{maxCamit}} - [\text{Ca}^{2+}]_{\text{totalmit}}) + \sqrt{(K_{\text{dCabuffmit}} + B_{\text{maxCamit}} - [\text{Ca}^{2+}]_{\text{totalmit}})^2 - 4 \cdot (-K_{\text{dCabuffmit}} \cdot [\text{Ca}^{2+}]_{\text{totalmit}})}\Big)$$

Energetics-related equations

$$\frac{d\Delta\Psi}{dt} = \Big(-4 \cdot J_{\text{ComplexI}} - 2 \cdot J_{\text{ComplexIII}} - 4 \cdot J_{\text{ComplexIV}} + n_A \cdot J_{\text{ATPsyn}} + J_{\text{ANT}} + J_{\text{HLeak}} - \left(J_{\text{NmSC}} + J_{\text{NCXmit\_cyt}}\right) + 2 \cdot \left(J_{\text{CaUni}_{\text{JS}}} + J_{\text{CaUni}_{\text{cyt}}}\right) + J_{\text{KUni}} - J_{\text{AGC}}\Big) / C_{\text{mit}}$$

$$pH_{mit} = \log\left(\frac{[H^+]_{mit}}{1000}\right)$$

$$rbuffer_{mit} = \frac{0.22}{\left(\frac{10^{-pH_{mit}} - 10^{-pH_{mit}-0.001}}{0.001}\right)}$$

$$\frac{d[H^+]_{mit}}{dt} = \left(-(4+1)\cdot J_{ComplexI} - 2\cdot J_{ComplexIII} - 4\cdot J_{ComplexIV} + (n_A - 1)\cdot J_{ATPsyn} + 2\cdot J_{PiC} + J_{HLeak} + J_{KHE} + J_{NHE} - J_{AGC} + J_{MCT} + J_{TCT} + J_{CS} + J_{MDH}\right)/rbuffer_{mit}$$

$$\frac{d[Pi]_{mit}}{dt} = J_{PiC} + J_{PC} - J_{ATPsyn} - J_{DCT} - J_{SCS}$$

$$\frac{d[totalATP]_{mit}}{dt} = J_{ATPsyn} - J_{ANT} - J_{PC} - J_{NDK}$$

$$\frac{d[NADH]_{mit}}{dt} = -J_{ComplexI} + J_{PDHC} + J_{ICDH} + J_{OGDH} + J_{MDH}$$

$$\frac{d[UQH_2]_{mit}}{dt} = J_{ComplexI} - J_{ComplexIII} + J_{SDH}$$

$$\frac{d[Cytcr]_{mit}}{dt} = 2\cdot\left(J_{ComplexIII} - J_{ComplexIV}\right)$$

$$\frac{d[ASP]_{mit}}{dt} = J_{AGC} - J_{AST}$$

$$\frac{d[GLU]_{mit}}{dt} = J_{ALT} + J_{AST} - J_{AGC}$$

$$\frac{d[OG]_{mit}}{dt} = J_{OGC} + J_{ICDH} - J_{OGDH} - J_{ALT} - J_{AST}$$

$$\frac{d[MAL]_{mit}}{dt} = J_{DCT} + J_{FH} - J_{OGC} - J_{TCT} - J_{MDH}$$

$$\frac{d[PYR]_{mit}}{dt} = J_{MCT} + J_{ALT} - J_{PDHC} - J_{PC}$$

$$\frac{d[CIT]_{mit}}{dt} = J_{TCT} + J_{CS} - J_{ACO}$$

$$\frac{d[ISOC]_{mit}}{dt} = J_{ACO} - J_{ICDH}$$

$$\frac{d[ScCoA]_{mit}}{dt} = J_{OGDH} - J_{SCS}$$

$$\frac{d[FUM]_{mit}}{dt} = J_{SDH} - J_{FH}$$

$$\frac{d[SUC]_{mit}}{dt} = J_{SCS} - J_{SDH}$$

$$\frac{d[OAA]_{mit}}{dt} = J_{PC} + J_{MDH} + J_{AST} - J_{CS}$$

$$\frac{d[AcCoA]_{mit}}{dt} = J_{PDHC} - J_{CS}$$

$$\frac{d[GTP]_{mit}}{dt} = J_{SCS} + J_{NDK}$$

$$\Delta pH = 2.3 \cdot \frac{RT}{F}\left(pH_{mit} - pH_{cyt}\right)$$

$$\Delta p = \Delta pH - \Delta\Psi$$

$$[freeATP]_{mit} = \frac{[totalATP]_{mit}}{1 + \frac{[Mg^{2+}]_{mit}}{K_{dATPmit}}}$$

$$[MgATP]_{mit} = [totalATP]_{mit} - [freeATP]_{mit}$$

$$[totalADP]_{mit} = [totalAdenine]_{mit} - [totalATP]_{mit}$$

$$[freeADP]_{mit} = \frac{[totalADP]_{mit}}{1 + \frac{[Mg^{2+}]_{mit}}{K_{dADPmit}}}$$

$$[MgADP]_{mit} = [totalADP]_{mit} - [freeADP]_{mit}$$

$$E_{mN} = E_{mN,0} + \frac{RT}{2 \cdot F} \cdot \ln\left(\frac{[NAD^{+}]_{mit}}{[NADH]_{mit}}\right)$$

$$E_{mU} = E_{mU,0} + \frac{RT}{2 \cdot F} \cdot \ln\left(\frac{[UQ]_{mit}}{[UQH_2]_{mit}}\right)$$

$$E_{mc} = E_{mc,0} + \frac{RT}{F} \cdot \ln\left(\frac{[Cytco]_{mit}}{[Cytcr]_{mit}}\right)$$

$$E_{ma} = E_{mc} + \Delta p - \Delta\Psi$$

$$[NAD^{+}]_{mit} = [totaNAD]_{mit} - [NADH]_{mit}$$

$$[UQ]_{mit} = [totaUQ]_{mit} - [UQH_2]_{mit}$$

$$[Cytco]_{mit} = [totaCytc]_{mit} - [Cytcr]_{mit}$$

$$[Cytar]_{mit} = \frac{[totaCyta]_{mit}}{1 + \exp\left(\left(E_{ma} - E_{ma,0}\right) \cdot \frac{F}{RT}\right)}$$

$$[Cytao]_{mit} = [totaCyta]_{mit} - [Cytar]_{mit}$$

$$[CoA]_{mit} = [totaCoA]_{mit} - [AcCoA]_{mit} - [ScCoA]_{mit}$$

$$[GDP]_{mit} = [totaGuanine]_{mit} - [GTP]_{mit}$$

$$[freeISOC]_{mit} = \frac{[ISOC]_{mit}}{1 + \frac{[Mg^{2+}]_{mit}}{K_{dISOC}}}$$

$$[MgISOC]_{mit} = [ISOC]_{mit} - [freeISOC]_{mit}$$

Table S13. Membrane potential

$$I_{Na\_tot} = I_{Na\_totJS} + I_{Na\_totSL}$$
$$I_{K\_tot} = I_{K\_totJS} + I_{K\_totSL}$$
$$I_{Ca\_tot} = I_{Ca\_totJS} + I_{Ca\_totSL}$$
$$I_{Cl\_tot} = I_{Cl\_totJS} + I_{Cl\_totSL}$$
$$I_{tot} = I_{Na\_tot} + I_{K\_tot} + I_{Ca\_tot} + I_{Cl\_tot}$$
$$\frac{dVm}{dt} = -I_{tot} + I_{stim}$$

Table S14. Steady state initial conditions; isotonic contraction, cycle length 1 sec

	MSI model	non-MSI model
Vm (mV)	−81.2558892292957	−81.3101273968024
ΔΨ (mV)	−180.490096455412	−183.506771485269
hsmL (μm)	0.950094340705532	0.956261997841713
hsmX (μm)	0.945104404607623	0.951261552233556
$[Ca^{2+}]_{cyt}$ (mM)	0.000104535415906831	$8.09659085389541 \cdot 10^{-5}$
$[Ca^{2+}]_{JS}$ (mM)	0.000229175103379173	0.000249856871687648
$[Ca^{2+}]_{SL}$ (mM)	0.00011936787903103	0.000110529335656894
$[Ca^{2+}]_{SR}$ (mM)	0.663050599960056	0.522965444051458
$[Ca^{2+}]_{totalmit}$ (mM)	0.405916100937862	0.0704741302887895
$[H^{+}]_{mit}$ (mM)	$3.43310110405974 \cdot 10^{-5}$	$3.29667017388503 \cdot 10^{-5}$
$[K^{+}]_{mit}$ (mM)	149.689472348037	150.446139107484
$[Na^{+}]_{cyt}$ (mM)	8.35661283258966	8.41740318417665
$[Na^{+}]_{JS}$ (mM)	8.36014328486369	8.42179680371618
$[Na^{+}]_{SL}$ (mM)	8.35651980486934	8.41728197019088
$[Na^{+}]_{mit}$ (mM)	1.71987233382287	1.66145734768444
$[CaB_{JS\_low}]$ (mM)	0.00960678487054338	0.0104608046703138
$[CaB_{JS\_high}]$ (mM)	0.0857767819084825	0.0904246435220084
$[CaB_{SL\_low}]$ (mM)	0.011057553484444	0.0102475842101007
$[CaB_{SL\_high}]$ (mM)	0.123520176557396	0.117322442042261
$[NaB_{JS}]$ (mM)	3.44324738149926	3.45734957920537
$[NaB_{SL}]$ (mM)	0.751250766264635	0.754215458785412
$[TnC_{JhCa}]$ (mM)	0.119610238614854	0.113295664355079
$[TnC_{JhMg}]$ (mM)	0.00957209070041661	0.012589546165631
[CaM] (mM)	0.000352764030215123	0.000274438930559555
$[Myocin_{Ca}]$ (mM)	0.00212636404565329	0.00158130342550922

$[Myocin_{Mg}]$ (mM)		0.137358876123565	0.137907835562763
[SRB] (mM)		0.00252934245904414	0.00203354675395066
[TCa] (mM)		0.00721215171157938	0.00590368249118565
$[TCa^{*}]$ (mM)		0.00029110445899942	0.000165499748064044
$[T^{*}]$ (mM)		$2.34412880633487 \cdot 10^{-5}$	$1.544343053913 \cdot 10^{-5}$
$[Csqn_{b}]$ (mM)		1.31291929083407	1.15920736119173
$[AcCoA]_{mit}$ (mM)		0.298693587730975	0.298933210419957
$[ScCoA]_{mit}$ (mM)		0.000639385031260494	0.000374672063197351
$[totalADP]_{cyt}$ (mM)		0.0180206051518157	0.0167114371119949
$[ASP]_{mit}$ (mM)		0.00416627361249721	0.00390257149053709
$[totalATP]_{cyt}$ (mM)		6.68161865850406	6.68297839940339
$[totalATP]_{mit}$ (mM)		6.73800885645313	5.8104365531558
$[CIT]_{mit}$ (mM)		1.04831653101393	1.07570723252622
$[Cytcr]_{mit}$ (mM)		0.0473357880696593	0.0448113894155385
$[FUM]_{mit}$ (mM)		0.115763416083576	0.117535639965651
$[GLU]_{mit}$ (mM)		1.20799329504835	1.20617790479048
$[GTP]_{mit}$ (mM)		0.601695985707051	0.542746339180489
$[ISOC]_{mit}$ (mM)		0.0857784921938939	0.0880995093446045
$[MAL]_{mit}$ (mM)		0.507464589209632	0.5152737442167
$[NADH]_{mit}$ (mM)		1.18753724965769	1.41570202237165
$[OAA]_{mit}$ (mM)		0.000122032447831617	$9.10837869219534 \cdot 10^{-5}$
$[OG]_{mit}$ (mM)		0.313091908391165	0.249123581979973
$[Pi^{-}]_{cyt}$ (mM)		0.365847720455209	0.239248578305876
$[Pi^{-}]_{mit}$ (mM)		0.0986638686436536	0.0654574795878746
$[PCr]_{cyt}$ (mM)		15.5555684113826	16.0305240611554
$[PYR]_{mit}$ (mM)		0.509695128610508	0.579999738302158
$[SUC]_{mit}$ (mM)		0.00535213170390013	0.00515877082323413
$[UQH_2]_{mit}$ (mM)		0.150593675397626	0.117487719513052
I_{Na_JS}	m	0.00395340673967454	0.00390914333987568
	h	0.619340289223851	0.621128543989574
	j	0.618411707293565	0.619266906286702
I_{Na_SL}	m	0.00395340673967454	0.00390914333987568
	h	0.619340289223851	0.621128543989574
	j	0.618411707293565	0.619266906286702
I_{Kr_JS}	xkr	0.0217569972741135	0.0235435419775075
I_{Kr_SL}	xkr	0.0217569972741135	0.0235435419775075
I_{Ks_JS}	xks	0.00433981340877934	0.00432371315389125

I_{Ks_SL}	xks	0.00433981340877934	0.00432371315389125
I_{to,f_JS}	xto,f	0.000447175822264377	0.000445367930895831
	yto,f	0.999995677077432	0.999995722029957
I_{to,f_SL}	xto,f	0.000447175822264377	0.000445367930895831
	yto,f	0.999995677077432	0.999995722029957
I_{to,s_JS}	xto,s	0.000447138035233467	0.000445367777073242
	yto,s	0.774771956121754	0.775083546170086
I_{to,s_SL}	xto,s	0.000447138035233467	0.000445367777073242
	yto,s	0.774771956121754	0.775083546170086
I_{CaL_JS}	d	$3.02277401395629\cdot 10^{-6}$	$2.9956249671874\cdot 10^{-6}$
	f	0.99502716467496	0.9950515753119
	fCaB	0.03071604355324	0.0345364790966573
I_{CaL_SL}	d	$3.02277401395629\cdot 10^{-6}$	$2.9956249671874\cdot 10^{-6}$
	f	0.99502716467496	0.9950515753119
	fCaB	0.0165623068383975	0.0156486491346093
J_{RyR}	RyR_r	0.888955281847854	0.848438046180244
	RyR_o	$1.56931208186383\cdot 10^{-6}$	$1.3179574510164\cdot 10^{-6}$
	RyR_i	$1.9602903656745\cdot 10^{-7}$	$2.35432816720526\cdot 10^{-7}$
